# Biocoin: an open-source wearable platform for multiplexed and multimodal biosensing

**DOI:** 10.1038/s44328-026-00103-z

**Published:** 2026-05-27

**Authors:** Tyler Hack, Drew A. Hall

**Affiliations:** 1 Department of Electrical and Computer Engineering, University of California—San Diego, La Jolla, CA USA; 2 Department of Bioengineering, University of California—San Diego, La Jolla, CA USA

**Keywords:** Biological techniques, Biotechnology, Engineering, Materials science, Nanoscience and technology

## Abstract

Wearable biosensors promise proactive, precision medicine by enabling continuous biochemical monitoring, yet their clinical translation remains hindered by high-performance hardware accessibility barriers. Practical on-body operation demands stringent miniaturization, multiplexing, multi-method sensing, iontophoresis, and ultra-low power wireless operation – challenges that force researchers to remain in vitro or engineer tailored hardware. Unfortunately, prior systems rarely satisfy these requirements simultaneously, proliferating inadaptable designs that prevent reuse across applications. We introduce Biocoin, the first fully open-source, open-hardware wearable biosensing platform that unifies these needs in a reconfigurable, general-purpose framework. The 530 mm^2^ wireless device demonstrates >10 multiplexed sensor inputs, broad physicochemical sensing (amperometry, voltammetry, potentiometry, impedimetry, temperature) with benchtop-comparable performance, current-monitored iontophoresis for biofluid extraction, and extensive power optimizations enabling months-long, µA-level multimodal sensing. All hardware, firmware, and software are publicly released, providing a turnkey platform that removes long-standing hardware barriers to on-body biosensor validation and accelerates their translation into clinically actionable wearables.

## Introduction

Wearable biosensors are poised to transform modern healthcare systems^1–4^, providing molecular-level health insights through continuous, non-invasive monitoring of sweat or interstitial fluid (ISF) biomarkers^5–8^. Ranging from wristbands to epidermal patches^9–16^, recent progress in skin-interfaced biochemical devices integrating microneedle arrays, sweat microfluidics, and iontophoresis has made on-demand access to these biofluids increasingly practical^17–20^. Electrochemical biosensors, pairing target-specific bioreceptors with rapid, high-sensitivity transduction methods^21–23^, have successfully quantified numerous physiologically and metabolically relevant markers abundant in these fluids, including electrolytes, metabolites, proteins, and nutrients^24–32^. Notably, many of these biomarkers correlate strongly with their blood counterparts, establishing their diagnostic credibility^5,19,33^. Such real-time physicochemical insights offer a path toward personalized, context-aware medicine by enabling timely interventions, proactive treatment, and early abnormality detection—a dramatic shift from traditional reactive healthcare constrained by delayed laboratory diagnostics^33^. However, despite these advances, translating biosensors from in vitro demonstrations to human studies remains slow, in part because researchers lack accessible, high-performance wearable hardware capable of on-body biofluid analysis.

The gap persists as a repercussion of the rigorous electronic and system-level requirements for reliable on-body operation. Comprising a power source, sensor(s), and measurement circuitry, these platforms must support multiplexed biomarker analysis for holistic health assessment and multi-method electrochemical techniques (amperometry, voltammetry, potentiometry, impedimetry) to accommodate diverse sensing mechanisms^34^. Critically, they must also function wirelessly with ultra-low power consumption, integrate iontophoresis for biofluid extraction or drug delivery, and possess a miniaturized, skin-compatible form factor without compromising measurement precision. Obtaining such systems necessitates multidisciplinary collaboration or deep expertise in mixed-signal circuit design, embedded firmware, and wireless communication—resources that many biosensor research institutions lack. Meanwhile, existing wearable hardware is typically proprietary, siloed behind barriers that impede adoption or modification. These constraints (demanding electronics and platform inaccessibility) have strongly influenced the biosensor literature landscape, accentuating two primary consequences. The first is a vast body of publications that is entirely restricted to artificial biofluid experimentation. Second, researchers who attempt on-body studies often introduce application-specific hardware that is insufficiently adaptable and fails to comprehensively address the electronics requirements, forcing redesign efforts for each new sensor and preventing hardware reuse across applications despite strikingly consistent needs. This redundancy, coupled with the absence of a unified hardware platform, hampers efficiency, limits replication and scaling of promising technologies, and confines sensor validation to in vitro lab demonstrations rather than clinical trials.

Open-source technologies have ushered in an era of accelerated scientific innovation by lowering barriers to cross-disciplinary collaboration^35–37^. Prominent ecosystems like Arduino and Linux exemplify how accessibility enables developers to build upon established foundations rather than reinvent systems. Prior open-source potentiostat devices^38–46^, though valuable as handheld, low-cost alternatives to benchtop electrochemical workstations, lack the essential multiplexing, power efficiency, and iontophoresis capabilities required for on-body operation. Unfortunately, among wearable biochemical devices, design releases have been sparse. Partial schematics, firmware fragments, and isolated component lists provide insufficient completeness for replication, proliferating the development of bespoke electronics that ultimately slows translation^15,47–49^. Subsequently, the demand for a transparent, ready-to-use, general-purpose biosensor solution remains unmet.

**Fig. 1:**
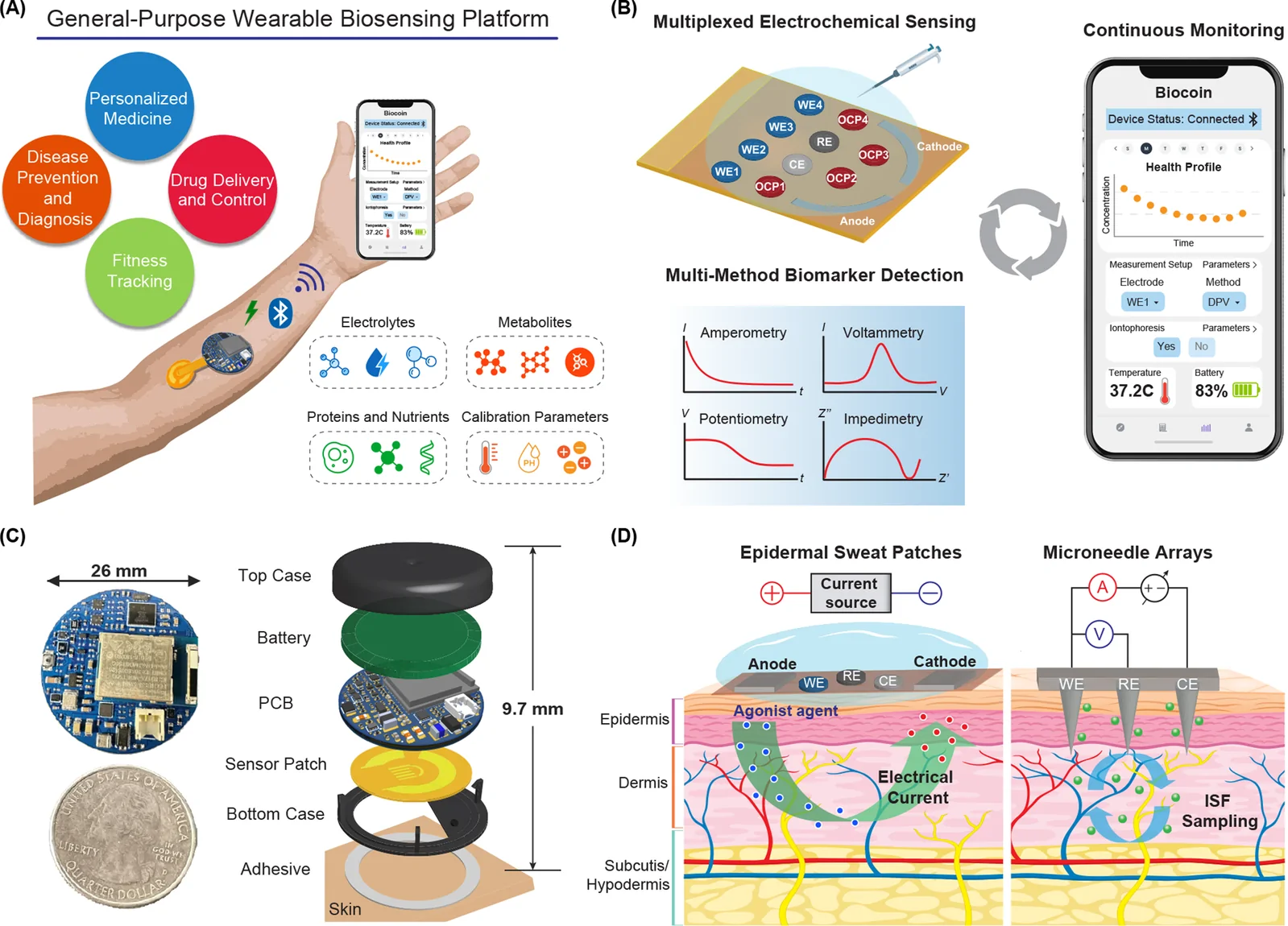
Conceptual overview of the open-source wearable biosensing platform. **A** Vision of the ultra-low power wearable device wirelessly transmitting physicochemical biomarker information to a smartphone for noninvasive health monitoring applications. **B** The device enables multiplexed and multi-method electrochemical sensing with measurements continuously controlled and monitored through a mobile application, providing personalized insights. **C** Photograph of the miniaturized wearable device adjacent to a US quarter-dollar coin and exploded view of the wearable’s assembled subcomponents. **D** A cross-sectional skin illustration highlighting representative epidermal sensing interfaces (e.g., iontophoretic sweat patches and microneedle arrays) compatible with the platform architecture for biomolecular analysis of sweat and ISF.

To address these shortcomings, this work presents Biocoin, the first open-source, open-hardware wearable biosensing platform that simultaneously meets the stringent multiplexing, power consumption, form factor, and quantitative performance requirements of modern biosensors. The fully integrated wireless electronic system was designed to autonomously and continuously execute multi-electrode, multi-method physicochemical analysis, making it ideal for biomarker panel detection across diverse health applications (Fig. 1). Compared to prior wearables, Biocoin offers exceptional multiplexing (>10 sensor inputs) and the broadest suite of sensing capabilities (10 methods) within just 530 mm^2^, rendering it the most feature-dense wearable biochemical device reported to date (Supplementary Table 1). Performance during pulsed voltammetry—techniques notoriously difficult for battery-powered devices—is particularly emphasized. Among systems with similar wireless connectivity, it also exhibits the lowest power consumption during both deep sleep and measurement modes, attributable to meticulous architectural and firmware optimizations that support months of uninterrupted sensing. Biocoin’s primary objective, however, is to serve as an enabling technology platform, prioritizing accessibility and reproducibility to unlock on-body sensor validation opportunities previously unattainable for researchers due to the siloization of existing wearable hardware. Therefore, all aspects of the system are released publicly, including the printed circuit board (PCB) design files, hardware fixtures, manufacturing documents, bill of materials (BoM), firmware, and software^50^. Fabricated deliberately using readily available off-the-shelf integrated circuits (ICs), Biocoin circumvents the proprietary barriers of custom IC solutions and overcomes the multiplexing, miniaturization, and iontophoresis limitations of both prior open-source electrochemical systems (Supplementary Table 2) and commercial wearables^51^. This commitment to open-source dissemination represents a step towards democratizing powerful healthcare tools that foster collaboration and widespread adoption, reducing the field’s barrier to entry. Ultimately, Biocoin represents a proven, modifiable framework for hardware specialists, and a turnkey “*click-to-order*” solution for non-specialists seeking to translate biosensors into clinical operation. This work reports comprehensive electrical and electrochemical benchtop validation of the platform, demonstrating its suitability for integration with emerging biosensors and adoption by the broader community.

## Results and discussion

### The Biocoin wearable biosensor platform

Comprised of a disposable epidermal sensor patch, reusable readout circuitry, and a small 110 mAh rechargeable battery stacked within a 3D-printed casing, Biocoin supports concurrent biofluid sampling, signal transduction, data processing, and wireless communication (Fig. 1A–C and Supplementary Figs. 1 and 2). By discarding the sensor patch at the end of its functional lifetime, this blended, reusable/disposable system approach aims to mitigate biofouling and signal drift challenges^52^. At the core of the Biocoin system lies a miniaturized PCB (Fig. 1C), measuring just slightly larger than a US quarter-dollar coin, that bridges accessibility with benchtop-level performance by co-integrating strategically selected commercial ICs with our custom open-source firmware stack. The modular design and reconfigurable analog sensor interface supports amperometry, potentiometry, various forms of voltammetry, impedance spectroscopy, and physical sensing techniques, enabling a broad spectrum of electrochemical sensors (Supplementary Table 3). Capable of multiplexed biomarker detection and the monitoring of common sensor accuracy confounders (skin temperature, pH, ionic strength), the device facilitates real-time sensor calibration as well as cross-analyte correlation^48,53^. A versatile electrode interface and the integration of a programmable iontophoresis current source (with overcurrent protection) render the system compatible with both ISF and sweat sensors, extending the wearable’s diagnostic utility (Fig. 1D). Data telemetry is handled via Bluetooth low-energy (BLE), allowing for visualization and analysis on a host device. The system’s embedded sleep algorithm incorporates programmable power-latency trade-offs that minimize power consumption across all modes of operation, prolonging operational lifetime and eliminating concerns about frequent battery recharging. When adhered to the skin and paired with a non-invasive microneedle array or microfluidic sweat patch, this ‘lab-on-the-skin’ concept enables continuous in situ biochemical profiling, even during everyday sedentary activities.

### Electronic device design, architecture, and integration

**Fig. 2:**
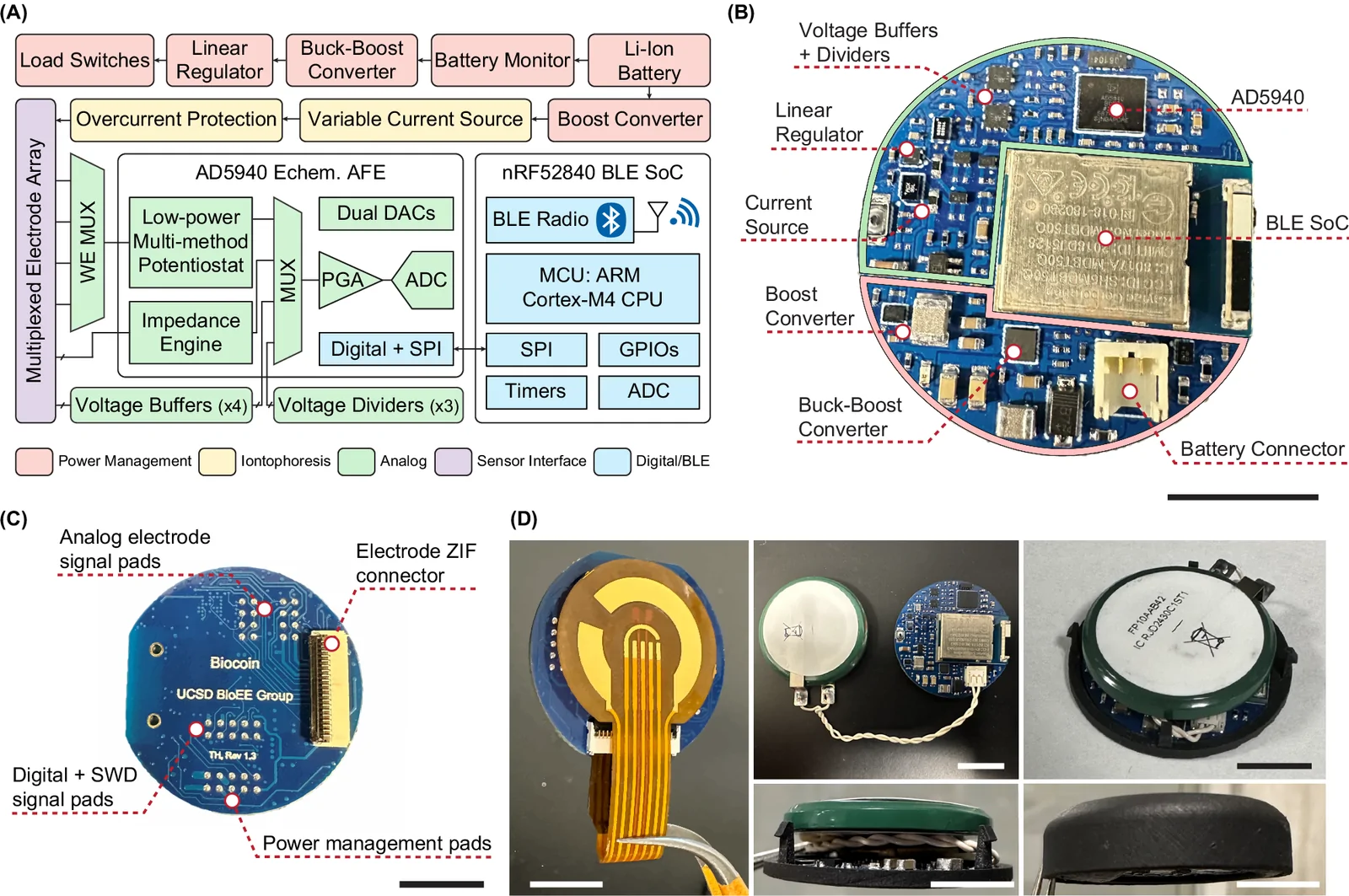
Electronic system design and integration. **A** Block diagram of the wearable system. **B** Annotated photograph of the Biocoin PCB front side showcasing all integrated circuits. **C** Photograph of the PCB back side, annotating the electrode interface and subsets of electrode, power, and digital programming signals routed to exposed metal pads that facilitate connection to the docking station. **D** Photographs of the wearable device at various stages of assembly. Reusable PCB connected to a representative disposable electrode sensor (left). Rechargeable battery plugged into the PCB through a miniaturized cable and receptacle (top middle). Side-view of the battery and PCB assembled into a 3D-printed bottom case piece (bottom middle). Top view of the battery, PCB, and bottom casing (top right). Fully assembled device encapsulated in a 3D-printed top case (bottom right). Scale bars are 1 cm.

The electronic components (Fig. 2A) can be functionally grouped as follows: (1) precision analog circuits responsible for high-fidelity signal acquisition and biomarker quantification, (2) a high-compliance voltage iontophoresis current source with real-time current monitoring and overcurrent protection for exercise-free sweat stimulation, (3) a low-power microcontroller (MCU) and BLE module for measurement control, data processing, and wireless communication, and (4) a high-efficiency network of voltage regulators that provide flexible battery power management (explained in more detail later). The analog blocks can be further divided into subsystems comprising a low-noise potentiostat analog front-end (AFE), a 4:1 working electrode (WE) multiplexer (MUX), four voltage buffers, and three voltage-divider-based resistive sensors. Together, they enable multiplexed, multi-method electrochemical analysis, along with the measurement of physical parameters. The wearable platform is orchestrated by the Nordic nRF52840 system-on-chip (SoC) integrating an ARM Cortex-M4 MCU and low-power BLE radio, which manages all measurement control, housekeeping tasks (e.g., analog block activation, AFE register configuration, sample acquisition, processing, and protection checks against iontophoresis overcurrent and undervoltage battery scenarios), and data transfer. Unlike many other low-power BLE SoCs (Supplementary Table 4), the nRF52840 is also supported by Arduino-compatible development frameworks (the Adafruit nRF52 board support package), enabling firmware development using familiar Arduino-based toolchains and APIs. This compatibility simplifies firmware development and lowers the barrier to entry for researchers adopting the open-source platform. Credited to the selection of minimal-footprint, power-efficient ICs, the compact PCB layout reflects considerable measures taken to balance miniaturization with battery lifetime and electrical isolation (Fig. 2B). After determining the optimal battery (Supplementary Note 1), the PCB was approximately matched in diameter to avoid further increasing the size. Biocoin interfaces with a wearable electrode patch through a high-density zero-insertion-force (ZIF) connector on the underside of the PCB, routing the epidermally transduced signals to their readout electronics (Fig. 2C). This approach provides strong mechanical support with reliable contact and is compatible with both sweat patches and microneedle arrays. Figure 2D showcases the system integration at varying levels of vertical assembly, including a prototype electrode patch designed to illustrate the sensor connection. Despite the compact stacked architecture, including placement of the battery above the BLE module, only a minor reduction in received signal strength indicator (RSSI) (≤ ~3 dB) was observed under on-body conditions, with no observable increase in packet loss or degradation in link reliability (Supplementary Fig. 3). Further design information about the electrode patch, overall mechanical assembly, and an auxiliary “docking station” PCB (developed for battery recharging, firmware programming, and characterization) is provided in “Methods” and Supplementary Note 2.

### Electrochemical and analog subsystems validation

**Fig. 3:**
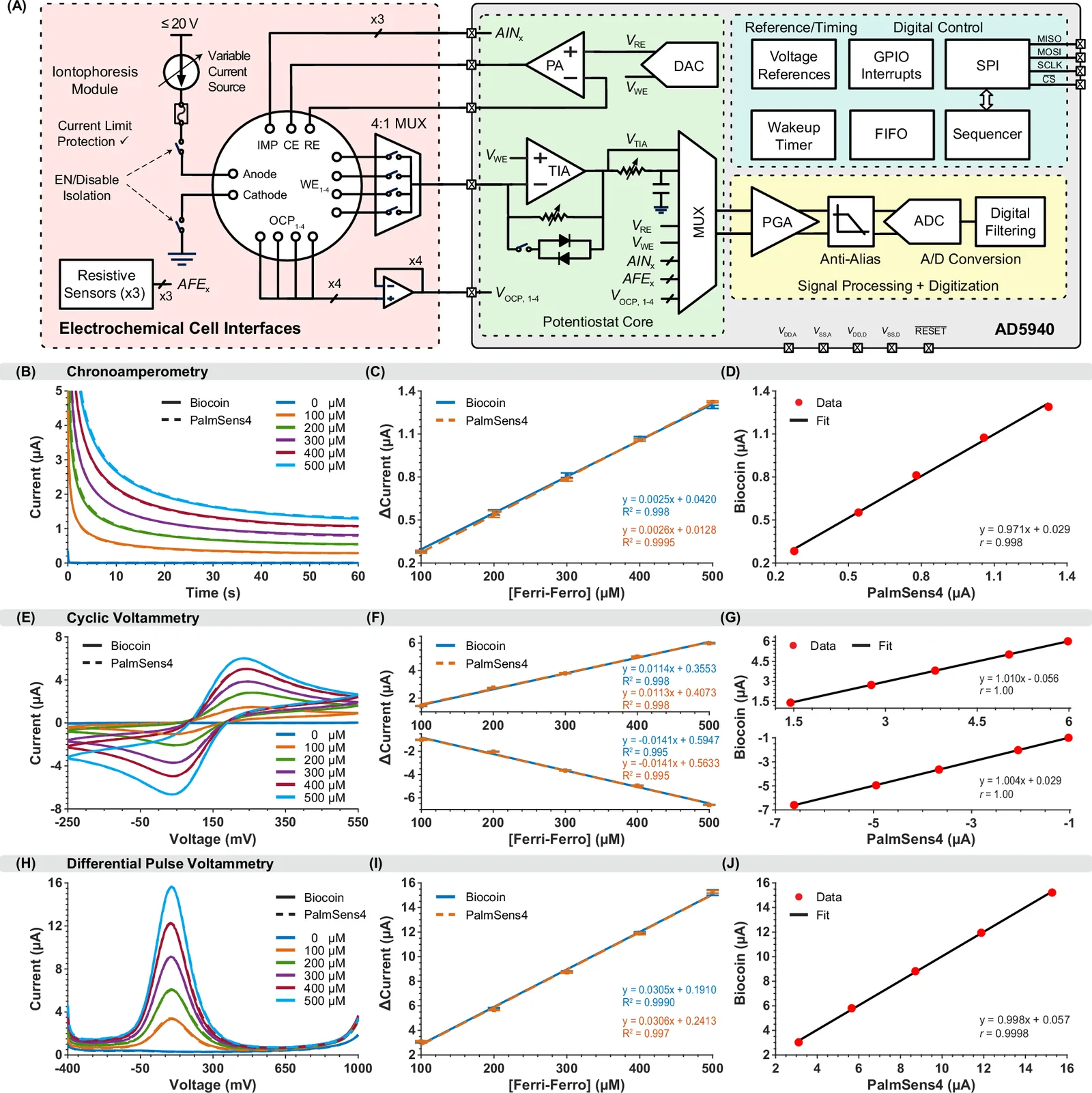
Biocoin front-end schematic and performance benchmarking vs. a commercial PalmSens4 potentiostat. **A** Schematic of all analog circuits that interface with the wearable sensor array. MISO, master in slave out; MOSI, master out slave in; SCLK, serial clock; CS, chip select. Chronoamperometry measurements, including **B** overlaid chronoamperograms, **C** corresponding calibration curves, and **D** Deming regression fit comparing both devices. Cyclic voltammetry measurements, including **E** overlaid voltammograms, **F** corresponding calibration curves overlaid with the oxidation peak responses (top) and reduction peak responses (bottom), and **G** Deming regression fits comparing both devices with the oxidation peak comparison (top) and reduction peak comparison (bottom). Differential pulse voltammetry measurements consisting of **H** overlaid voltammograms, **I** corresponding calibration curves overlaid, and **J** Deming regression fit comparing both devices. All data were recorded using potassium ferri-/ferro-cyanide (Ferri-Ferro) solutions (100–500 µM K_3_[Fe(CN)_6_]/K_4_[Fe(CN)_6_] in 1× PBS) with equivalent method parameters for the two devices. All calibration and regression data presented are mean values. Error bars represent one s.d. from *n* = 3 readings.

A detailed schematic of the wearable’s analog subsystems is depicted in Fig. 3A, highlighting all sensor interface hardware. Biosensor signal transduction and readout are handled by the Analog Devices AD5940, a highly reconfigurable AFE that integrates all the features required for electrochemical instrumentation, substantially reducing PCB area and power consumption compared to prior art built around discrete amplifiers and data converters^9,16,24,27,29^. Compared with other dedicated electrochemical front-end chipsets (Supplementary Table 5), the AD5940 provides high-resolution, wide-dynamic-range current measurement capabilities while integrating critical backend digital sequencing logic and memory, enabling autonomous operation. An external low-leakage 4:1 MUX was added in series with the WE node to allow for crosstalk-free time-division multiplexing of up to four different amperometric/voltammetric sensors, expanding the AFE’s total electrode support beyond what is natively possible. Measured data, along with AFE settings, are stored in internal registers and read back or programmed by the MCU through the serial peripheral interface (SPI). Detailed descriptions of the AFE’s signal chain, reconfigurable parameters, and digital backend functionality are outlined in the “Methods”.

To assess the performance, Biocoin was benchmarked against a high-performance commercial potentiostat (PalmSens4, PalmSens) through a comparative study using three representative electrochemical methods: chronoamperometry (CA), cyclic voltammetry (CV), and differential pulse voltammetry (DPV) (Fig. 3B–J). From the overlaid chronoamperogram and voltammogram datasets, the two devices produce nearly identical results over current ranges typical of wearable sensors, thereby validating the AFE signal path configurations and the implementation of the different electrochemical methods in the custom firmware. Calibration curves demonstrated excellent agreement and linearity, with Deming regressions yielding slopes and correlation coefficients close to unity, indicating near-perfect equivalence between the miniaturized device and a benchtop potentiostat. Moreover, to reinforce the platform’s compatibility for practical biosensing applications, Biocoin was interfaced with a commercial enzyme-based glucose test strip and used to perform amperometric measurements in artificial ISF, demonstrating a linear response across physiologically relevant concentrations (Supplementary Fig. 4).

Biocoin’s multimodal sensing capabilities were further expanded through three enhancements that reuse the AFE’s backend conditioning and analog-to-digital converter (ADC) blocks, avoiding significant PCB overhead. First, all remaining auxiliary analog inputs were pinned out for impedance (IMP) sensing (Fig. 3A). Biocoin supports three impedance analysis modalities: 2- and 4-wire configurations for quantifying parameters such as ionic strength, sweat rate, bioimpedance (Bio-Z), or galvanic skin response (GSR); and electrochemical impedance spectroscopy (EIS), useful for electrochemical cell characterization. These techniques leverage the AFE’s internal waveform generator, high-bandwidth excitation loop, and discrete Fourier transform (DFT) engine^54^, demonstrating precise impedance measurements (<±0.5% error) with strong linearity (*R*^2^ = 1.000) across an 80 dB dynamic range sufficient for biochemical applications (Supplementary Fig. 5).

**Fig. 4:**
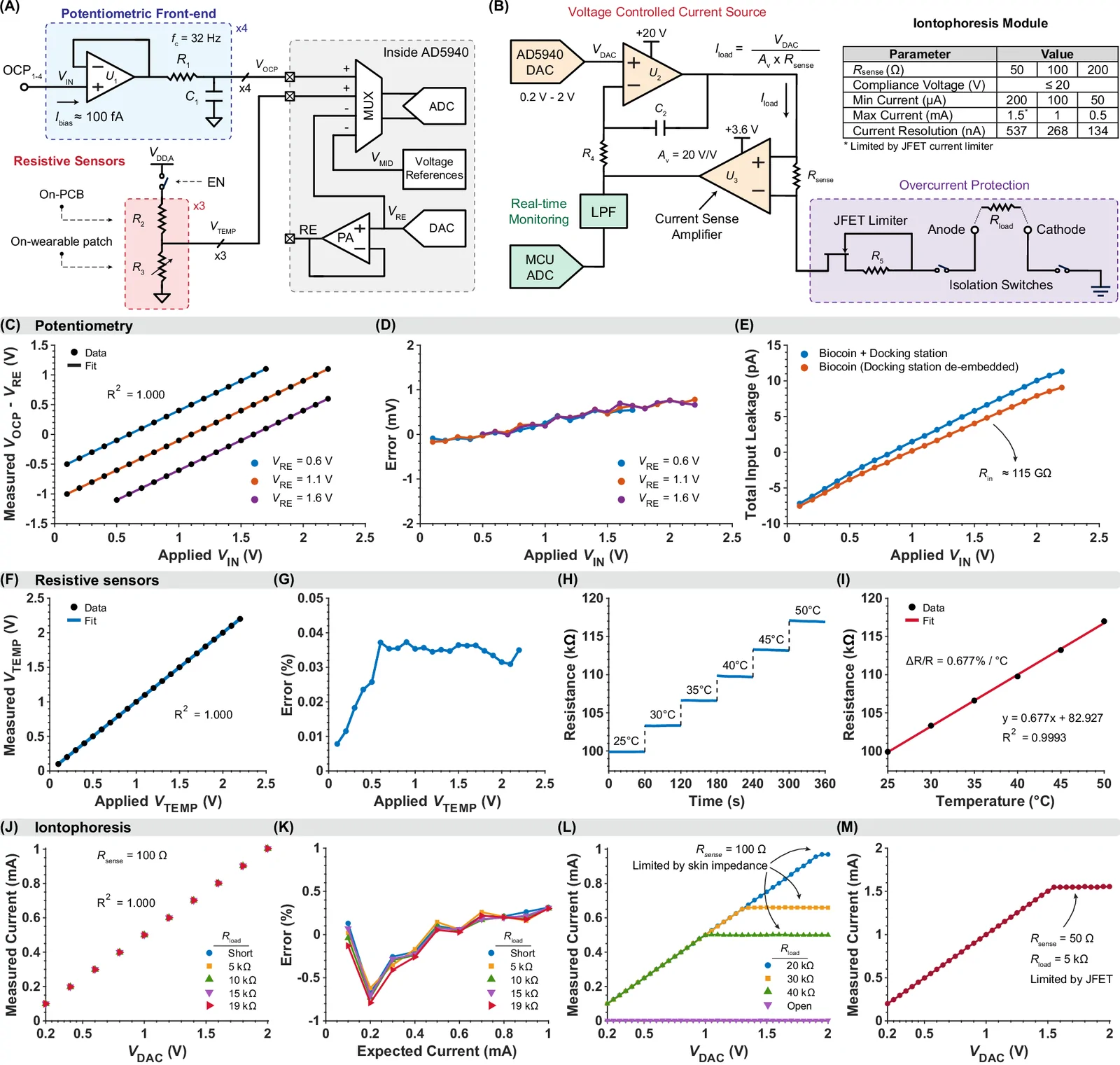
Design and characterization of the wearable’s potentiometric front-end, resistive sensors, and iontophoresis module. **A** Schematics of the OCP voltage buffers and resistive voltage dividers used for potentiometric and physical sensing, respectively. **B** Schematic of the iontophoresis current source, including the real-time overcurrent monitoring and protection circuitry. Performance summary table for different *R*_sense_. Potentiometric front-end measurements consisting of **C** transfer curve vs. applied benchtop input potentials for different RE voltages (*V*_RE_), **D** corresponding error between the measured vs. applied voltages with the error expressed in mV (not %) to avoid divide-by-zero conditions, and **E** input leakage current for the OCP channels and their equivalent *R*_in_, including all leakage sources (PCB and buffers). Resistive sensor measurements, including **F** transfer curve vs. applied benchtop input potentials (*V*_TEMP_), **G** corresponding error (%) between the measured vs. applied voltages, **H** response of a linear thermistor-based temperature sensor (*R*_3_) in the physiological temperature range, and **I** corresponding thermistor calibration curve. Iontophoresis module characterization, including **J** transfer curve *I*_load_ vs. *V*_DAC_ under different skin impedance (*R*_load_) conditions that do not exceed compliance (*I*_load_×*R*_load_ ≤ 20 V). *I*_load_ measured via the current sense amplifier output by the MCU’s ADC. *R*_sense_ = 100 Ω and *V*_DAC_ = 0.2–2.0 V, **K** corresponding error (%) between the measured vs. expected load current, **L** circuit behavior under larger skin impedance conditions that do exceed the compliance, and **M** validation of the JFET current limiter, where *R*_sense_ = 50 Ω and *R*_load_ = 5 kΩ.

As depicted more thoroughly in Fig. 4A, the second enhancement involves the inclusion of two dual-channel (4×) unity-gain buffers, featuring ultra-high input impedance, which enables the open-circuit potentiometry (OCP) electrochemical method for up to four electrodes. OCP characterization (Fig. 4C, D) demonstrated strong linearity with <±1 mV error across a 0.1–2.2 V range (set by the AFE’s ADC) (Supplementary Note 3). Considering a typical pH sensor (the most ubiquitous OCP application) with a near-Nernstian sensitivity of ~59 mV/pH at room temperature, this 2.1 V differential range easily covers the full 0–14 pH scale. Identical performance is achieved across reference electrode (RE) common-mode voltages (*V*_RE_), allowing a given sensor to be situated within the circuit’s input range. Coupled with a measured input resistance of 115 GΩ (Fig. 4E), the OCP channels provide immunity to common potentiometric sensor non-idealities, such as electrode offset voltages and large source impedances^55,56^.

Finally, three resistive sensors, implemented as voltage dividers (Fig. 4A), support measurement of physical parameters, such as skin temperature, stress, or strain. Like OCP, these channels showcase a 0.1–2.2 V input range with *R*^2^ = 1.000 linearity and <0.04% transfer curve error (Fig. 4F, G). Demonstrative temperature sensing with a linear thermistor (*R*_3_) exhibited high sensitivity (Δ*R*/*R* of 0.677%/°C) and exceptional linearity (*R*^2^ = 0.9993) across the physiological range (Fig. 4H, I), validating the AFE’s ability to monitor these changes. Supplementary Note 4 provides additional design considerations and circuit-level operation details for the potentiometric, resistive, and impedimetric blocks.

### Programmable iontophoresis module with overcurrent protection

In the absence of vigorous exercise, limited sweat production often necessitates the use of iontophoresis in wearable biosensing systems to capture dynamic biomarker profiles with high temporal resolution. To that end, a high-compliance voltage current source was added to generate precise stimulation currents that enable on-demand, localized iontophoretic sweat induction (Fig. 3A). This design also supports current generation for applications involving reverse iontophoresis, such as drug delivery or ISF extraction. However, implementing an iontophoresis module within a compact battery-powered device is challenging due to constraints in programmability, safety, power, and PCB area. The critical design considerations and circuit operation details, alongside a comparative discussion of the proposed architecture with prior art, are provided in Supplementary Note 5. The full iontophoresis module occupies just a small fraction of the total PCB area (<7%) and consists of a programmable voltage-controlled current source with overcurrent protection, skin electrodes (anode and cathode) for closed-circuit current delivery, and two switches that provide isolation from the electrochemical cell when the module is inactive (Fig. 4B). The architecture employs two anti-parallel amplifiers and a sense resistor (*U*_2_, *U*_3_, and *R*_sense_) in negative feedback to generate the stimulation current, controlled by the AFE’s 12-bit digital-to-analog converter (DAC) output (*V*_DAC_) that provides programmability while reusing existing hardware. A 20 V compliance voltage is achieved through the on-board boost converter, providing resilience against large skin impedance. Safety is ensured by a junction field-effect transistor (JFET) current limiter (~1.5 mA maximum), electrode isolation switches, and real-time monitoring of the delivered current^57^. This overcurrent monitoring is conveniently achieved without additional active components by directly measuring the current-sense amplifier’s output voltage with the MCU’s internal ADC. Using an applied *V*_DAC_ from 0.2 to 2.0 V, the default output current range spans 0.1–1 mA with ~268 nA resolution, but can be easily shifted with different *R*_sense_ values, making this architecture compatible with recent iontophoresis demonstrations using carbachol and 50–100 µA of stimulation current^29,31,48^. Electrical performance characterization of the module confirmed <1% error in current accuracy across physiological skin impedances, with the desired clamping behavior in over-compliance conditions (*I*_load_×*R*_load_ ≥ 20 V) (Fig. 4J–L). In overcurrent scenarios, the JFET clamps the current, validating reliable protection (Fig. 4M). This flexibility and tolerance to load impedance variation ultimately enable fine-tuning of stimulation current values commonly used to optimize sweat secretion rate across different sweat induction and sampling module properties (e.g., various electrode geometries, agonist agents, gel adhesives, and microfluidics). As explained later, the iontophoresis circuitry consumes low quiescent power, supporting continuous sweat replenishment throughout the day for autonomous biomarker analysis.

### System power management

**Fig. 5:**
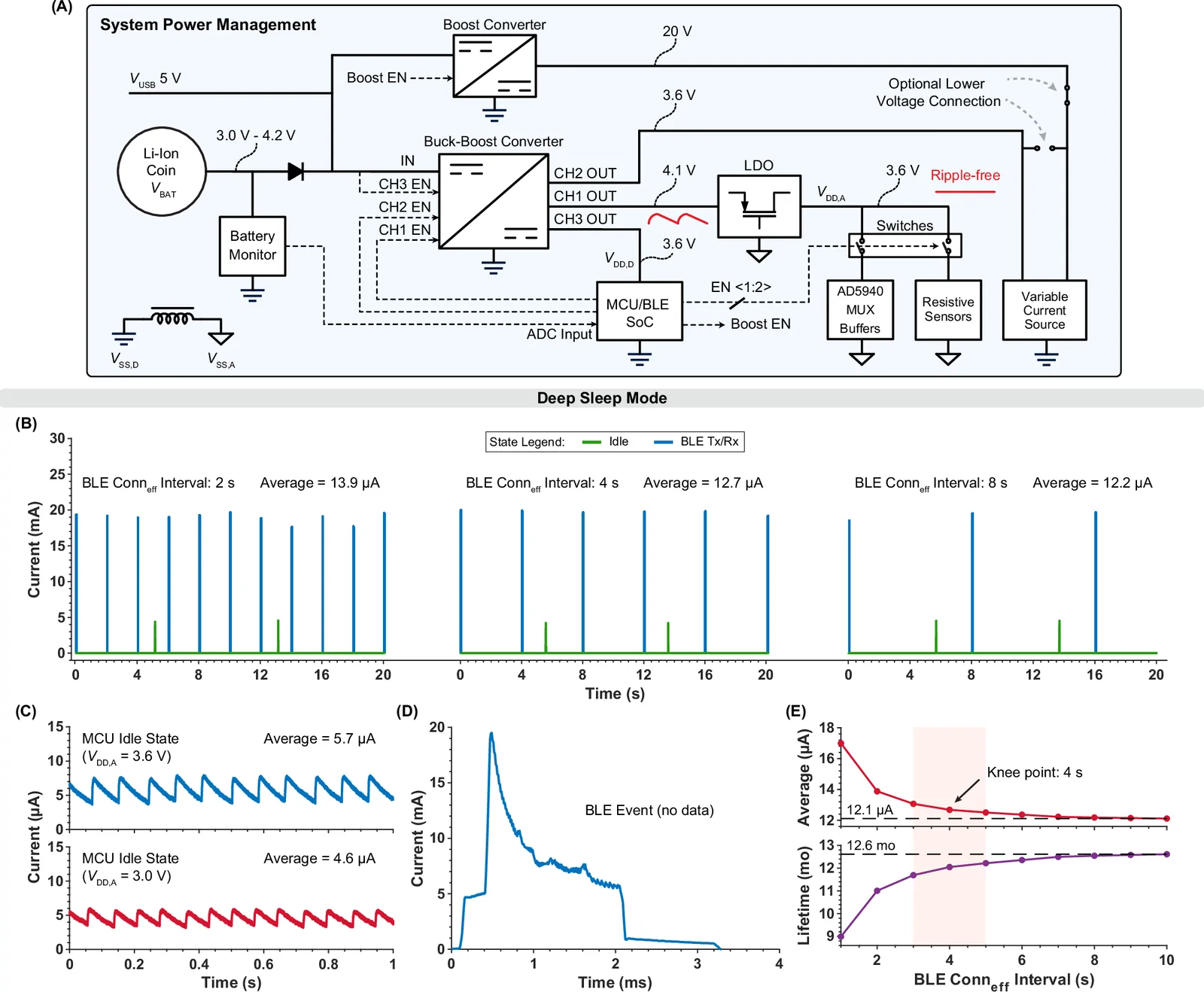
System power management and deep sleep mode characterization. **A** Detailed schematic of the system’s power management architecture. Annotated voltages represent the configuration for maximum potentiostat compliance. Characterization of the wearable device’s quiescent current consumption while operating in the deep sleep mode, including **B** transient deep sleep current profiles with 2, 4, and 8 s effective BLE connection interval, **C** zoomed-in transient current profiles of the MCU idle state when *V*_DD,A_ = 3.6 and 3.0 V, **D** zoomed-in transient current profile of a BLE connection event with no measurement data transmitted, and **E** average current consumption and estimated PCB battery lifetime in deep sleep vs. effective BLE connection interval. Shading highlights the region of optimal power-latency tradeoffs. For all plots, unless stated otherwise, *V*_DD,A_ = 3.6 V. In all cases, the voltage of *V*_DD,D_ equals that of *V*_DD,A_, and the electronic system was powered at 3.7 V (*V*_BAT_).

The system’s power management is integral to enabling long-term health monitoring. Carefully engineered for ultra-low power operation, the architecture (Fig. 5A) emphasizes block-level power gating, high-efficiency regulation, and minimal quiescent current draw. First, the Li-Ion battery voltage is periodically tracked by a low-power (~1 µA) always-on monitor (Supplementary Figs. 6 and 7), enabling dynamic reduction of AFE sampling or BLE rates in low-voltage scenarios to prolong operation. Following this, the battery is managed by two parallel switching regulator networks: a boost converter that supplies the iontophoresis current source with 20 V and a buck-boost converter that feeds the MCU and downstream analog circuits. In battery-powered transimpedance-based potentiostats, the limited supply voltage inherently constrains the allowable electrode/amplifier voltage swings, often causing amplifier saturation or mandating reduced transimpedance gain (and, consequently, reduced sensitivity), particularly during transient electrochemical measurements with large double-layer capacitances or counter electrode (CE) overpotentials. As reviewed thoroughly in Supplementary Note 6 and Supplementary Fig. 8, the adopted buck-boost converter regulation scheme overcomes this critical limitation by allowing the AFE to operate flexibly at any supply voltage (*V*_DD,A_) across its supported 2.8–3.6 V range, while maintaining system operation across the full battery range (3.0–4.2 V). Accordingly, the design is tailored for high-sensitivity electrochemical sensing and demanding pulsed voltammetry techniques.

The buck-boost converter facilitates area compactness by employing a single-inductor multiple-output (SIMO) topology with three controllable outputs, consuming nA-level quiescent current: one always-on channel (CH3) for the MCU and two duty-cycled channels (CH1 and CH2) for all analog blocks. This split-rail design also permits separation between analog and digital supplies (*V*_DD,A_/*V*_SS,A_, *V*_DD,D_/*V*_SS,D_) for improved electrical isolation without the area penalty of additional regulators. A low-dropout linear regulator (LDO) follows the buck-boost converter to filter the switching ripple, thereby preserving the analog measurement fidelity. Finally, two subsequent load switches enable the selective activation of the electrochemical or resistive circuits as needed. To demonstrate the power management’s versatility and power consumption implications, two analog supply configurations are characterized herein: (1) *V*_DD,A_ = 3.6 V, enabling maximum compliance at higher consumption, and (2) *V*_DD,A_ = 3.0 V, offering significant power savings in relaxed compliance applications. Supplementary Note 7 provides an in-depth analysis of the complete set of voltage-domain considerations and architectural justifications for this cascaded regulator approach.

### Ultra-low power wireless sensing

Co-optimized with the power management system, Biocoin’s firmware and BLE protocol design critically influence battery life, data latency, and overall user experience. An event-driven architecture prioritizes low-power sleep states, rapid wake-ups, and aggressive hardware power gating, keeping the MCU and AFE predominantly in their lowest-power states (Supplementary Fig. 9). After BLE pairing, the system automatically enters a low-power idle state until commanded to do otherwise. When prompted, the AFE autonomously acquires sensor data with minimal MCU intervention and is power-gated between ADC sampling events (Supplementary Fig. 10), supporting application-specific trade-offs between power and acquisition rate. All electrochemical and physical sensing methods, including iontophoresis, follow this framework (Supplementary Figs. 11 and 12). Firmware was developed using a class-based framework for modularity and simplified extensibility (Supplementary Fig. 13). During BLE operation, Biocoin exposes a tailored server profile to its host (Supplementary Fig. 14), enabling wireless transfer of instructions, AFE settings, and sensor data. Custom software and a graphical interface provide full wireless configuration and logging capabilities (Supplementary Fig. 15). Collectively, these efforts allow seamless transitions between advertising, initialization, sleeping, and measurement operations, with transient current consumption profiles that vary drastically according to the device’s activity level (Supplementary Fig. 16a).

Using this framework, we define three operating modes for Biocoin, each comprising various states and tasks judiciously refined to eliminate superfluous power consumption (Supplementary Note 8 and Supplementary Fig. 17): (1) deep sleep, where the BLE link is maintained but the MCU idles and the AFE powers off, (2) active mode, encompassing any measurement method with data operations (e.g., sampling, processing, and transmission), and (3) current-monitored iontophoresis. Beginning with deep sleep, the MCU cycles between its idle state and BLE radio events that preserve the wireless connection. Leveraging the BLE slave latency parameter, these events align with the effective BLE connection interval (BLE conn_eff_ interval), yielding current profiles marked by seconds-long ultra-low power sleep (5.7 µA, which further drops to 4.6 µA with *V*_DD,A_ = 3.0 V) interrupted by brief (ms-scale) radio spikes near 20 mA (Fig. 5B–E). Despite these transient peaks, the profile averages remain close to the sleep current floor, corresponding to estimated battery lifetimes approaching 1 year.

**Fig. 6:**
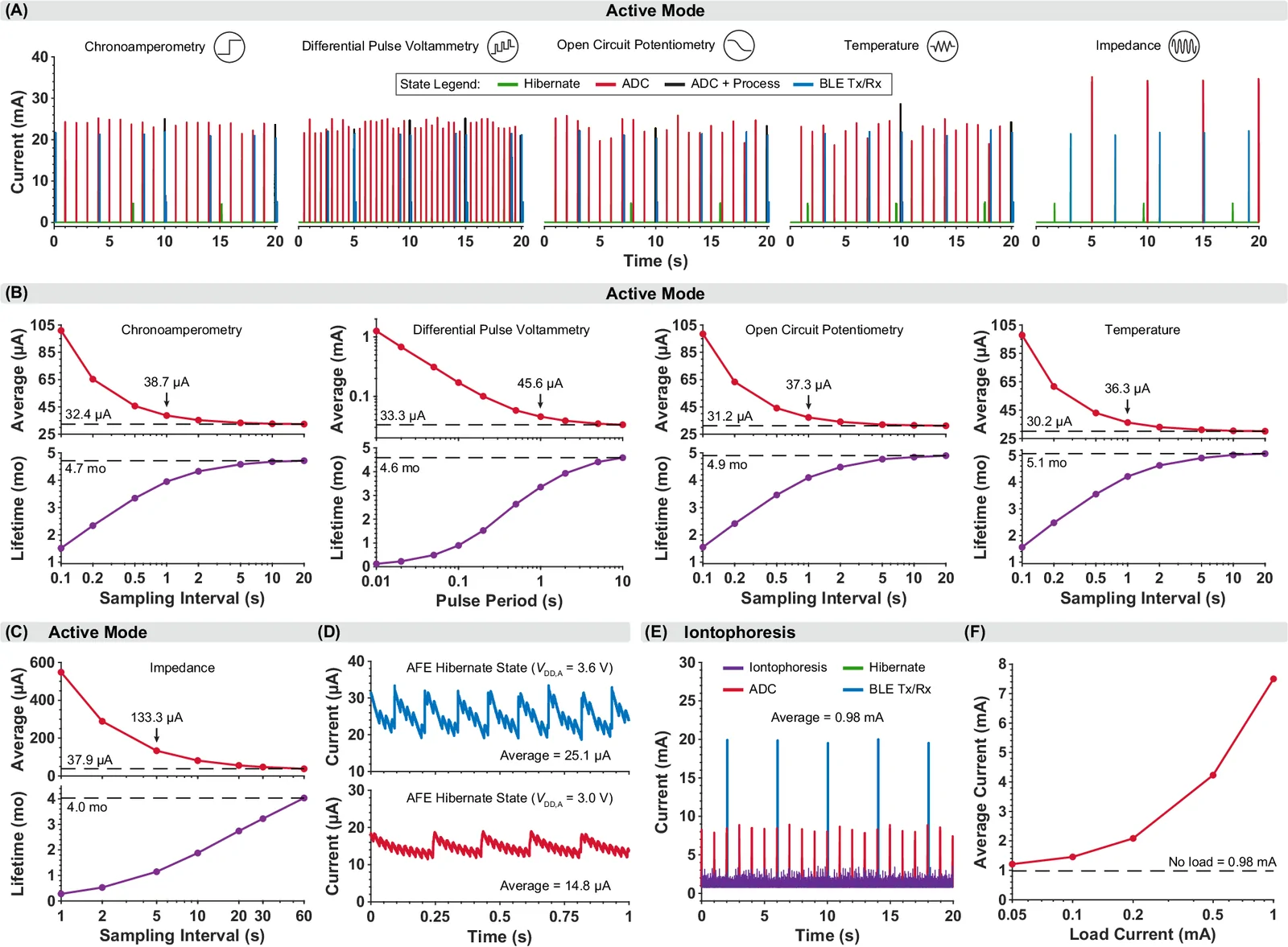
Characterization of the wearable system’s current consumption during active and iontophoresis modes. Characterization of the wearable device’s quiescent current consumption during various active mode operations, including **A** transient current profiles while executing different electrochemical or physical measurement techniques, and average quiescent current consumption and estimated PCB battery lifetime across varying sampling intervals (or pulse periods) for **B** chronoamperometry, differential pulse voltammetry, open circuit potentiometry, temperature, and **C** impedance measurement methods. Values annotated with arrows represent the average current consumption of the corresponding transient profiles from A. **D** Zoomed-in transient current profiles of the AFE hibernate state when *V*_DD,A_ = 3.6 and 3.0 V. Characterization of the wearable device’s current consumption during iontophoresis with a 20 V compliance, including **E** transient quiescent current profile during current-monitored iontophoresis with no load, and **F** average current consumption during iontophoresis while delivering various load currents. For all plots, unless stated otherwise, *V*_DD,A_ = 3.6 V. In all cases, the voltage of *V*_DD,D_ equals that of *V*_DD,A_, and the electronic system was powered at 3.7 V (*V*_BAT_). A 100 ms BLE connection interval and a 4 s effective BLE connection interval were used for all cases.

Active-mode power consumption was evaluated across parameters governing data collection and BLE transmission (Supplementary Fig. 18 and “Methods”). Each parameter—most notably the processing interval, defining how often the MCU reads and queues AFE data for transmission—was optimized to balance power and communication latency without compromising connectivity range or user experience (Supplementary Fig. 19). Using these optimizations, a thorough analysis was performed on five prominent techniques in electrochemical biosensing (CA, DPV, OCP, temperature, and impedance) with their corresponding current profiles highlighted in Fig. 6A. The average currents and associated battery lifetimes of each method were characterized across realistic sampling (or pulse period) intervals until asymptotic behavior was observed, indicating when the power penalty of infrequent ADC sampling becomes negligible (Fig. 6B, C). The hibernate sleep current floor in active mode increases relative to the MCU’s idle state during deep sleep due to the LDO and potentiostat blocks remaining on throughout the measurement, but remains exceptionally low (25.1 µA), dropping to just 14.8 µA when *V*_DD,A_ = 3.0 V (Fig. 6D). Estimates for other related methods (*e.g*. square wave voltammetry (SWV) and EIS) can be inferred from these results under equivalent parameters. During iontophoresis, the system draws a quiescent current of 0.98 mA with a 1 s current monitoring interval and 20 V compliance (Fig. 6E), which increases to several mA as the stimulation current approaches 1 mA (Fig. 6F). Because iontophoretic sweat induction typically occurs for 5–15 min intervals a few times per day, this architecture therefore minimizes its impact on total lifetime. All data presented in Fig. 6 assume a buffered transmission strategy enabled by the processing interval and the AFE’s FIFO, in which multiple samples are batched prior to BLE communication. Alternative acquisition modes, including real-time streaming and other data handling strategies, introduce different tradeoffs between latency, sampling rate, and power consumption. The system’s upper limits on sampling rate, BLE throughput, and associated worst-case power consumptions under high-frequency operation are further detailed in Supplementary Note 9.

This demonstrated power-latency reconfigurability permits customization for either high-temporal-resolution biomarker monitoring or extended operational lifetime, while also supporting dynamic sampling adjustment (e.g., favoring higher resolution when the battery is fully charged vs. energy conservation as it approaches depletion) depending on the application or clinical priorities. Taking CA as an example—the method-of-choice in commercial continuous glucose monitors (CGMs)—Biocoin consumes just 38.7 µA with a 1 s sampling interval, enabling uninterrupted sampling and BLE operation for ~3.9 months. Across nearly all sensing methods and sampling conditions evaluated, the system sustains weeks-to-months of battery lifetime spanning more than two orders of magnitude in acquisition intervals. Accordingly, Biocoin shifts the primary wearable biosensor lifetime bottleneck from the electronics to sensor chemistry, allowing future advances in sensor stability and biofouling mitigation to directly translate into longer operational lifetimes. Supplementary Table 6 summarizes current consumption and lifetimes across noteworthy conditions. These scenarios were also measured using the 3.0 V *V*_DD,A_ configuration, showcasing improvements in battery lifetime ranging from 11% to 96% across the methods compared to 3.6 V, a testament to the carefully architected power management. To the best of our knowledge, the tabulated current consumptions for deep sleep and active mode at both analog supply voltages are the lowest reported for prior-art wearable biosensors operating with continuous sampling and BLE connectivity (Supplementary Table 1).

Biocoin establishes the first fully open-source, open-hardware wearable biosensing platform designed to support real-time, continuous biochemical and physical sensing on the body, addressing a long-standing barrier that has limited electrochemical wearables to in vitro or single-sensor demonstrations. The 530 mm^2^ device delivers benchtop-level accuracy (*r* ≥ 0.998) across multiple electrochemical sensing modalities, emphasizing pulsed voltammetry techniques that are challenging in battery-powered systems. Its multiplexed support (4× WE, 4× OCP, 3× resistive inputs), autonomous sensing capabilities (10 methods), and integrated iontophoresis circuitry (supporting sweat and ISF sampling or drug delivery) enable emerging biosensors to be deployed without hardware redesign. Through co-optimized system architecture and event-driven firmware, Biocoin achieves µA-level power consumption in both deep sleep and active modes, supporting months of wireless operation from a small battery, thereby eliminating recharge burdens and addressing longevity bottlenecks commonly encountered in wearable biosensing systems. By releasing all design files, firmware, and software openly, Biocoin establishes a reproducible, extensible foundation for cross-disciplinary research and enables researchers to integrate their own sensing chemistries and pursue on-body validation studies for biosensors that previously remained confined to the laboratory. While this work demonstrates thorough electrical and electrochemical platform validation, future efforts will focus on integrating next-generation sensors to reveal nuanced physicochemical health insights, alongside their validation in longitudinal clinical studies that further advance personalized and preventive healthcare. Looking forward, we anticipate Biocoin will accelerate innovation in wearable molecular sensors and serve as a widely adoptable platform that transitions continuous biochemical monitoring from the lab to life.

## Methods

### Reagents and materials

Phosphate-buffered saline (PBS, P5493) was purchased from Sigma-Aldrich. Potassium ferricyanide (P1286) and potassium ferrocyanide (P1296) were obtained from Spectrum. Screen-printed carbon electrodes (SPCEs) with a 4 mm-diameter carbon WE, carbon CE, and silver RE were purchased from Metrohm DropSens (SPE-C110).

### Fabrication of the electronic system

The wearable device was designed using commercial ICs assembled onto a coin-shaped, 6-layer FR4 PCB (530 mm^2^, radius 13 mm, height 0.8 mm) with an electroless nickel immersion gold (ENIG) surface finish (layer thicknesses: Ni 120 µ" and Au 2 µ"). The PCB was fabricated by PCB Minions, and all electronic components were sourced from DigiKey Electronics or Mouser Electronics. Component assembly was performed by Quality Systems Integrated Corporation. Schematic design and layout were performed using Altium Designer (v23.0.1). Biocoin is recharged, programmed, and electrically/electrochemically tested via an auxiliary “docking station” PCB. A detailed description of the critical components utilized on Biocoin, along with component and fabrication details of the docking station, is discussed in Supplementary Note 2. Complete schematics and BoMs for Biocoin and the docking station are provided in Supplementary Figs. 20–26 and Supplementary Tables 7–9. The BoMs contain a cost analysis of the unit price per component when purchased at small (10×) and larger (100×) quantities for both PCBs, while Supplementary Table 10 summarizes the cost breakdown for all aspects of the system (e.g., components, PCB fabrication, assembly, and 3D-printed fixture ordering) and compares the total to other commercially available potentiostat platforms. All hardware design, fabrication, and assembly files (including Altium project schematics, board layouts, gerber, drill, BoM, and pick-and-place files) are available in the GitHub repository: https://github.com/ProfDrewHall/Biocoin.

### 3D-printed mechanical fixture design

The Biocoin wearable casing and docking station connection fixtures were designed using SOLIDWORKS (v2022) and printed on a Formlabs 3B resin printer via a print preparation software (PreForm, v3.33.1). The wearable pieces were printed using a biocompatible BioMed resin (RS-F2-BMBL-01, Formlabs), while the docking station pieces were made from either black or gray resin (RS-F2-GPBK-04 or RS-F2-GPGR-04, Formlabs). Printed parts were washed in an isopropyl alcohol (IPA) bath (FH-WA-01, Formlabs) for 20 min and cured in a temperature-controlled station (FH-CU-01, Formlabs) at 60 ^°^C for 60 min. Additional information about the 3D-printed fixtures and system assembly are detailed in Supplementary Note 2 and Supplementary Figs. 1 and 2. All fixture design files are available via the GitHub repository.

### AFE signal chain and electrochemical operation

The AD5940 core integrates a transimpedance amplifier (TIA), potentiostat amplifier (PA), and dual output DAC within a three-electrode potentiostat for electrochemical measurement, control, and readout (Fig. 3A). The DAC outputs (*V*_WE_ and *V*_RE_) generate the bias potentials for the WE and RE, respectively, with 12-bit resolution in *V*_RE_ (LSB = 537.2 µV) and 6-bit resolution in *V*_WE_ (LSB = 34.38 mV), both separately configurable from 0.2 to 2.4 V. These ranges enable a total dynamic scan range (Δ*V*) up to 2.2 V, supporting the quantification of analytes with large oxidation/reduction potentials. Due to the improved resolution of *V*_RE_, time-varying electrochemical methods (DPV, CV, etc.) are implemented by programming the RE potential, while keeping the WE constant. When interfaced with an electrochemical cell, the TIA and PA form two negative feedback control loops that apply these potentials to the WE and RE, respectively. The PA drives the CE to form a closed circuit and works with the TIA to source/sink the sensor current, which is then converted into a voltage at the TIA output for further conditioning and digitization. The TIA gain is programmable from 0.2 to 512 kΩ across 26 settings, accommodating a wide dynamic range of sensor currents, and an external 33 nF feedback capacitor was added to stabilize the TIA against large electrode double-layer capacitances (up to ~1 µF). An optional *RC* low-pass filter (LPF) is also available following the TIA for additional analog filtering.

For amperometric/voltammetric measurements, the TIA output enters a low-leakage MUX/switch matrix, alongside *V*_WE_, allowing the differential signal (*V*_TIA_ – *V*_WE_) to be conditioned by a programmable gain amplifier (PGA, five gain settings: 1, 1.5, 2, 4, and 9) and second-order anti-aliasing filter (AAF, three cutoff frequencies: 50, 100, and 250 kHz) before digitization by a 16-bit, 800 kSps successive approximation register (SAR) ADC. Note that the internal AFE MUX allows either the current measurement channel or external voltage inputs (e.g., OCP, resistive sensors) to reach the downstream ADC, depending on the application. A series chain of digital filters—including sinc3 and sinc2 decimation (low-pass) filters and a 60/50 Hz mains filter—follows the ADC to further reduce high-frequency noise and improve the output signal-to-noise ratio (SNR), at the expense of higher power consumption.

### Electrochemical measurements

Biocoin supports eight electrochemical methods: CA, CV, DPV, SWV, linear sweep voltammetry (LSV), normal pulse voltammetry (NPV), OCP, and EIS (Supplementary Table 3). Electrochemical performance was characterized through amperometric and voltammetric experiments involving redox mediator solutions containing equal parts potassium ferri-/ferro-cyanide (K_3_[Fe(CN)_6_]/K_4_[Fe(CN)_6_]) diluted in 1× PBS from 100 to 500 µM. Measurements were conducted by drop-casting 100 µL of each sample concentration onto commercial SPCEs, with Biocoin and the SPCEs installed in the docking station. The following parameters were used: CA at 0.5 V for 60 s with 0.1 s sampling; CV with -0.25 V Estart, 0.55 V Evertex1, -0.26 V Evertex2, 5 mV Estep, and 20 mV/s scan rate; and DPV with a −0.4 –1.0 V scan range, 5 mV Estep, 100 mV Epulse, 50 ms pulse, and 20 mV/s scan rate. Measurements were collected three times consecutively for each method, and a separate SPCE was used for each method. Raw data were baseline-corrected by point-wise subtraction of the PBS-only (0 µM) curves. Calibration curves were derived from the final sample points or oxidation/reduction peak maxima, and sensitivity and linearity were evaluated using linear regression and coefficients of determination (*R*^2^).

### Performance benchmarking with a commercial potentiostat

To validate the implementation of the different electrochemical methods in custom firmware, the electrochemical responses of Biocoin were compared with those of a high-performance, commercially available potentiostat (PalmSens4, PalmSens). The electrochemical measurements previously described were repeated using a PalmSens4. The same SPCEs used for Biocoin were reused here for each method to remove the effect of electrode mismatch from the measurements, isolating only differences in electronic performance and firmware. Identical method parameters were used for fair comparison. Qualitative comparison was performed by overlaying the raw chronoamperograms, voltammograms, and calibration curves from both devices. Quantitative comparison was accomplished by fitting a Deming regression to the mean values reported in the calibration curves of the two potentiostats (since both data sets contain measurement uncertainty) and computing the Pearson correlation coefficient (*r*).

### Electrical performance evaluation and power consumption characterization

Electrical validation of Biocoin’s circuitry was performed using high-performance lab instrumentation and/or the MCU and AFE’s built-in ADCs. The analog subsystems support electrochemical, impedimetric, and resistive sensing, current-monitored iontophoresis, and battery voltage monitoring, all of which were functionally verified. Linear regressions, coefficients of determination (*R*^2^), and error plots between the measured and expected results were computed to evaluate performance. The system’s power consumption during different tasks, states, and modes of operation was also characterized. Detailed descriptions of the considerations, measurement setups, and equipment used for validating each subsystem are provided in Supplementary Note 3.

### Embedded firmware development

The firmware was developed in C/C++ on a computer using the PlatformIO extension (v6.1.18) within the Visual Studio Code integrated development environment (IDE, v1.102.0), allowing firmware to be written, compiled, and uploaded to the device. The Nordic nRF52 platform (v10.8.0) was installed using the Arduino framework and the Adafruit Feather nRF52840 Express board support package (BSP), which provides a hardware abstraction layer built on top of Nordic’s native software development kit (SDK). This approach enables firmware development using standard C/C++ and Arduino-style APIs while still providing access to the nRF52840’s platform-specific features. Although the Biocoin firmware includes device-specific peripheral configuration and analog front-end control routines, this Arduino-compatible BSP and development toolchain was intentionally selected to simplify compilation, programming, and debugging compared to development directly with Nordic’s native SDK. Firmware can also be developed directly in the Arduino IDE via the Adafruit nRF52 package. Connection to the IDE used a micro universal serial bus (USB) connector plugged into the docking station with Biocoin installed. Upon first power-up, the MCU’s internal bootloader was burned using a serial wire debug (SWD) probe (J-Link Pro, SEGGER) to enable future firmware uploads. Since the nRF52840 supports native USB, all subsequent programming was performed directly via the USB interface. It also supports over-the-air (OTA) updates via BLE. Note that the electrochemical firmware was built by extending AD5940 example applications with method-specific modifications, additional sequences, and signal-chain optimizations.

### MCU event-driven firmware operation

The MCU’s firmware routine exploits an ultra-low power “idle” sleep state to minimize current consumption. Instead of remaining “always awake” and polling for instructions, it follows an event-driven approach that keeps the MCU asleep most of the time. After brief hardware initialization upon startup or reset (Supplementary Fig. 9), and once a BLE connection is established, the MCU’s central processing unit (CPU) is immediately put to sleep, reducing idle current draw from 3.5 mA to ~3 µA. It then stays asleep indefinitely, waking in response to six defined interrupts. Two timer-controlled interrupts handle simple housekeeping procedures (battery voltage monitoring and status light-emitting diode (LED) flashing), occurring once every five minutes with a combined penalty of just 0.1 µA (Supplementary Fig. 7). A third interrupt manages reception of BLE host commands during connection events, typically containing measurement instructions or start/stop requests. After deciphering the information, the MCU programs the AFE accordingly and triggers the desired method. For sampled techniques involving the AD5940, a general-purpose input/output (GPIO) pin interrupt controls data flow. Once the AFE’s first-in first-out (FIFO) data storage fills, it toggles the pin to prompt an SPI transfer of raw ADC data, which the MCU processes into appropriate units (*e.g*., potentiostat current in µA) and stores in a data queue before awaiting the next interrupt: BLE transmission. At the next BLE interval, queued data are transmitted if available; otherwise, the interrupt routine terminates early. The final interrupt occurs during current-monitored iontophoresis, where a timer prompts the MCU to check for overcurrent. If the measured current exceeds a pre-programmed safety threshold, the iontophoresis module is disabled; otherwise, normal operation continues. Critically, after any interrupt, the MCU immediately returns to idle sleep to extend battery lifetime.

### Autonomous AFE sequencing and electrochemical method implementation

The device firmware synergistically leverages four AD5940 digital blocks— sequencer, wakeup timers, FIFO, and GPIO interrupts—to execute electrochemical methods autonomously. Every method is decomposed into periodic “sequences” of DAC voltage updates and ADC sampling events (or, in some methods, only ADC sampling). Because the method parameters (scan ranges, step sizes, sampling intervals, test durations) are known in advance, the MCU precomputes all measurement instructions before starting. These instructions are loaded into the sequencer’s static random-access memory (SRAM) during AFE configuration. Once triggered, the sequencer autonomously drives DAC and ADC operations in real-time, allowing the MCU to remain in idle sleep for most of the experiment. This approach yields considerable power savings compared to systems that require frequent MCU wakeups to manage both DAC and ADC functions via SPI. The timing of sequence execution is controlled by the AFE’s wakeup timer, which defines the ADC sampling interval and DAC waveform transitions. From an application perspective, the sampling interval determines the temporal resolution at which dynamic biomarker profile changes are captured, with shorter intervals resulting in higher average power consumption.

Like the MCU, the AFE supports a low-power “hibernate” state to further minimize power during measurements. In hibernation, most internal AFE blocks are shut down; however, the low-power potentiostat blocks always remain on to maintain the electrochemical cell’s bias. The AFE hibernates between wakeup timer triggers and sequencer operations, such that the most power-intensive circuit blocks (i.e., the ADC and digital filters) are only active during the sampling sequence. This sequencer-based operation is depicted graphically in Supplementary Fig. 10. Collected samples are stored in the FIFO and compared against a programmable count threshold. If the number of FIFO samples exceeds the threshold, or the final sample has been collected, a GPIO interrupt prompts the MCU to read the data (which only takes a few milliseconds), as elaborated on previously (Supplementary Fig. 9). These interrupts represent the only times the MCU must wake for the AFE during a measurement routine.

The firmware makes the time between FIFO reads a user-controllable parameter, referred to as the processing interval, which is defined by the relationship: FIFO threshold = processing interval/sampling interval (Supplementary Fig. 18a, b). This parameter also inherently defines the BLE data transmission rate (*i.e*., the rate at which health charts/plots can be updated in real-time on a user interface), since data are queued immediately after processing. A fast sampling interval coupled with a slow processing interval means that biomarker data will be collected rapidly, but transmitted infrequently in larger chunks rather than sample-by-sample – a useful approach for applications that do not necessarily require live plotting or rapid clinical interpretation, but may benefit from higher-resolution historical records if health problems are identified.

The DAC update, ADC sampling, and wakeup timer events, as defined for each sensing method, are annotated in Supplementary Fig. 11. Importantly, during each sampling window, the ADC and digital filters run only long enough to produce one sample before returning to hibernation. Upon concluding any method, the AFE and LDO are shut down, and the system returns to deep sleep. All waveform and sampling definitions were matched to, and validated against, a PalmSens4 commercial potentiostat to ensure accuracy (Supplementary Fig. 12).

### BLE communication

Once paired with a central ‘host’ device, Biocoin acts as a peripheral and is configured as a custom Generic Attribute Profile (GATT) server, exposing key services and characteristics that form a repository of organized operational information (Supplementary Fig. 14). One custom Biocoin service was implemented with characteristics for device status, name, measurement control, waveform parameters, AFE settings, and data samples (voltages, currents, impedances). A Python library was also developed, acting as the central device, to manage complete wireless control of the wearable (Supplementary Fig. 15a,b). The firmware for the GATT server and the peripheral’s transmission/reception (Tx/Rx) communication protocol was developed using Bluefruit (v4.1.2). The Python (v3.13) software for the central was written using Bleak (v1.1.1). A graphical user interface (GUI) was built on top of the Python library using Streamlit (v1.37.0), allowing for live plotting of sensor data and experimental configuration (Supplementary Fig. 15c).

On power-up or a BLE disconnect, Biocoin transmits advertising packets to signal pairing availability (Supplementary Fig. 16a). Packets are first broadcast at a fast interval (30 ms) for low-latency connection (Supplementary Fig. 16b). If no central connects within 30 s, an immediate connection is assumed unlikely, and the interval dynamically increases to 200 ms to conserve power (Supplementary Fig. 16c). After pairing, communication occurs only during dedicated connection events, where devices exchange packets to maintain synchronization, even if application data is unavailable. Two BLE parameters govern the timing of these events: the connection interval (exchange frequency) and the slave latency, which defines how many consecutive intervals the peripheral can skip if no data needs to be transmitted. An effective connection interval can therefore be defined by: BLE conn_eff_ interval = BLE connection interval×(1 + slave latency) (Fig. 5B). Slave latency can thus be exploited to enable higher throughput data transfer when available, while increasing the Tx/Rx latency otherwise for reduced power consumption.

Consider the case where slave latency = 39 (the number of skippable events) and the connection interval = 100 ms (BLE conn_eff_ interval = 4 s): during active mode, collected data is queued for Tx at a rate set by the processing interval, which triggers transmission at the next integer multiple of 100 ms relative to the previous connection event (*i.e*., within 100 ms) (Supplementary Fig. 18a,c). When the processing interval exceeds the BLE conn_eff_ interval, the frequency of BLE events in between processing operations reduces to every 4 s until the next set of samples is queued (Supplementary Fig. 18b). Sensor data are transmitted using BLE notifications as floating point values (4 bytes per sample) at 1 Mbps. The maximum transfer unit (MTU) determines the amount of data that can be transmitted during one GATT operation, with 3 bytes reserved for the protocol header. A 247-byte MTU allows 244 notifiable bytes, or 61 floating-point samples. Queued data exceeding this limit is segmented across consecutive connection intervals (Supplementary Fig. 18d). A link-layer acknowledgement (LL Rx Ack.) from the central node occurs one interval after all queued data has been notified, confirming successful receipt.

### Power consumption optimizations

Trade-offs among operational parameters affecting data-collection timing and volume during sampling and transmission were explored to optimize power and battery lifetime while balancing connectivity range, latency, and user experience. Subsequently, the device current consumption versus sampling interval can be assessed in detail (Fig. 6). All parameters listed below are reconfigurable and user-accessible in the firmware/software, encouraging further application-specific optimizations.

#### Radio transmit power

The BLE transmit power significantly affects energy consumption and connectivity: higher outputs extend the range but reduce battery life. Current measurements during advertising identified -4 dBm as optimal: lower power yielded negligible savings while maintaining a non-line-of-sight range of ≥10 m in a workplace environment (Supplementary Fig. 16d). Thus, -4 dBm was used in all subsequent characterizations.

#### BLE conn_eff_ interval

Using a 100 ms connection interval, current consumption versus BLE conn_eff_ interval during deep sleep revealed a 4 s ‘knee point’ optimum (12.7 µA) for balancing lifetime and latency (Fig. 5E). Therefore, low-latency data transfer following processing events is achieved, with power savings elsewhere (Fig. 6).

#### MTU size

BLE transmissions suffer from overhead due to radio startup/shutdown and protocol headers. Accordingly, the MTU was negotiated to its maximum value (247 bytes here, although some devices permit 512 bytes) – alongside the data length extension parameter – to minimize the total number of transmission events while fitting the data into a single packet. Accordingly, the radio on-time, packet count, and total energy are minimized. This approach incurs no power penalty when transmitting shorter data since packets are not padded when the payload is smaller than the MTU.

#### Processing interval

Similar to BLE transmission, processing incurs additional power overheads from the MCU and SPI startup. Supplementary Fig. 18e-j shows that although processing and transmission durations increase with data size, the growth is nonlinear and diminishing, suggesting that batching data into fewer operations will have a significant impact on average power. The processing interval was optimized through an active mode study under three sampling interval configurations (Supplementary Fig. 19). As processing and transmission overheads become less frequent, the average currents flatten exponentially, showing optimum knee point transition regions followed by asymptotic behavior. While the processing knee time shifted with sampling intervals, normalizing to the sampling interval and plotting against the FIFO threshold revealed consistent alignment across cases, with a threshold of 10 effectively suppressing overheads without excessive latency. This 10× scaling of processing relative to sampling intervals was carried through all subsequent measurements (Fig. 6 and Supplementary Table 6).

#### Digital filter decimation factors

Increasing the AFE’s digital filter decimation reduces the ADC’s sampling rate, improving precision through stronger noise rejection, at the cost of longer conversion time and higher power, since the ADC must stay active longer to produce one output sample. In wearables, where battery lifetime is critical, this filtering (e.g., smoothing) is better offloaded to the host, where the power penalty is negligible. Thus, the sinc3 and sinc2 decimation factors were configured modestly (4 and 44, respectively) for all measurements.

### Statistical analysis

Data and statistical analyses (including linear regressions, coefficients of determination, Deming regressions, Pearson’s correlation coefficient calculations, and error analysis) were performed in MATLAB. Data displayed with error bars were collected from three independent experiments. Error bars represent one standard deviation.

### Reporting summary

Further information on research design is available in the Nature Portfolio Reporting Summary linked to this article.

## Supplementary information

Supplementary Information

## Data Availability

The wearable device hardware, firmware, software, and mechanical fixture documentation are available open-access at: https://github.com/ProfDrewHall/Biocoin. All raw datasets supporting the findings of this study are available from the corresponding author upon reasonable request. Source data are provided with this paper.

## Code availability

The code used to analyze the raw datasets and generate statistical analysis results was developed in MATLAB v2023b and is available from the corresponding author upon reasonable request.
