# Supplementary material for: Biocoin: an open-source wearable platform for multiplexed and multimodal biosensing
Source: NPJ Biosens. 2026 May 27;3(1):44. doi: 10.1038/s44328-026-00103-z (PMC13433255; doi:10.1038/s44328-026-00103-z)
Supplement: Supplementary file 1 [file 44328_2026_103_MOESM1_ESM.pdf]

Table of Contents:

Supplementary Note 1. Justification for the choice of system power supply and wireless protocol.

Supplementary Note 2. Additional fabrication, component, and assembly details of the wearable system.

Supplementary Note 3. Electrical characterization of the system's various measurement methods and power consumption.

Supplementary Note 4. Additional design considerations and circuit details of the device's analog subsystems.

Supplementary Note 5. Iontophoresis current source design considerations, circuit operation, and comparative discussion with prior art.

Supplementary Note 6. Potentiostat compliance voltage, CE clipping, and TIA gain tradeoffs/challenges.

Supplementary Note 7. Additional system power management considerations and justifications.

Supplementary Note 8. Electronic system modes of operation.

Supplementary Note 9. High-frequency sampling operation, BLE throughput, and worst-case system power consumption.

Supplementary Figure 1. Photographs of the Biocoin and docking station subcomponents.

Supplementary Figure 2. 3D CAD model images of the 3D-printed fixture components.

Supplementary Figure 3. BLE RSSI vs. distance with and without battery placement.

Supplementary Figure 4. Amperometric glucose sensing in artificial interstitial fluid (ISF).

Supplementary Figure 5. Measurement results of the impedance sensing engine.

Supplementary Figure 6. Schematic and measurement results of the battery voltage monitor.

Supplementary Figure 7. Current consumption of the battery monitoring and status LED housekeeping operations.

Supplementary Figure 8. Schematic illustration and examples highlighting the challenges in a TIA-based potentiostat regarding compliance voltage, TIA gain, and amplifier clipping.

Supplementary Figure 9. Flow chart of the wearable device's firmware operation.

Supplementary Figure 10. Firmware flow chart specific to the AD5940's operation during measurement routines.

Supplementary Figure 11. Implementation diagrams of the different electrochemical and impedance measurement methods supported by Biocoin.

Supplementary Figure 12. Example waveform oscilloscope captures of the six core electrochemical methods: CA, CV, DPV, SWV, LSV, and NPV.

Supplementary Figure 13. Illustration of the device firmware's class-based organizational structure.

Supplementary Figure 14. BLE operation and custom GATT server configuration.

Supplementary Figure 15. Custom Python script and graphical user interface for wireless BLE control of Biocoin.

Supplementary Figure 16. Device startup and measurement sequence example routine and characterization during BLE advertising.

Supplementary Figure 17. System modes of operation overview used for power consumption optimizations.

Supplementary Figure. 18. Illustration of the device's transient current consumption while performing different active mode operations with varying parameters.

Supplementary Figure. 19. Optimization of the data processing interval parameter.

Supplementary Figure 20. Biocoin schematic: power management sheet.

Supplementary Figure 21. Biocoin schematic: MCU and BLE module sheet.

Supplementary Figure 22. Biocoin schematic: analog front-end sheet.

Supplementary Figure 23. Biocoin schematic: iontophoresis current source sheet.

Supplementary Figure 24. Docking station schematic: power management sheet.

Supplementary Figure 25. Docking station schematic: digital signal interface sheet.

Supplementary Figure 26. Docking station schematic: analog signal interface sheet.

Supplementary Table 1. Comparison table of state-of-the-art wireless electronic systems for skin-interfaced biochemical sensing.

Supplementary Table 2. Comparison table of open-source potentiostat-based electrochemical systems.

Supplementary Table 3. Summary of all electrochemical, resistive, and impedance measurement methods performed by Biocoin, and the supported values and ranges of their associated parameters.

Supplementary Table 4. Comparison of low-power BLE-compatible microcontroller chipsets (modules) considered during the Biocoin design phase.

Supplementary Table 5. Comparison of dedicated electrochemical front-end chipsets considered during the Biocoin design phase.

Supplementary Table 6. Summary of the wearable device's average current consumption and estimated battery lifetime during different modes and states of operation, with varying operational parameters and analog supply voltages ($V_{DD,A}$).

Supplementary Table 7. Bill of materials for all components on the Biocoin PCB ($V_{DD,A}$ = 3.6 V).

Supplementary Table 8. Bill of materials for all components on the Biocoin PCB ($V_{DD,A}$ = 3.0 V).

Supplementary Table 9. Bill of materials for all components on the docking station PCB.

Supplementary Table 10. Detailed cost breakdown of the complete Biocoin system at varying production volumes, and comparison with representative commercial potentiostat platforms.

Supplementary Note 1. Justification for the choice of system power supply and wireless protocol.

The choice of power supply and wireless communication protocol for a wearable device has significant implications for the system's operational lifetime, range, and form factor. Beginning with the power supply, the most commonly adopted energy source among wearable biochemical sensors over the last decade has been Li-Ion/polymer batteries. These sources provide high energy densities (large energy storage capacities within a given area) for a long-lasting electronic lifetime, can be easily recharged for repetitive use, and are available at low cost off the shelf. In addition, the large energy densities (>100 mW/cm^2) enable the electronics to support complex processes, including electrochemical analysis, data processing, and wireless transmission/reception, which can all demand large instantaneous current draws (>10 mA) from the battery. As such, opting for a Li-Ion/polymer battery imposes little to no restrictions on the functionality of the wearable device. Nevertheless, lithium batteries are not without downsides: they have bulky and rigid form factors, use hazardous and non-biodegradable materials (*e.g.,* lithium salts, cobalt), and pose safety concerns for potential overheating or puncture scenarios.

To tackle these challenges, extensive research has been conducted on various energy-harvesting methods to develop more sustainable and conformable power sources for battery-free wearables. Several notable examples include biofuel cells (BFCs)[1], triboelectric nanogenerators (TENGs)[2], thermoelectric generators (TEGs)[3], moisture-enabled electricity generators (MEGs)[4], perovskite solar cells[5], sweat-activated batteries (SABs)[6], and Wi-Fi energy harvesting[7]. Unfortunately, the primary challenge shared by all of these energy harvesting methods is their limited power densities (<10 mW/cm^2), which can either prevent them from powering the most demanding electronic functions within a wearable system – such as wireless communication or signal processing – or necessitate slower sampling and communication rates, leading to increased latency. For BFCs in particular, an additional challenge is their long-term stability, which is limited in practice by biofouling. Since BFCs, SABs, and TENGs all require either human sweat or motion for energy harvesting, rigorous physical exercise is necessary for their operation. As a result, these approaches lack power continuity during everyday sedentary activities (such as working, sleeping, eating, and lounging), making them less suitable for continuous monitoring applications. To that end, powering methods based on ambient light, moisture, or Wi-Fi harvesting are attractive because they do not require human input; however, their performance remains dependent on environmental conditions that may fluctuate (*e.g.,* indoor vs. outdoor, day vs. night, urban vs. rural). Among these techniques, the longest reported continuous device operation time was 60 hours for the biofuel cell[1], followed by 12 hours for the perovskite solar cell[5].

After carefully weighing these tradeoffs in the current landscape of battery and energy-harvesting research, we opted for a rechargeable Li-ion battery in this work to produce a general-purpose wearable platform that supports as wide a range of applications as possible. Following a survey of commercially available battery options, the RJD2430C1ST1 (Cornell Dubilier/Illinois Capacitor) was specifically selected for its excellent balance between high capacity (110 mAh) and minimal form factor (24.5 mm diameter and 3.55 mm thickness). Given Biocoin's ultra-low power operation, this type of capacity enables operation (even with continuous wireless communication) for weeks to months, depending on measurement sampling rates, mitigating device 'downtime' and removing the burden of frequent battery recharges. When required, the docking station provides the necessary infrastructure for this recharging. Note, however, that the battery connector and system power management are flexible, allowing for the integration of any ~3.7 V battery into this system, depending on application size, weight, or lifetime considerations.

Regarding wireless communication, variants of the Bluetooth (BL) protocol are by far the most popular among wearable biochemical devices. Early fully integrated device prototypes used the Bluetooth Classic protocol, while newer designs have transitioned to Bluetooth Low Energy (BLE), which is now the gold standard for these types of systems. The operation of BL classic is most closely comparable to that of a serial universal asynchronous receiver/transmitter (UART), in which both the receiver (Rx) and transmitter (Tx) are always on, listening for incoming data. This type of scheme achieves low-latency communication at the expense of high average current

consumption (>10 mA). BLE, on the other hand, leverages the lower data rates of wearable devices to reduce average power consumption dramatically. By optimizing the BLE connection interval and slave latency parameters, average currents on the order of 10–100 μA can be achieved for latencies ranging from seconds to a few milliseconds, which are acceptable for biochemical applications. Importantly, both BL classic and BLE can support communication distances of >10 m with a host device in typical environments.

Another wireless protocol that has gained popularity for wearable biosensors is near-field communication (NFC)[8–11]. NFC provides simultaneous power and data communication to a wearable device via inductive coupling between the wearable and an external transponder (which is typically a smartphone reader). When the transponder is placed in close proximity to the wearable device (~4 cm or less), radio-frequency (RF) power is harvested via an inductive link, enabling battery-free operation, albeit with a reduced maximum allowable current draw compared to batteries. NFC, therefore, represents a solution to eliminate the need for bulky, hazardous batteries. However, the requirement of manually bringing a transponder into close proximity to the NFC wearable makes their operation “on-demand” and prevents fully autonomous sensing. To avoid placing the burden of device power and sensor sampling on the user, while also supporting longer range and truly continuous functionality, we opted for the BLE communication protocol in this design.

Supplementary Note 2. Additional fabrication, component, and assembly details of the wearable system.

Biocoin. The electronic system components can be categorized into four main areas: analog sensor interface, iontophoretic sweat stimulation, data processing and wireless communication, and power management. The components that interface with the analog sensors consist of a fully integrated analog front-end (AFE, AD5940BCBZ-RL, Analog Devices), a low-leakage 4:1 multiplexer (MUX, TMUX1104DQAR, Texas Instruments), four voltage buffers using discrete amplifiers (2× TLV8542RUGR, Texas Instruments), and up to three physical sensors implemented as resistive voltage dividers. A zero-insertion-force (ZIF) connector (5034802000, Molex) located on the bottom of the printed circuit board (PCB) provides the electrical interface between Biocoin and the wearable electrode patch. Data processing and wireless communication are controlled by a BLE-compatible microcontroller (MCU) module (MDBT50Q-1MV2, Raytac) based on the nRF52840 system-on-chip (SoC) from Nordic Semiconductor, which includes an on-board chip antenna. Iontophoresis is enabled through a programmable voltage-controlled current source implemented using an operational amplifier (opamp, OPA990IDCKR, Texas Instruments) and a current sense amplifier (CSA, MAX4372TEBT+T, Analog Devices). Overcurrent protection is ensured through a junction field effect transistor (JFET) current limit circuit (TF412ST5G, Onsemi) and two discrete transistor switches (DMP32D5SFB-7B, DMN62D1LFB-7B, Diodes Inc.). The device is powered by a rechargeable 3.7 V 110 mAh Li-Ion coin cell battery (RJD2430C1ST1, Cornell Dubilier/Illinois Capacitor), whose voltage level is monitored using a resistive voltage divider and an opamp buffer (MAX44264EWT+T, Analog Devices). The system power management further comprises a high-efficiency boost converter (MAX17291ANT+T, Analog Devices), a multi-output buck-boost converter (MAX17270ENE+T, Analog Devices), a low-dropout linear regulator (LDO, TPS7A2036PDQNR for 3.6 V analog supply operation or TPS7A0230PDQNR for 3.0 V analog supply operation, Texas Instruments), and a pair of load switches (CSD75208W1015, Texas Instruments).

Docking station. The docking station PCB was fabricated by JLC PCB on a 4-layer rigid FR4 substrate measuring 35.18 cm^2 (5.33 cm x 6.6 cm) and 1.6 mm thick (designed using Altium Designer version 23.0.1). Components were sourced from DigiKey Electronics or Mouser Electronics and soldered onto the docking station. Similar to Biocoin, the docking station schematics and bill of materials (BoM) are provided in **Supplementary Figs. 24-26** and **Supplementary Table 9**, respectively, with all other hardware files available in the GitHub repository.

The docking station comprises: 1) a battery charging circuit implemented with a Li-Ion battery charger (MCP73831T-2ACI/OT, Microchip Technology), power diode (MBR120ESFT3G, Onsemi), and a P-channel metal oxide semiconductor field effect transistor (MOSFET, DMG2305, Diodes Inc.), 2) two sets of spring-loaded 8-pin headers (POGO, 855-22-008-10-001101, Mill-Max Manufacturing Corp.) and two sets of 10-pin headers (POGO, 855-22-010-10-001101, Mill-Max Manufacturing Corp.), 3) two standard 3-pin screen-printed electrode (SPE) connectors (DS1020-03RT1D, Connfly) for *in vitro* electrochemical tests, 4) screw terminals (1725708, Phoenix Contact) for versatile connection to other types of electrodes/equipment, 5) electrochemical mock cells built using *RC* circuit models for electrochemical method validation and debug, 6) a serial wire debug (SWD) header, 7) a power on-off switch (1101M2S3CQE2, C&K), 8) an auxiliary battery connector (S2B-PH-K-S(LF)(SN), JST Sales America Inc.), 9) test hooks connected to the positive and negative battery terminals that permit the device to be powered/measured using external equipment (5010, 5011, Keystone Electronics), and 10) a micro-universal serial bus (USB) connector.

To facilitate electrical connection between Biocoin and the docking station, the majority of the analog signals that are sent into the electrode connector on the underside of Biocoin are also routed to sets of exposed metal pads (**Fig. 2c**). These pads connect to the POGO pin headers located on the docking station with the aid of 3D-printed fixtures. All other signals on Biocoin required for device programming, recharging, and serial communication are also sent to different sets of exposed pads (such as power, ground, USB, SWD, *etc.*) (**Fig. 2c**). **Supplementary Fig. 1b,c** presents annotated images of the docking station features and highlights the assembly steps between the docking station, the fixtures, and Biocoin.

Additionally, the auxiliary battery connector on the docking station allows the two PCBs to be powered by a higher-capacity battery, significantly extending their operational lifetime. When coupled with standard SPE connectors, the device can also serve as a credit card-sized, portable electrochemical analyzer, offering comparable performance to commercial potentiostats at a reduced cost. The utility of this system is thereby extended beyond wearable applications and rivals, or even exceeds, the functionality of prior-art portable potentiostats thanks to the wide array of implemented electrochemical methods, wireless BLE communication, and power-optimized firmware outlined in this manuscript[12–24].

Battery recharging. The battery charging circuitry is implemented on the auxiliary docking station PCB rather than on the wearable Biocoin board itself. The docking station connects to a USB power source and contains the battery charging IC, while the wearable PCB plugs into the docking station for charging via the POGO pins. This strategy was intentionally selected to reduce the size, thickness, and power overhead of the wearable device. Integrating charging circuitry or a USB connector directly onto the wearable PCB would increase component count and occupy valuable board area within the constrained coin-sized form factor. Similarly, wireless charging would require an additional receiver coil, increasing the device thickness. By relocating the charging circuitry to the docking station, these components are removed from the wearable electronics during normal operation while maintaining a simple charging workflow in which the device is inserted into the USB-powered dock. Given the ultra-low power consumption of the system, which enables weeks-to-months of battery lifetime depending on the sensing modality (**Fig. 6** and **Supplementary Table 6**), it is likely that sensing chemistry, rather than battery lifetime, will limit the overall wearable lifetime.

Electrode patch. A wearable multiplexed electrode patch prototype was designed on a flexible 2-layer PCB with a polyimide substrate and an immersion-gold surface finish. The PCB was fabricated by PCB Minions and measures 9.97 cm^2 (4.907 cm × 2.032 cm) with a thickness of 0.235 mm to support enhanced flexibility, allowing it to be wrapped underneath the Biocoin PCB within a wearable fixture. Altium Designer (version 23.0.1) was used for schematic and layout design. For illustrative purposes, three working electrodes (WEs), one reference electrode (RE), and one counter electrode (CE) were included with an iontophoresis anode and cathode electrode pair surrounding them in a circular geometry. A footprint for a surface-mount thermistor element was also included on the back of the PCB to serve as an example skin-temperature sensor. The electrode patch serves as proof of concept that the ZIF connector on Biocoin provides a general-purpose connector solution compatible with standard PCB technologies, suitable for low-cost and/or mass production. Photographs of the electrode PCB are shown in **Fig. 2d** and **Supplementary Fig. 1a**.

3D-printed fixtures. The Biocoin casing for wearable operation consists of two fixture pieces, denoted the top and bottom cases (**Supplementary Figs. 1a and 2**). Two variants of the top case were designed (with heights of 7.366 mm and 9.525 mm, respectively), providing support for batteries with greater thickness and, by extension, greater capacity. By default, all measurements and figures presented in this manuscript assume the smallest top case is used (supporting the 110 mAh RJD2430C1ST1 battery) to favor user comfort by minimizing device bulkiness and weight. However, if additional battery lifetime is required for a given application, the larger top case can support a 150 mAh battery (RJD2440ST1).

The fixtures for the docking station also consist of a top and bottom piece that are used to align the exposed pads under Biocoin with the POGO pin headers (**Supplementary Fig. 1c and 2**). The bottom fixture slots into the docking station via cutouts for the POGO pin headers. Biocoin mounts into the bottom fixture via two vertical posts that insert into mounting holes on the underside of the wearable device. The top fixture then sandwiches Biocoin to the bottom fixture and docking station via four sets of machine screws and nuts (91841A038 and 91772A170, McMASTER-CARR) that run through mounting holes located in each component. This press-fit solution, using exposed metal pads, enabled a secure docking-station connection while eliminating the need for an additional bulky connector on Biocoin.

Assembly of the system components for wearable operation. The wearable device hardware consists of six primary components (**Supplementary Fig. 1a**). The battery cable is established through a miniaturized receptacle (ACHR-02V-S, JST Sales America Inc.) and a pre-crimped lead (ASACHSACH28W51, JST Sales America Inc.). One side of the cable is soldered directly to the battery terminals, while the other plugs into the corresponding connector on Biocoin (BM02B-ACHSS-GAN-TF, JST Sales America Inc.). The bottom case fixture secures to Biocoin in the same manner as described for the docking station. The electrode patch plugs into Biocoin's ZIF connector and can be wrapped underneath the PCB and through the bottom case, exposing the sensors. Upon alignment, small notches in the top case wall allow the four prongs of the bottom case to enter and secure the connection. For adhering the wearable hardware to the skin, a double-sided medical adhesive (1509, 3M) can be used. Various assembled images of the wearable device are depicted in **Fig. 2d**.

Supplementary Note 3. Electrical characterization of the system's various measurement methods and power consumption.

During execution of a given measurement task, analog input signals from various external sensors are obtained and processed by the different front-end circuits provided on Biocoin. These signals are subsequently digitized by the internal analog-to-digital converters (ADCs) of either the AD5940 or the nRF52840, depending on the specific measurement. The data are then buffered and stored locally in the MCU before being transmitted to a host device, either wirelessly via BLE during wearable operation or via a USB serial connection if installed in the docking station. For all measurements described in this section, unless otherwise mentioned, Biocoin was connected to the docking station to permit connection to external equipment and sensors for characterization purposes.

Potentiometric measurements. The potentiometric channels implement open-circuit potentiometry (OCP), an electrochemical method (**Fig. 4a**). The circuit was characterized first by measuring its zero-frequency (DC) transfer function (**Fig. 4c,d**). Through the docking station screw terminal headers, known DC input voltages were applied to the OCP electrode inputs (node labeled V_{IN}) from a precision benchtop source measure unit (SMU, 6430, Keithley). The output voltage V_{OCP} was measured with respect to the RE common-mode voltage (V_{RE}) as V_{IN} was swept across the AD5940's ADC input common-mode range. The measurement was repeated for three different RE voltages, which was enabled by reprogramming the digital-to-analog converter (DAC) output voltage. The input resistance of the OCP channels was characterized using the same measurement procedure as the DC transfer function, except now the PCB leakage current was read out from the SMU (leveraging the 6430's sub-femtoampere precision) (**Fig. 4e**). Due to the presence of two PCBs in this measurement setup, Biocoin and the docking station, two leakage current measurements were performed. The first included both PCBs installed together, while the second included just an empty docking station. The difference between the two measured currents represents the leakage current of Biocoin itself.

Resistive sensor (temperature) measurements. The resistive sensor channels, intended for measuring physical parameters such as skin temperature or strain, were characterized in two ways (**Fig. 4a**). First, known input voltages from a precision SMU (B2912A, Keysight) were applied directly into the AD5940's internal MUX and ADC inputs via the V_{TEMP} node (once again through the docking station screw terminal headers) (**Fig. 4f,g**). Note that the input resistive voltage dividers, consisting of R_2 and $R_{3,}$ were not populated for this test. The voltage V_{TEMP} was then swept across the ADC's input common-mode range and measured. Second, R_2 and R_3 were reinstalled to form a voltage divider, with R_2 a known 100 kΩ resistor on Biocoin and R_3 implemented with a surface-mount linear thermistor (TMP6331QDYARQ1, Texas Instruments) on the flexible electrode patch (**Supplementary Note 2**). The temperature performance of the sensor was characterized by placing the electrode patch on a temperature-controlled hot plate (76549-914, VWR) and measuring V_{TEMP} at 60-second intervals across temperatures ranging from 25 to 50˚C (**Fig. 4h,i**). Accurate temperatures were ensured by calibrating the hot plate using an infrared thermometer (LASERGRIP 800, ETEKCITY). This measurement was performed outside the docking station, allowing the electrode patch to be plugged into the ZIF connector. As such, sensor readings were transmitted back to a host computer via BLE.

Impedance measurements. Impedance (or admittance) sensors are commonly used in wearable sweat sensors for monitoring sweat rate and sensor calibration. Using the high-speed excitation loop and discrete Fourier transform (DFT) engine inside the AD5940, impedance measurements were conducted by characterizing external through-hole resistors of a known value that were inserted between the appropriate AD5940 analog inputs sent down to the docking station (**Supplementary Fig. 5**). A 4-wire measurement configuration was used to eliminate stray resistance associated with routing and/or cable interconnects. Resistors ranging from 10 Ω to 100 kΩ were measured, corresponding to admittances of 100 mS to 10 µS, respectively. To keep the measured current within an acceptable range for the transimpedance amplifier (TIA), the 100, 10, and 1 kΩ resistors were measured with a 600-mV sine wave amplitude (E_{ac}), while the 100 Ω resistor was measured with E_{ac} = 60 mV and the 10 Ω resistor was measured with E_{ac} = 6 mV. A stimulation frequency of 10 kHz was used for all

measurements. The TIA gains were manually adjusted for each resistor to ensure the TIA output voltage remained within the acceptable ADC input common-mode range. The "true" resistor values were determined using a precision benchtop *LCR* meter (E4980A, Keysight) with identical E_{ac} and frequency settings as those used on Biocoin for each resistor.

Iontophoresis measurements. The functionality of the iontophoresis voltage-controlled current source was measured by connecting through-hole resistors (R_{load}) of different values between the anode and cathode electrodes (via the screw terminals on the docking station), which mimic the load impedance presented by the skin during wearable operation (**Fig. 4b**). The circuit's DC transfer function was characterized by sweeping the input control voltage from the AD5940 DAC (V_{DAC}) and measuring the current (I_{load}) delivered to the resistive loads via the CSA output and built-in MCU ADC (**Fig. 4j-l**). The transfer function was measured at different values of R_{load} ranging from 5 to 40 kΩ. Short and open measurements were also taken. The current-source compliance voltage was set to 20 V for all measurements via the boost converter's output-voltage configuration.

Battery monitoring measurements. Battery voltage monitoring was functionally verified by applying known input voltages directly to the positive battery terminal on Biocoin (V_{BAT}) and measuring the output of the resistive voltage divider and opamp buffer sensing circuit using the MCU's built-in ADC (**Supplementary Fig. 6**). The voltages were applied from an external SMU (B2912A, Keysight) into the power management test hooks on the docking station (**Supplementary Fig. 1b**) and swept across a 3.0 to 4.2 V range to mimic the usable operating range of the employed Li-Ion battery.

Electrochemical method waveform validation. Biocoin supports eight electrochemical methods, including chronoamperometry (CA), cyclic voltammetry (CV), differential pulse voltammetry (DPV), square wave voltammetry (SWV), linear sweep voltammetry (LSV), normal pulse voltammetry (NPV), open-circuit potentiometry (OCP), and electrochemical impedance spectroscopy (EIS) (**Supplementary Table 3**). Each method was implemented by programming the AD5940 sequencer and by appropriately configuring the different analog front-end (AFE) blocks. The implementation of the time-varying voltage waveforms applied to the electrochemical cell (V_{WE}, V_{RE}, and $V_{WE} - V_{RE}$), as well as the timing of data-ready interrupt signals sent back to the MCU, were validated by probing the various circuit nodes with a digital oscilloscope (MSOS254A, Keysight) and comparing them head-to-head with a commercial potentiostat (PalmSens4, PalmSens) under identical parameter settings (**Supplementary Fig. 12**).

Power consumption measurements. Depending on the tasks Biocoin needs to execute at a given time, the device's instantaneous current consumption varies dramatically by several orders of magnitude. In the deepest sleep states, the PCB consumes single-digit µA of current for prolonged periods, whereas high-power operations, such as ADC sampling or BLE radio events, consume >10 mA for brief ms-level intervals. Accordingly, accurately measuring the device's instantaneous current consumption profiles as it switches between different modes of operation requires instrumentation with >80 dB of current measurement dynamic range (DR) and at least 10 kHz of bandwidth. For characterization purposes, Biocoin was powered at 3.7 V by an SMU (B2912A, Keysight) connected to the docking station test hooks to emulate battery-powered operation (**Supplementary Fig. 1b**). Since Biocoin employs switching voltage regulators (buck-boost and boost converters) to manage the battery supply, its current draw from the battery will dynamically increase as the Li-Ion decays from 4.2 V (fully charged) down to 3.0 V (empty), based on conservation of total power. However, since 3.7 V serves as the nominal working voltage of a Li-Ion battery, around which the device will spend the majority of its lifetime, it is therefore a strong representation of the average current that the PCB will consume. For this reason, all current consumptions reported in this work were taken with the electronic system powered at 3.7 V. Since the supply voltage is always kept constant for this characterization, 'power consumption' data are therefore presented as 'current consumption' in this manuscript, as it provides the easiest translation into battery lifetime for a given capacity (in this case, 110 mAh). Additionally, all current measurements herein, except **Fig. 6f**, represent quiescent current consumption values as they were taken with no external loads or sensors connected to the device.

To support the aforementioned specifications, all current measurements were performed using a power profiling device (PPK2, Nordic Semiconductor) set to ampere meter mode, placed in series with the SMU. The device features current auto-ranging, allowing precise instantaneous measurements across all modes of operation (deep sleep to active). For accurate display of all transient events, the current profiles shown in this work were collected using the maximum sampling rate of 100 kSps, which was sufficient to capture the rapid current spikes associated with the on-board switching regulators and BLE/ADC events (**Figs. 5b-d and 6a,d,e** and **Supplementary Figs. 16a-c and 18**). However, reporting accurate average current values of a given transient profile requires taking the average over long time periods to achieve strong statistical confidence (*i.e.,* longer than the 20 s profiles shown for illustration). As such, average values were reported for transient profiles that spanned over several minutes during deep sleep (**Fig. 5e**), BLE advertising (**Supplementary Fig. 16d**), and iontophoresis (**Fig. 6f**), or at least 20 processing intervals worth of time during active mode (**Fig. 6b,c**, **Supplementary Fig. 19**, and **Supplementary Table 6**), and the measurements were collected at 10 kSps to reduce the amount of collected data and avoid excessively large files. Notably, no differences were observed in the average values between measurements taken at the two sampling rates. The measured data were visualized and saved using the Power Profiler App (v4.2.2, Nordic Semiconductor) running on a host computer connected to the PPK2 via USB. Note that for accurate current measurements under wearable operational conditions, the USB cable must be disconnected from the docking station, and all other components that could otherwise draw static quiescent current (*e.g.,* the battery charger) must be removed. In this manner, the measured currents correspond solely to that consumed by Biocoin.

Finally, it is worth noting that, since switching regulators power the MCU, AFE, and iontophoresis, the system naturally exhibits transient current consumption profiles that do not necessarily visually reflect the instantaneous current drawn by the various integrated circuits (ICs). For example, even though the AFE draws a static DC current of a few µA during its hibernate state – at the outputs of the buck-boost converter and subsequent LDO (**Fig. 5a**) – the input current to the buck-boost during this state may still exhibit large current spikes (mAs) followed by periods of near-zero current draw. These current spikes occur at varying frequencies according to the employed switching regulator's load current demands, making it hard at times to visually distinguish between spikes caused by these switching artifacts and 'real' scheduled device operations (BLE Tx/Rx, ADC sampling, *etc.*). To substantially reduce the frequency of extraneous current-switching spikes, a 100 µF bulk capacitor was placed at the output of the PPK2 instrument for all current measurements. The capacitor behaves as a large energy storage element, allowing the buck-boost converter to draw more instantaneous current at its input with a single switching event, thereby enabling it to supply its output load for longer periods before initiating another switching event. Occasionally, some residual current spikes from the buck-boost converter can still be seen, as evidenced in **Figs. 5b and 6a**, where a few mA-level spikes appear even though the system is in an idle or hibernate state during these instances. Outside of these infrequent spikes, the current only exhibits small ripple characteristics (**Figs. 5c and 6d**). This capacitor addition was made solely to improve visual clarity in the plots, enabling corresponding current profiles that more closely correlate with MCU or AFE activities and providing a better representation of the sleep-mode current floors. Since the bulk capacitor is a purely passive component, it makes no difference to the measured average values or battery lifetime estimates.

Supplementary Note 4. Additional design considerations and circuit details of the device's analog subsystems.

Regarding the additional functions added to Biocoin's AFE, it is important to note that their primary function is to expand the array of multiplexed measurable inputs beyond the AD5940's native capabilities. In any densely integrated sensing platform, there is always a potential for unwanted inter-electrode crosstalk. As the physical spacing between electrodes decreases in compact wearable formats, diffusion-mediated crosstalk and electrolyte coupling may arise depending on electrode geometry and the specific sensing chemistry. In this platform, however, multiple electrodes are interfaced using time-division multiplexing, where only one electrode is actively biased at a time while others remain in a high-impedance state. In particular, the selected 4:1 multiplexer (TMUX1104) placed on the AFE's WE node features extremely low leakage current in its off-state configuration (<15 pA across all voltage biasing conditions), providing strong electrical isolation. As a result, sensor crosstalk, potential coupling, and simultaneous faradaic processes occurring across multiple electrodes can all be greatly mitigated with this approach.

Additionally, note that an external WE MUX is not inherently necessary to permit multiplexed current inputs with the AD5940, due to the chip's internal switch matrix. However, all additional auxiliary analog input pins were already occupied for other functionalities (OCP, resistive sensors, and impedimetry). Regardless, the employed MUX occupies only 1 mm × 2.5 mm and features extremely low leakage (<15 pA) that is negligible compared to that of the AFE's own internal switch matrix (>300 pA).

To that end, the OCP channels could have also been realized by reconfiguring the AD5940's internal potentiostat amplifiers via the switch matrix. Nevertheless, incurring an input leakage current penalty of >300 pA is unacceptable for potentiometric applications. OCP electrodes can present large source impedances to the measurement circuitry and must operate in a *zero-current condition*, thereby requiring voltage buffers with exceptionally large input impedances (≥ GΩs)[25]. These buffers aid in measuring the open-circuit potential between a working electrode (labeled as $OCP_{1\text{-}4}$) and a reference electrode, allowing the AD5940's ADC to digitize the differential voltage ($V_{OCP} – V_{RE}$) (**Fig. 4a**). Ultra-low input bias current (100 fA) amplifiers were utilized for this objective to minimize voltage readout errors. The selected packages occupy just 1.5 × 1.5 mm^2 of PCB area and consume 500 nA per amplifier (2 µA total), minimizing their additional system overhead. Referring to the measured OCP channel input resistance of 115 GΩ, this result highlights a significant, albeit expected, increase in total leakage current due to the PCB substrate compared to that of the 100 fA input bias current amplifiers in isolation (**Fig. 4e**). Further improvements to input resistance could be obtained, if required, by increasing the PCB dielectric layer thickness or utilizing a lower loss substrate material (in exchange for increased device height or fabrication cost, respectively). Prior to conditioning and digitization by the AD5940's ADC chain, a first-order *RC* low-pass filter (LPF) with a cutoff frequency of ~32 Hz is placed in between each buffer output and the MUX input to further reduce measurement noise since OCP sensors operate near-DC.

For each resistive sensor (**Fig. 4a**), the resistor (R_2) represents a component located on the PCB with a known fixed resistance value, whereas the variable resistor (R_3) represents an element located on the wearable patch whose resistance changes proportionally to the desired physiological parameter. The output of the voltage divider (V_{TEMP}) is digitized by the AFE's differential ADC relative to a precision internal mid-scale reference voltage (V_{MID}), enabling quantification and back-calculation of the equivalent resistance change.

Finally, regarding impedance analysis capabilities, to sense the impedance of an unknown load using either the 2- or 4-wire measurement method, the load should be placed between the auxiliary IMP pins and the CE pin. Measurements using electrochemical impedance spectroscopy (EIS) can reuse the WE, RE, and CE pins. Compared to a 2-wire setup, a 4-wire configuration eliminates errors caused by electrode and interconnect routing parasitics.

Supplementary Note 5. Iontophoresis current source design considerations, circuit operation, and comparative discussion with prior art.

Upon surveying early examples of published wearable iontophoretic delivery systems, it is observed that most works employ stimulation currents in the range of 100s of μA to 1.5 mA to meet the necessary sweat rate and sweat duration requirements of a given application[26–30]. When coupled with the fact that the skin (and electrode-gel-skin interface) can present a relatively large impedance (~10s of kΩ), it becomes apparent that a large DC voltage can develop across the stimulation skin electrodes, necessitating that the current source operates with a large compliance voltage. For example, 1 mA passing through a 10 kΩ skin impedance mandates a 10 V compliance. Given the limited available battery voltage (~3.7 V), some form of boost conversion is therefore required to supply the current source and achieve this compliance, which can contribute significant additional power consumption and area. The full set of current source design considerations can therefore be summarized as follows: 1) support stimulation currents up to 1.5 mA, 2) generate a compliance voltage >10 V, 3) provide digital programmability of the current to permit individual sweat rate optimization, 4) integrate multiple protection features to avoid overcurrent scenarios that could burn or otherwise cause discomfort to the user[31], 5) consume minimal quiescent power, and 6) occupy a small PCB footprint.

Prior art wearable iontophoresis solutions have struggled to address these challenges simultaneously. A popular current-source architecture, introduced in 2017 and widely used thereafter, leverages a unity-gain difference amplifier and a bipolar transistor to source the iontophoretic current[26,27,32,33]. The design is programmable via voltage control, provides high-accuracy current generation via the amplifier's integrated matched resistors, and includes several safety features. However, >30 mA were drawn from the battery to perform iontophoresis, stemming from inefficient DC-DC conversion at the targeted load current and the large quiescent currents required to drive the amplifier's resistive feedback. Additionally, since the control voltage typically comes from an MCU's DAC output, an auxiliary voltage buffer may be required to drive the resistive input terminal, further increasing the PCB footprint. An elegant approach, utilizing a dedicated floating current source and two charge pumps to generate the large compliance voltage, was also developed[28]. Using charge pumps enabled higher efficiency at lighter loads, and the truly floating nature of the current source eliminated additional quiescent current paths, reducing the total battery draw to ~10 mA. Unfortunately, programmability was not addressed, and the adoption of more specialized components (*i.e.,* charge pumps) mandated the use of IC package footprints that substantially increased the PCB size. More recently, a compact current mirror-based solution has been proposed that consumes very low quiescent current (estimated at ~450 μA at an 18 V compliance) and reuses the TIA and ADC within the AD5940 for overcurrent monitoring protection[5,34]. Nevertheless, the architecture was not programmable or easily modifiable (with minimal area impact) to satisfy this objective. To mitigate the power consumption penalty of iontophoresis, research into zero-power osmotic sweat extraction has also been conducted, leveraging principles of osmosis, capillary wicking, and evaporation to collect and induce sweating without electronics. However, the suitability of this technique for reliable long-term monitoring remains unproven[35].

This work proposes a current-source architecture that seeks to overcome the limitations of prior art by simultaneously addressing the above challenges (**Fig. 4b**). In the schematic, U_2 is a traditional high-gain opamp, while U_3 is a current-sense amplifier that supports beyond-the-rail common-mode input voltage sensing and possesses a fixed voltage gain (A_v) of 20 V/V. The current source is programmed by changing the voltage on the opamp's positive input terminal, which is driven directly by the AD5940's 12-bit DAC output (V_{DAC}). Through the negative feedback operation of the amplifier control loop, V_{DAC} is copied to the output of the CSA, which allows for straightforward determination of the load current based on the CSA's gain and sense resistor (R_{sense}) value. The Miller compensation network, consisting of R_4 and C_2, is added to create a low-frequency pole in the system, ensuring feedback loop stability and setting the transient settling behavior of the current source.

Opamp U_2 supplies the stimulation current from a 20 V boost converter power supply. It features a rail-to-rail output stage, enabling an overall current source compliance voltage of just under 20 V. The stimulation current

then travels from the anode through the skin (denoted as resistor R_{load}) and returns to ground via the cathode. During a typical iontophoresis workflow, the skin interface can initially present a very large impedance (several 10s of kΩ) due to the absence of sweat. Notably, in these scenarios where the $I_{load} \times R_{load}$ product exceeds the compliance voltage (20 V), current flow will not be prevented (**Fig. 4l**). Rather, it is clamped to progressively lower magnitudes as impedance increases, implying that the sweat rate may be initially slowed but not inhibited altogether. As the sweat generation process begins to take effect, the skin impedance then settles to a lower value within the compliance range (<20 kΩ), allowing normal operation to resume (**Fig. 4j-k**). As such, a compliance voltage of 20 V is sufficient for practical operation, especially given recent research on carbachol-based iontophoresis[32]. Note that a lower compliance voltage can be set (resulting in reduced power consumption), if acceptable, by adjusting the boost converter output voltage. Assuming an R_{sense} value of 100 Ω and DAC voltages ranging from 0.2 to 2.0 V, this design provides a current range of 0.1 to 1 mA with a step size of ~268 nA. Changing the R_{sense} value adjusts the current range up or down by 20 dB, up to a maximum current of 1.5 mA, as set by the current limiter, providing flexibility for different applications or electrode patch form factors. Regarding the JFET current limiter, to test its clamping functionality, R_{sense} was assembled to a value of 50 Ω to artificially generate a scenario where the current source can be programmed into an overcurrent state (**Fig. 4m**). As intended, the current clamps to the JFET's ~1.5 mA saturation value, despite the DAC trying to force larger currents. Note that a low R_{load} value of 5 kΩ was used in this experiment to ensure the clamping is explicitly from the JFET, rather than from exceeding the 20 V compliance limit.

Several example current range and resolution combinations are tabulated in **Fig. 4b** using the current source's transfer function equation. The MCU's internal ADC monitors the delivered load current in real time for safety purposes via the CSA output voltage (a low-impedance node), with a passive first-order LPF for anti-aliasing. Note that during intended operation, the CSA output is bounded within the V_{DAC} output range (0.2 V – 2.0 V). Thus, it can be powered by a lower-voltage supply (3.6 V) instead of the 20 V boost converter (**Fig. 5a**), allowing the MCU to safely measure this node while rendering the CSA's quiescent current negligible. Critically, the proposed iontophoresis current source contributes just 35.5 mm^2 of additional area to the device (<7% of the total PCB area), including all components in the module and their associated routing. This module consumes a low quiescent current of 0.98 mA (since only U_2 is powered from the high voltage domain, while both the real-time monitoring and control voltage generation are accomplished in the low voltage domain), including the boost converter, while still meeting the programmability and safety requirements. A more thorough analysis of the iontophoresis current consumption is provided in **Fig. 6e,f**.

Supplementary Note 6. Potentiostat compliance voltage, CE clipping, and TIA gain tradeoffs/challenges.

In a TIA-based potentiostat, it is crucial to ensure that all electrode node voltages and the TIA output remain bounded within the system supply rails ($V_{DD,A}$ and $V_{SS,A}$) to prevent either amplifier from saturating (clipping). If clipping occurs at the CE or TIA output for a significant amount of time, the potentiostat's voltage regulation of the electrochemical cell ($V_{WE} - V_{RE}$) begins to fail, introducing strong measurement distortion and error. This challenge is depicted schematically in **Supplementary Fig. 8a**. A positive cell potential ($V_{WE} - V_{RE}$) is assumed for illustrative purposes. $\Delta V_{scan,max}$ refers to the maximum cell potential during a voltammetric or amperometric experiment. Additionally, the figure illustrates a Randles equivalent *RC* circuit that is commonly used to model the behavior of an electrochemical cell. In the circuit, R_{CT} models the charge-transfer resistance, which represents the faradaic signal component and is proportional to the target analyte concentration; C_{DL} models the capacitance at the interface between the WE and the electrolyte solution; and R_S models the resistance of the underlying biofluid/buffer solution. Similar *RC* parameters associated with the CE are also defined. Given this model and a positive $\Delta V_{scan,max}$, it is apparent from Ohm's Law that the CE will exist at a potential below V_{RE}. This potential difference between the RE and CE (ΔV_{CE}) is defined as the CE underpotential (or overpotential for negative $\Delta V_{scan,max}$). The potentiostat compliance voltage ($\Delta V_{Compliance}$) can then be defined as the potential between the WE and CE ($V_{WE} - V_{CE}$) and equals $\Delta V_{CE} + \Delta V_{scan,max}$. In the AD5940, the maximum supported compliance voltage is 2.4 V, set by the upper bound of the V_{WE} DAC output. Finally, since the sensor current (I_{in}) flows through the TIA feedback resistor (R_f), generating a voltage drop $\Delta V_{TIA} = I_{in} \times R_f$, a boundary condition for the potentiostat can now be established using this framework: $\Delta V_{Compliance} + \Delta V_{TIA} \leq V_{DD,A}$.

In wearable biochemical sensors, numerous scenarios require at least one of the following: 1) adopting pulsed voltammetry methods (*e.g.,* DPV or SWV) for enhanced sensitivity, 2) utilizing a large surface area WE for increased signal amplitude, 3) applying large TIA gain (R_f) for quantification of weak analyte concentrations, or 4) scanning up to large cell potentials (≥0.8 V) for identification of target analytes with large oxidation potentials. A few applicable examples include the detection of electroactive drugs for therapeutic drug monitoring or substance abuse monitoring[36–39], and the detection of various amino acids and nutrients for dietary health monitoring[32], although scenario 3 is common to almost all wearable applications. Each of these scenarios poses circuit-level challenges for the TIA-based potentiostat, making it difficult to simultaneously satisfy all the boundary conditions. In all four scenarios, large transient and/or static baseline voltages are developed that push the allowable limits on ΔV_{TIA} and ΔV_{CE}. For applications that fall into multiple (or all) of these scenarios, the challenges are greatly exacerbated. Pulsed voltammetry methods apply periodic pulses on the order of 10s of mV, generating instantaneous non-faradaic current pulses when passing through large C_{DL} and small R_s (≤ a few kΩ) values. The surface area of WEs in both sweat and interstitial fluid sensors can reach a few mm^2, as larger areas promote more redox reaction sites and larger signals, resulting in C_{DL}s ranging from ~1 to 100s of nF and subsequent current transients up to 10s of μA. When passed through an R_f in the 10s of kΩ range, these currents translate into instantaneous ΔV_{TIA} of 100s of mV. Moreover, if the faradaic analyte signal is weak (sub-μA or nA-levels), it may be required to increase the TIA gain (R_f) to reduce the circuit's input-referred noise, which can further increase ΔV_{TIA}[40]. These same transient currents flow through R_{CE} and C_{CE}, similarly increasing the CE underpotential. ΔV_{CE} is particularly affected when a target analyte requires a large $\Delta V_{scan,max}$ to fully resolve an oxidation peak or baseline signature, resulting in a larger baseline current flowing through the CE. Critically, predicting or controlling the value of ΔV_{CE} is challenging and highly application-dependent, as its value varies with the CE material properties, the CE geometry and area, the type of buffer solution, and the analyte concentration. Selecting a low-resistance CE material (*e.g.,* platinum instead of carbon in typical SPEs) with a larger area can help reduce R_{CE} and associated voltage drop; however, the challenge cannot be eliminated.

To illustrate these challenges schematically, a sequence of cases is presented in **Supplementary Fig. 8b-d**, using an example voltammetry scan with a maximum scan voltage of 1.0 V ($\Delta V_{scan,max}$ = 1.0 V). In case 1, consider that the AFE is operated at its minimum allowable supply voltage (2.8 V) and the WE common-mode (V_{WE}) is set to 1.3 V, the DAC's mid-scale value, establishing a compliance voltage of 1.3 V. At the scan's end, V_{RE} will equal 0.3 V, providing very little headroom for the CE to swing before clipping at $V_{SS,A}$. In practice, the CE will likely clip

well before the scan's end unless ΔV_{CE} has been hyper-optimized by the electrochemistry. Case 2 demonstrates a natural solution to this CE underpotential limitation, where the compliance voltage is increased to its maximum value of 2.4 V. ΔV_{CE} now has plenty of headroom (1.4 V) to avoid CE clipping; however, the clipping challenge has been moved to the TIA output, which only has 0.4 V of headroom before saturating at $V_{DD,A}$. Considering a capacitive transient spike of ≥10 μA on top of a baseline current, this limits the allowable TIA gain to an undesirably low value of at most 40 kΩ, presenting a major input-referred noise limitation for detecting small signals on top of this baseline. Case 3 solves both the CE and TIA clipping issues by increasing the AFE's supply voltage to its maximum (3.6 V). Note that the maximum compliance voltage of 2.4 V is maintained. Here, ΔV_{CE} can be as large as 1.4 V, while ΔV_{TIA} can approach 1.2 V. This effectively triples the allowable TIA gain compared to case 2, enabling a considerable reduction in input-referred noise and improvement to the sensor signal-to-noise ratio. Of course, this comes at the price of higher AFE power consumption, but it may be a necessary tradeoff in some applications. Although **Supplementary Fig. 8** illustrates cases with a $\Delta V_{scan,max}$ = 1.0 V, where a large compliance voltage is required, it is important to note that a larger supply voltage still provides substantial benefit even for lower $\Delta V_{scan,max}$ values. In applications with a reduced cell potential (*e.g.,* enzymatic glucose monitoring), a higher supply voltage allows the designer to fully maximize the TIA gain, pushing the system's sensitivity towards the potentiostat's theoretical minimum detectable signal. These considerations justify adopting a flexible power management architecture in the wearable device, which enables operating the AFE at any supply voltage within its allowable range (2.8 to 3.6 V) (**Fig. 5a**).

For added clarification, it is important to note that the AD5940 datasheet claims an ADC input common-mode range of 0.2 V – 2.1 V for any ADC MUX input that does not scale with $V_{DD,A}$. These claims, in principle, would limit the range of digitizable signals, and as such, portions of the node voltages illustrated in **Supplementary Fig. 8** may exceed this nominal range under high-compliance operating conditions. Nevertheless, during practical operation of the system, accurate current measurements were maintained under these conditions, providing empirical validation that the potentiostat signal chain can tolerate the stated high-compliance conditions. In this work, increasing $V_{DD,A}$ is therefore primarily leveraged to expand the available voltage headroom for the CE driver and TIA output and to prevent analog saturation, while the ADC input range remains a specified constraint for digitization. For pulsed voltammetry techniques in particular, this increased analog headroom allows the amplifiers to tolerate rapid double-layer capacitive currents without saturation, after which their output voltages decay back to lower values away from the supply rails prior to sampling/digitization.

Supplementary Note 7. Additional system power management considerations and justifications.

By far the most popular and simplest power management scheme in battery-powered devices is to directly down-regulate the battery voltage with an LDO, as they often possess small footprints, require minimal additional passive components, consume low quiescent current, and do not introduce supply voltage ripple. For a system that powers its AFE at 3.0 V or less, for example, this is an acceptable solution since most LDOs can function with <100 mV of dropout voltage, allowing the electronic system to remain functional even as the battery discharges down to ~3.1 V. Therefore, the vast majority of the battery's usable operating range is leveraged. However, considering the potentiostat compliance challenge (**Supplementary Note 6**), an LDO supply voltage ($V_{DD,A}$) of 3.6 V for the AFE would reduce the usable battery range by approximately half (from 3.0–4.2 V to 3.7–4.2 V), thereby dramatically decreasing the device's battery lifetime. This realization forms the basis for the adoption of a buck-boost converter following the battery in this work, as it can generate a stable output for the system regardless of the battery's voltage (**Fig. 5a**). Fortunately, the area overhead of the converter is tolerable, occupying 41 mm^2 (<8 % of the total PCB), including the layout and routing of all associated passive components. Additionally, a power diode (PMEG3010ER,115, Nexperia), located on Biocoin (**Fig. 5a**), allows the USB voltage (V_{USB}) to take over, providing power to the device during a charging scenario when it is connected to the docking station. The diode's low forward drop ensures that the input to the buck-boost converter remains within its acceptable input range, even as the battery voltage decays to 3.0 V.

Before device fabrication, evaluation modules of the buck-boost converter and AD5940 were used for prototyping to assess the impact of supply ripple on the analog circuitry. An undesirably large output voltage ripple of up to 50 mV_{pp} was observed from the buck-boost converter, which further coupled into the potentiostat front-end (WE and RE nodes). To eliminate the possibility of these switching transients disturbing the electrochemical cell, an LDO was added to filter the ripple via its power supply rejection ratio (PSRR). When powering the AFE at 3.6 V, this cascaded regulator scheme indeed introduces an extra boost conversion step, causing an additional current draw from the battery (relative to an LDO-only scheme). For $V_{DD,A}$ = 3.6 V, the buck-boost converter CH1 outputs 4.1 V (boosting the battery from a nominal 3.7 V), providing a 500 mV dropout for the LDO (**Fig. 5a**). Ideally, a smaller dropout would have been utilized to save power; however, the converter features discrete (resistor-tunable) output settings and 4.1 V was the closest option >3.6 V. Note that the MCU's maximum allowable supply voltage is also 3.6 V, which prevents using the same buck-boost channel that powers the LDO from also supplying the MCU. This represents one of the benefits of a multi-channel buck-boost solution, as it avoids the need for additional regulators. The selected LDO (TPS7A2036, Texas Instruments) provides strong PSRR, consumes 6.5 µA of quiescent current, and contributes negligibly to the total PCB area (1 mm^2). Nevertheless, despite this power penalty, we believe that this is a worthwhile tradeoff, as it simultaneously enables maximum potentiostat compliance while removing the supply ripple. Given that in many applications, the analog blocks will predominantly operate in a low-power hibernate state or will be disabled entirely (including the LDO) in deep sleep mode, the power penalty is tolerable, especially since the full battery range is always leveraged.

Although adding the LDO removed any significant ripple present on the AD5940's supply voltage, it is also important to note that the AD5940 is a mixed-signal IC and contains both sensitive analog circuitry as well as its own digital backend with dedicated supply pins for both domains, as seen in **Supplementary Fig. 22.** This introduces the possibility for switching transients, digital noise, or other artefacts (*e.g.,* during SPI configurations, FIFO reads, *etc.*) to couple back into the potentiostat via the supply pins and cause measurement errors. Rather than introducing yet another voltage regulator to separate these rails, which would add further area, quiescent power, and cost to the PCB, a ferrite choke was added in between the AD5940's analog and digital supply pins (specifically between pins A4/F2 and F3/G2). The ferrite presents low DC resistance while providing high impedance at switching frequencies, effectively forming an *LC* low-pass filter with local decoupling capacitors and preventing digital noise from propagating into the AD5940's analog supply domain. For similar reasons, the PCB's analog and digital grounds ($V_{SS,A}$ and $V_{SS,D}$) were isolated and joined at a single point via a ferrite choke. The single-point connection strategy is a standard good practice in any mixed-signal design as it ties all ground connections to the same DC potential, but eliminates undesirable ground loops between the analog and digital

domains, thereby confining any digital current return paths away from the sensitive analog regions. Both ferrites are annotated for reference in **Supplementary Fig. 20**. Dedicated power/ground planes and physical separation between analog, digital, and power circuitry were also employed to enhance isolation and signal fidelity.

However, it is recognized that not all biosensing applications require large potential scan ranges or push the limits of the potentiostat's input-referred noise and thus do not require enhanced compliance. To that end, Biocoin was also assembled and characterized with a $V_{DD,A}$ = 3.0 V configuration to notably remove the aforementioned buck-boost power penalty. Furthermore, for AFE operation below 3.6 V, a different LDO can be utilized with the same footprint (TPS7A0230, Texas Instruments), dropping its quiescent current to a negligible 25 nA. Accordingly, CH1 of the buck-boost now outputs 3.3 V to feed the LDO, and CH2 and CH3 were reduced to 3.0 V to match the AFE's supply. In this scheme, the power consumption during sampled measurement techniques is significantly reduced (**Fig. 6** and **Supplementary Table 6**), even lower than that of a theoretical LDO-only architecture. Note that the employed LDOs in either $V_{DD,A}$ configuration are available in numerous other output voltage settings, ranging from 2.8 to 3.6 V, according to the application's needs. The component assembly BoMs for both the $V_{DD,A}$ = 3.6 and 3.0 V configurations are provided in **Supplementary Tables 7 and 8**, respectively.

Regarding the iontophoresis boost converter selection, it is essential to recognize that the majority of commercially available high-voltage converters are optimized for efficiency under large load scenarios (*e.g.,* 10s to 100s of mA loads or greater). As such, they often utilize physically large components (inductors, power diodes, capacitors) to meet these energy requirements and consume large quiescent currents. However, when used in wearable applications that operate with a load current of < a few mA (*e.g.,* iontophoresis), boost converter efficiencies degrade significantly, resulting in dramatically amplified current draws from the battery. In particular, the LMR64010 (Texas Instruments) has historically been the most popular boost converter among prior art iontophoresis wearables, but it suffers from an efficiency of < 25%, resulting in tens of mA of current consumption[26,27,32,33]. Fortunately, the growing prevalence of wearable devices has prompted the development of newer boost converter solutions optimized for light load scenarios, such as the MAX17291 (Analog Devices) and the TPS61096A (Texas Instruments). These solutions also provide power diode integration for reduced area. The MAX17291 was adopted in this work, providing an efficiency of over 80% in the expected sub-mA stimulation range with a current draw of just 28 µA. These improvements allowed for a compact total boost converter layout of 22 mm^2, notably smaller than charge-pump-based approaches that operated at the same 20 V compliance[28].

Finally, not all wearable biochemical applications require sweat extraction via iontophoresis (*e.g.,* many microneedle applications that sample interstitial fluid). In these scenarios, the variable current source can be used for additional functionality. Two such applications include drug delivery into the skin (still via iontophoresis, but with different positively charged drugs loaded onto the anode) and reverse iontophoresis for the extraction of biomarkers in interstitial fluid (an alternative to microneedles). In both cases, the current source still operates as described in this work. Other possible current source functionalities include 1) pulsed (alternating current (AC)) current injection for electrical stimulation therapy and/or controlled drug delivery, or 2) acting as a heater module where electrical current flowing through a thermally conductive material generates heat. In particular, the current source acting as a heater module can be combined with a resistive temperature sensor, where both the thermal element and the temperature sensing element are located on a wearable patch, to create a closed-loop temperature control system for electrochemical reactions. Temperature readings via the AD5940 could be used to adjust either the current value or the duty cycle (pulse width modulation) of the current waveform supplied to the heating element, thereby controlling the temperature. If the sense resistor's (R_{sense}) value in the current source was accordingly decreased and the current limiter was bypassed (**Fig. 4b** and **Supplementary Fig. 23**), the control opamp can source up to 80 mA of load current for this application. Similarly, a drug delivery module coupled with a multiplexed biosensor could enable a closed-loop therapeutic drug monitoring system. In either the temperature control or AC stimulation scenarios, likely, a large 20 V compliance voltage is not required for operation. To that end, a set of 0 Ω jumper footprints was also placed on the PCB to optionally power the opamp in the current source from the same lower voltage buck-boost channel as the CSA (**Fig. 5a** and **Supplementary Fig. 23**).

Supplementary Note 8. Electronic system modes of operation.

Modes of operation. When used in a practical wearable application, Biocoin may need to perform a wide range of tasks, including various electrochemical measurements, BLE radio communication, iontophoresis, data processing, and battery monitoring. Multiplexed measurements and/or periodic task sequences may also be performed. In most applications, the system spends most of its lifetime in a sleep or idle state, where the device operates between periodic measurement sampling events or awaits further BLE instructions from a host device. Consider the following sequence of events, which illustrates a typical application. First, a host device sends measurement instructions over BLE to Biocoin, which the MCU decodes. The MCU then powers on the appropriate analog blocks and programs/configures the AD5940 accordingly, based on the received instructions. The AFE then begins the measurement, collecting data at a pre-defined sampling interval. When data is obtained, the MCU reads and processes it, then immediately queues the data for BLE transmission back to the host device during the next available connection interval. Between sampling or data-read events, the MCU and most of the AFE's internal blocks are put into sleep states to reduce their idle power consumption. As such, the device's average power consumption can be heavily optimized based on the frequency of data sampling and BLE communication events. Slower sampling rates and longer BLE connection intervals reduce average power, at the expense of reduced temporal resolution and increased latency.

To encapsulate the various functions executed by Biocoin and provide an organized way to report power consumption, we distinguish between tasks, states, and modes of operation in this manuscript. The distinction is also illustrated graphically in **Supplementary Fig. 17a**. Tasks refer to individual or instantaneous events. Some examples include, but are not limited to, an ADC or BLE radio turning on/off, the iontophoresis current source turning on/off, the MCU going to sleep or waking up, the flashing of the on-board status light emitting diode (LED), *etc*. Simply put, tasks transition the device between different states. States represent the device's continued operation after a specific task has been executed. A few state examples include running constant-current iontophoresis, receiving or transmitting a BLE packet, sampling data with an ADC, the MCU reading or processing data, leaving the low-power potentiostat biasing on between amperometry samples, or the MCU remaining asleep for a specified period. As such, the average power consumption of a state does not include multiple transitional tasks. Modes of operation, on the other hand, refer to sequences or combinations of multiple states and tasks that collectively encompass all functionality required for wearable operation over a longer period. The average power of a mode, therefore, represents the average of all tasks and states that comprise the mode. Using iontophoresis as another example, the average power during current delivery refers to the power required to continuously maintain the current source in operation. The average power of the iontophoresis mode, however, also includes the power required for periodic current monitoring, BLE communication, and MCU housekeeping tasks (such as battery monitoring and status LED flashing).

Three modes of operation are defined in this work, as outlined below, each comprising several tasks and states that are either continuously or periodically executed during the mode. **Supplementary Fig. 17b** tabulates which circuit blocks are enabled or disabled during each mode of operation.

<u>Mode 1: Deep Sleep</u> – The lowest average power consumption mode available on the wearable device. In this mode, all analog measurement blocks and iontophoresis blocks are completely shut down. Only the battery monitor circuit, one output channel (CH3) of the buck-boost converter, and the MCU are powered on. Although the MCU is on, it primarily operates in a low-power 'idle' sleep state, switching to BLE communication or housekeeping tasks only periodically.

<u>Mode 2: Active</u> – This mode refers to when analog measurements are being conducted (including any of the electrochemical methods, impedance analysis, or resistive sensor measurements). As a result, this mode can be subdivided into categories, each with a different average power consumption depending on which measurement method is active at a given time. The battery monitor, CH3 of the buck-boost converter, and the MCU are still on; however, CH1 of the buck-boost converter is now also enabled, which powers the downstream

LDO and analog blocks. Since the AD5940, voltage buffers, and 4:1 MUX are all powered via the same load switch, all are turned on together for any electrochemical method. The resistive voltage dividers are powered via a separate load switch; thus, that block will only be enabled if it is specifically being measured. While an active measurement sequence is executed, the AD5940 alternates between two states: 1) ADC sampling when data needs to be obtained and 2) a low-power hibernate state. During hibernation, all blocks within the AD5940 are powered down internally, except for the low-power (LP) potentiostat loop (comprising two LP amplifiers, an LP bandgap reference, an LP reference buffer, and an LP DAC), the low-frequency 32 kHz oscillator, and the wakeup timer logic. This allows for electrochemical cell biasing and timing control to be maintained at all times, with minimal power consumption, in between sampling events. The MCU alternates between the same states as detailed for the deep sleep mode, plus additional states for reading and processing data from the AFE.

Mode 3: Iontophoresis – In this mode, all iontophoresis blocks are powered on, including CH2 of the buck-boost converter, the boost converter, the control opamp, and the CSA. Since the current source's input control voltage is provided by the DAC within the AD5940, all analog measurement blocks are also powered on, except for the resistive voltage dividers. However, unlike the active mode, the AD5940 can remain in its hibernate state during iontophoresis, as it does not need to perform sampling operations. The same always-on blocks as described in deep sleep mode are also powered on, except that the MCU now alternates between four states: idle, BLE communication, housekeeping jobs, and periodic overcurrent safety monitoring.

Supplementary Note 9. High-frequency sampling operation, BLE throughput, and worst-case system power consumption.

The maximum achievable sampling rate of the Biocoin platform is jointly limited by the AFE signal chain and the wireless data transmission configuration, including the BLE connection interval, slave latency, AFE filter settings, and the processing interval. As described in the main text, the configuration adopted in this work represents a practical operating point that balances power consumption and latency for most wearable sensing applications. To be more specific, the effective maximum sampling rate is also governed by the BLE link's ability to reliably transmit acquired data without packet loss. In this work, the maximum supported sampling rates are empirically defined as the highest rates at which the number of received samples matches the expected count based on the programmed sampling interval and the experiment duration. Sampling beyond this limit can result in dropped packets due to communication bottlenecks. Alternative configurations may be employed to further increase the sampling rate or reduce latency, depending on application-specific requirements; however, in what follows, the system's limits are characterized based on the current BLE framework reported herein. Three data acquisition and transmission strategies were evaluated, each introducing tradeoffs between sampling rate, latency, and power consumption:

1. Buffered transmission – With this scheme, the processing interval parameter is leveraged to locally store data inside the AFE's FIFO before it is then read all at once by the MCU and transmitted over BLE in batches. This approach reduces BLE duty cycle and power consumption but increases latency. As described in **Methods**, using a processing interval set to 10× the sampling interval yielded a favorable power-latency trade-off and was subsequently used for measurements in **Fig. 6** and **Supplementary Table 6**. The latency with this scheme, therefore, equals the processing interval.

2. Real-time streaming – Here, the processing interval is set equal to the sampling interval, and each sample is transmitted immediately for point-wise visualization. This represents the worst-case condition in terms of power consumption, in exchange for minimized latency (in this scheme, latency simply equals the sampling interval).

3. Post-acquisition FIFO readout – Alternatively, samples can be stored locally in the AD5940 FIFO (1024 samples maximum) during acquisition and transmitted only after completion of an entire measurement (processing interval is set equal to the measurement duration). This eliminates communication overhead during sampling and enables higher effective sampling rates, but is limited in practice to shorter duration measurement techniques (*e.g.,* voltammetry methods) due to the AFE's finite FIFO depth. Here, latency therefore equals the measurement duration.

For both the buffered (10×) and real-time (1×) transmission strategies, the minimum supported sampling interval where packet loss is not introduced is ~3 ms, as reported in **Supplementary Table 3**. This corresponds to sampling rates of 333 Hz for amperometry (and other time-based readout methods) and 167 Hz for SWV or DPV, since these methods acquire two samples per square wave period. At this sampling rate, the average "worst-case" current consumption is 2.11 mA for buffered transmission (52.1-hour battery lifetime) and 3.70 mA for real-time streaming (29.7-hour battery lifetime). Note that this 3 ms minimum sampling interval limit is approximately the same for both the buffered and real-time strategies because BLE transmission time is dominated by radio startup and protocol overhead for small overall payload sizes, such that transmitting multiple samples per packet introduces minimal additional delay.

Using the post-acquisition FIFO strategy, higher sampling rates of up to 600 Hz (amperometry) and 300 Hz (SWV) were achieved without packet loss, with an average "worst-case" current consumption of 3.37 mA (32.6-hour battery lifetime). This approach is well-suited for voltammetric techniques with finite scan durations. While it does not provide point-by-point real-time streaming, in many practical applications, the primary output of interest to the wearable's end-user or patient is simply the extracted biomarker concentration values from each voltammogram (*e.g.,* peak current or potential), rather than the full raw waveform.

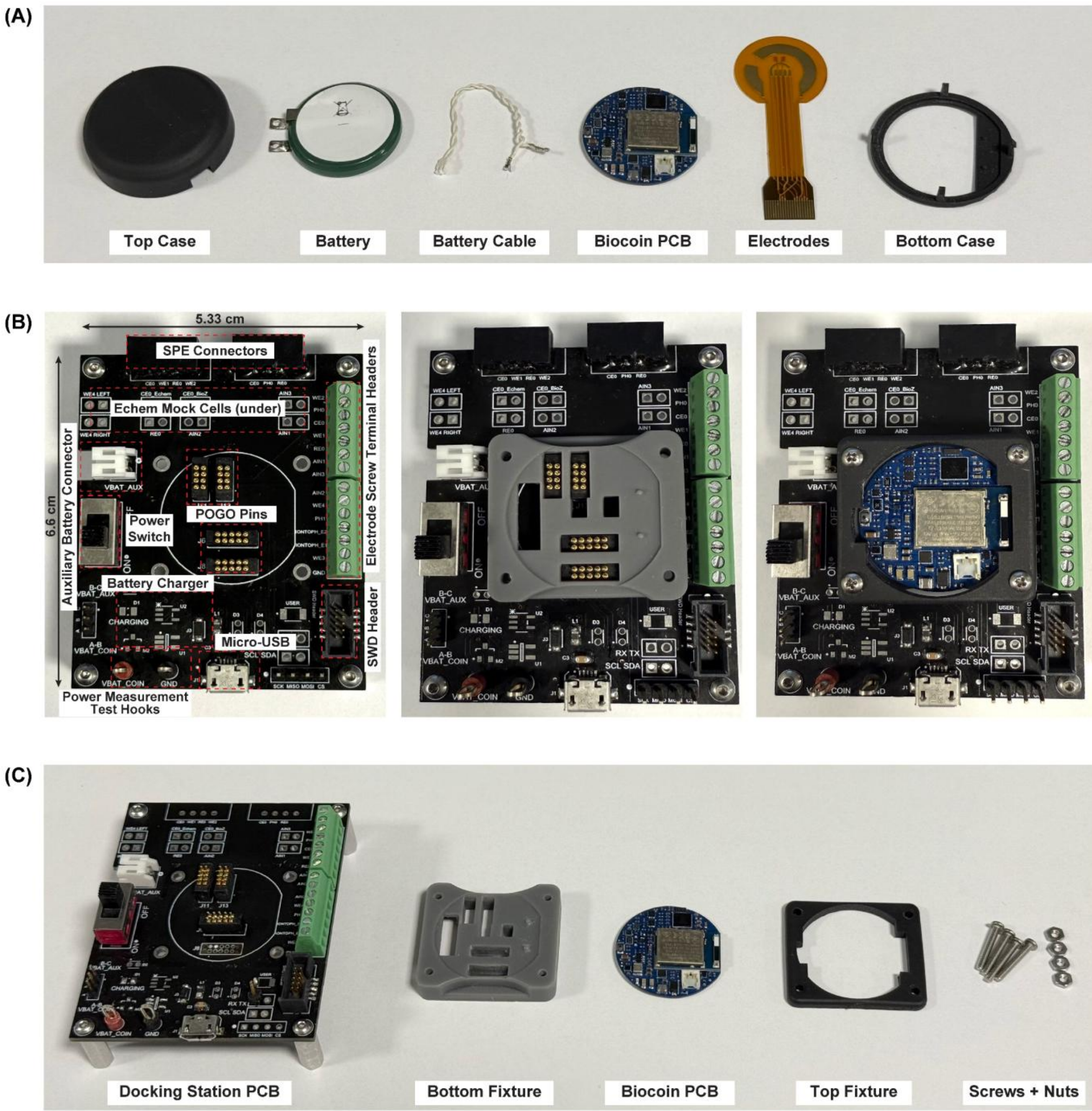

Supplementary Figure 1. Photographs of the Biocoin and docking station subcomponents. (A) Photograph of the sensor device's disassembled components that are employed for wearable operation. **(B)** Annotated photograph of the docking station PCB (left). Photograph of the bottom fixture piece assembled into the docking station via press-fitting (middle). Photograph of the fully assembled docking station system, including Biocoin, secured together via screws (right). **(C)** Photograph of the docking station's disassembled components that enable firmware programming, device characterization, and operation as a portable potentiostat module.

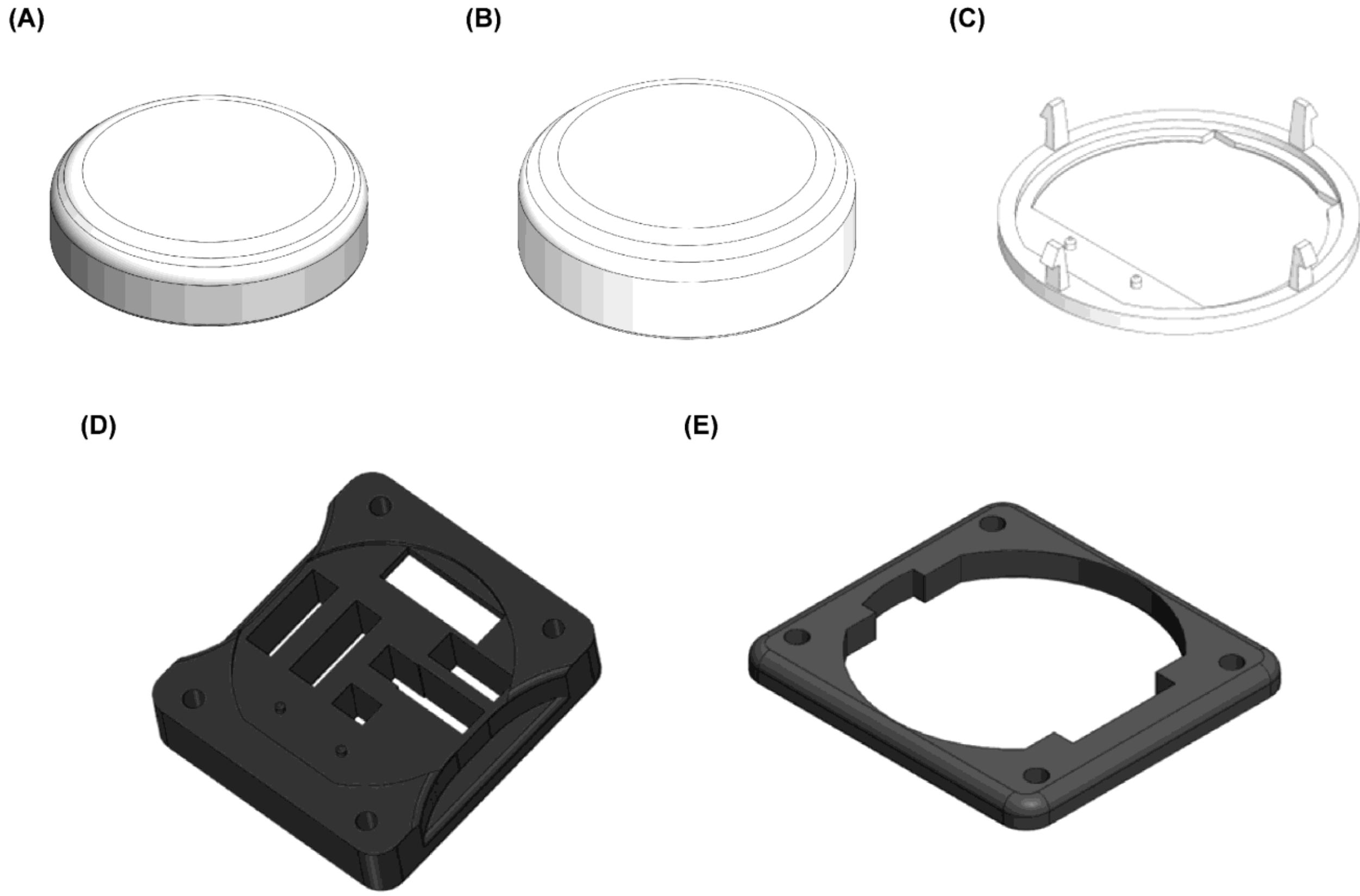

Supplementary Figure 2. 3D CAD model images of the 3D-printed fixture components. (A) Biocoin top case (7.366 mm height variant). **(B)** Biocoin top case (9.525 mm height variant). **(C)** Biocoin bottom case. **(D)** Docking station bottom fixture. **(E)** Docking station top fixture.

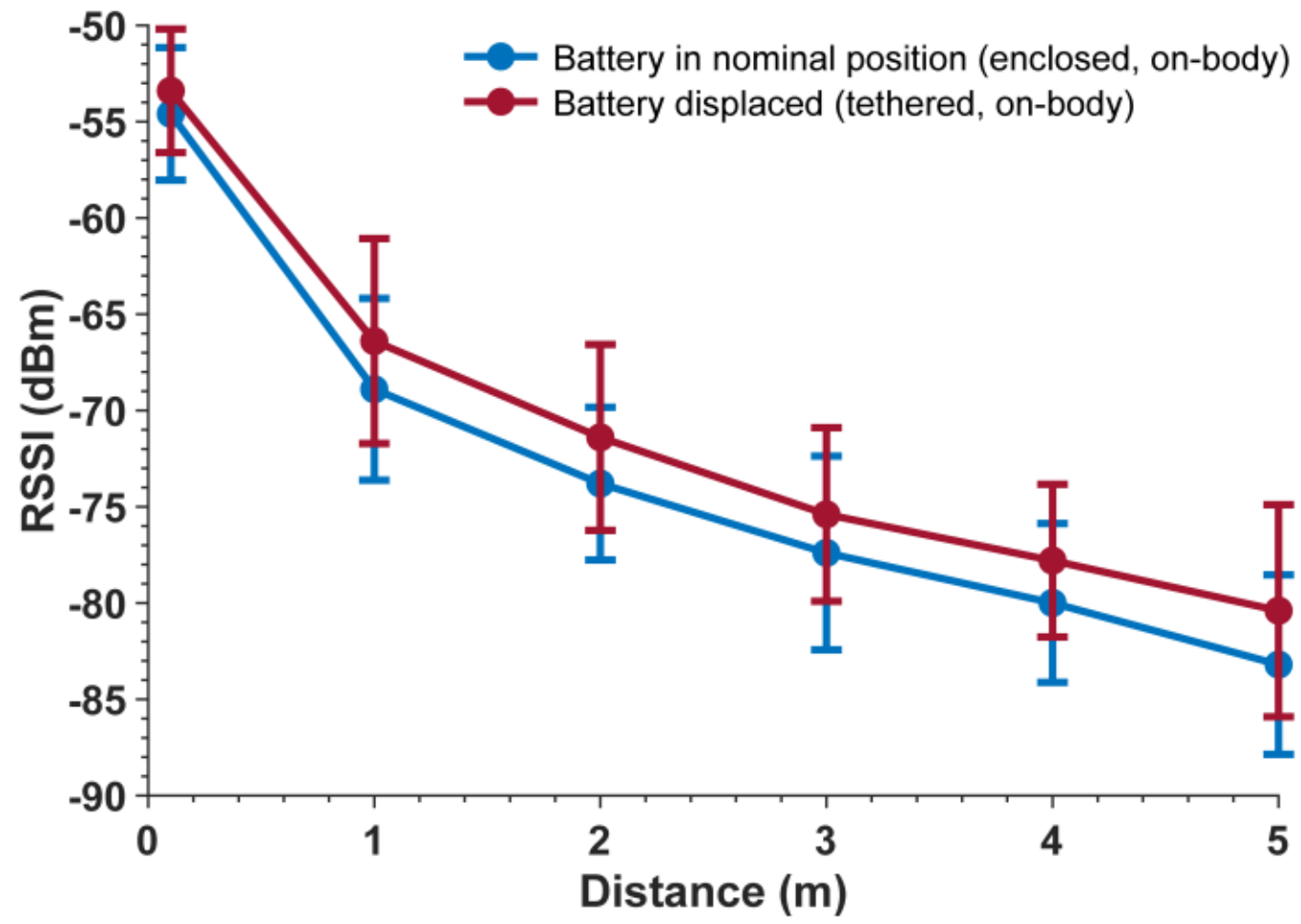

Supplementary Figure 3. BLE RSSI vs. distance with and without battery placement. Measured received signal strength indicator (RSSI) as a function of distance for the Biocoin platform with the battery in its nominal position above the BLE module and with the battery displaced. In the nominal case, the fully assembled wearable (enclosed device) was adhered to the arm; in the displaced case, the battery was removed from the enclosure and tethered off to the side while the PCB remained mounted on the arm. Measurements were performed using the nRF Connect mobile application by recording RSSI from BLE advertising packets at fixed distances in an indoor environment. At each distance, *n* = 5 readings were averaged; error bars represent one standard deviation.

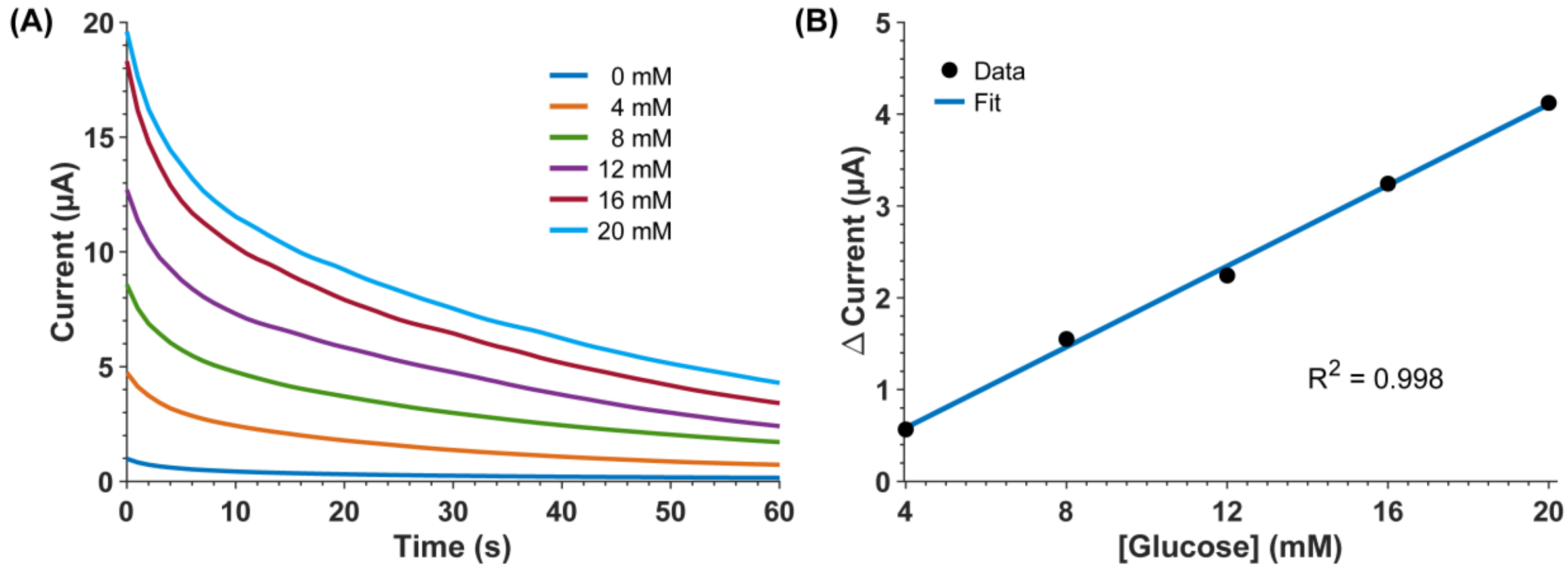

Supplementary Figure 4. Amperometric glucose sensing in artificial interstitial fluid (ISF). Amperometric glucose measurements performed using a commercial glucose oxidase enzyme-based test strip (Blood Glucose Test Strips, G-425-2, Lvonge) interfaced with the Biocoin platform in artificial ISF. The ISF solution was prepared according to a previously reported formulation[41–43] using reagent-grade chemicals (*e.g.,* glucose, salts, buffers) purchased from Sigma-Aldrich. **(A)** Measured current transients following application of a 0.6 V fixed potential for glucose concentrations ranging from 0 to 20 mM (covering the physiological range of glucose in ISF). **(B)** Corresponding calibration curve extracted from Time = 60 s, demonstrating a linear response (R^2 = 0.998) across the measured concentrations. These results illustrate the platform's compatibility with biorecognition element-based electrochemical sensors for biomarker detection.

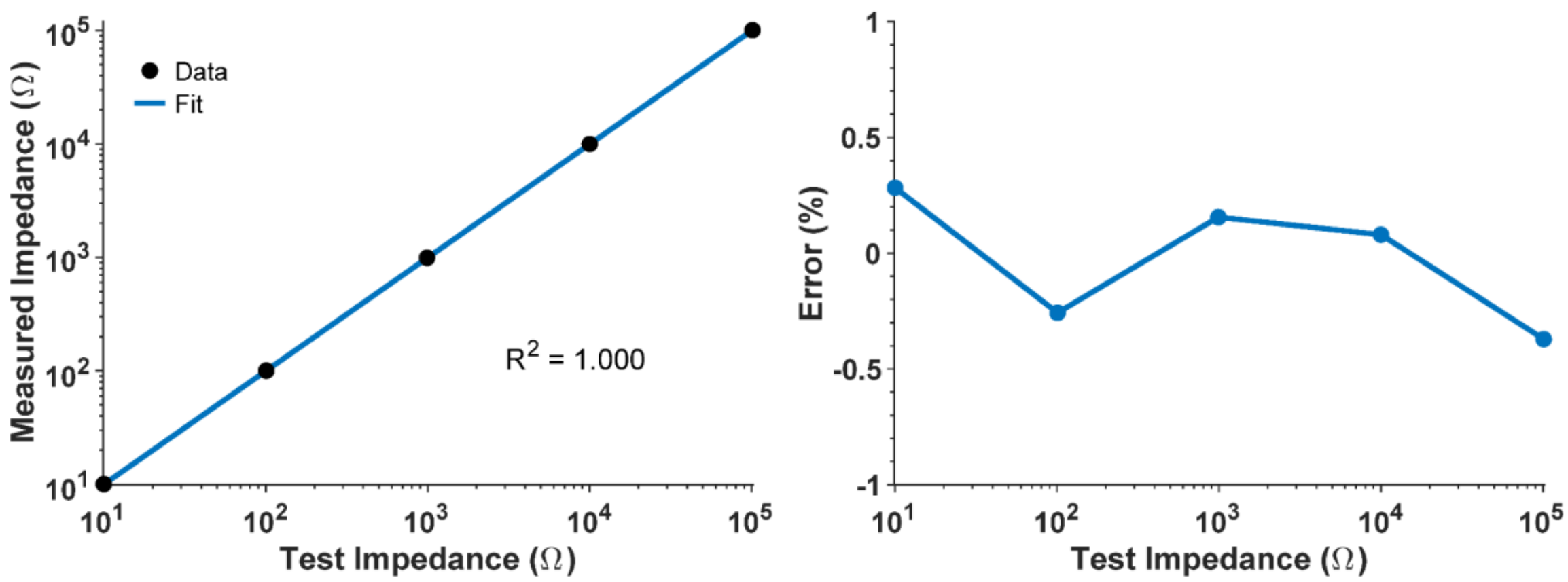

Supplementary Figure 5. Measurement results of the impedance sensing engine. (A) Measured impedance curve in response to applied load resistors of known values. **(B)** Corresponding error plot between the measured vs. applied load resistances. Error expressed in %.

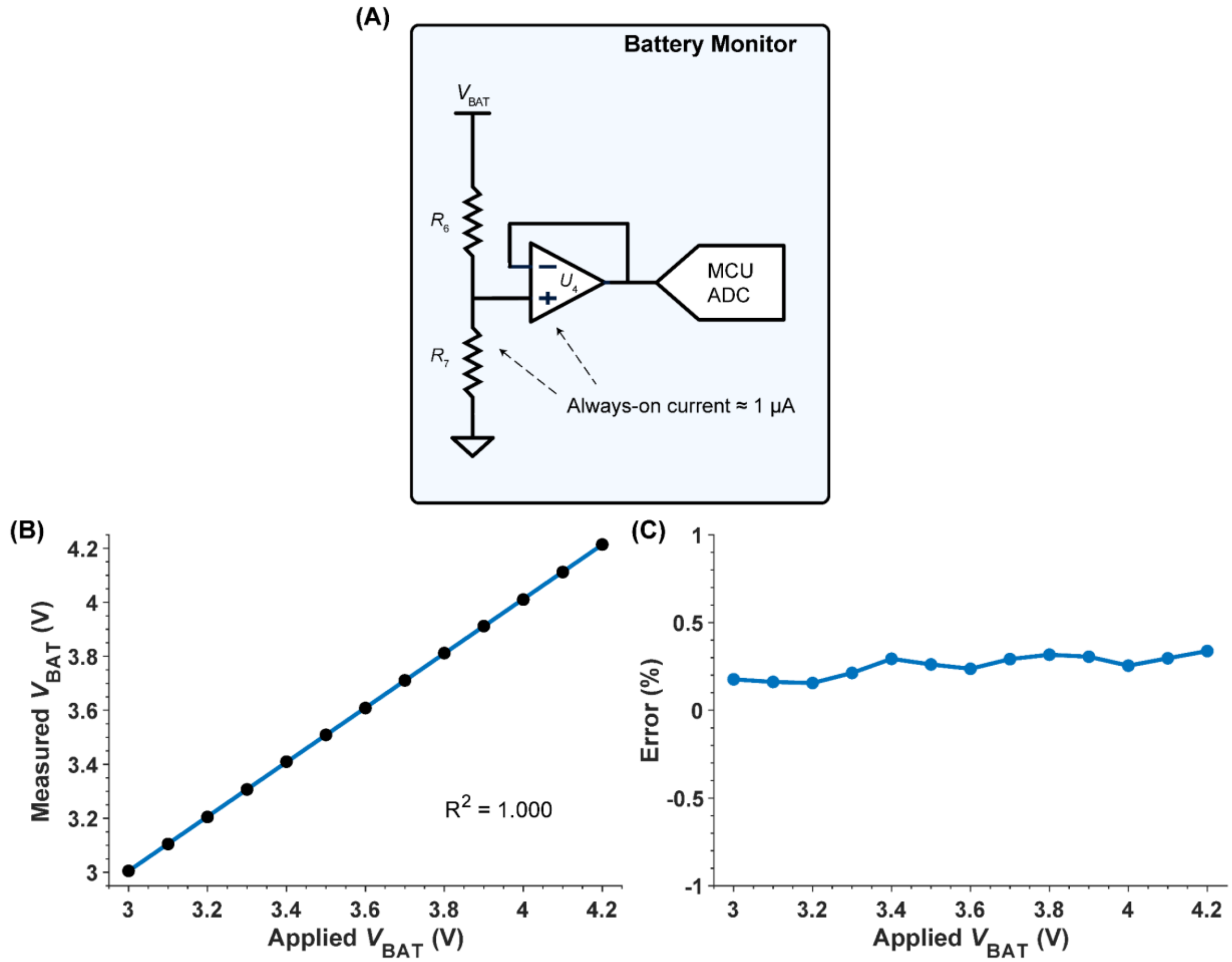

Supplementary Figure 6. Schematic and measurement results of the battery voltage monitor. (A) Schematic of the resistive voltage divider and opamp buffer circuitry. **(B)** Measured DC transfer curve in response to applied benchtop input potentials (V_{BAT}) across the usable voltage range of a Li-Ion battery. **(C)** Corresponding voltage error between the measured vs. applied input voltages. Error expressed in %.

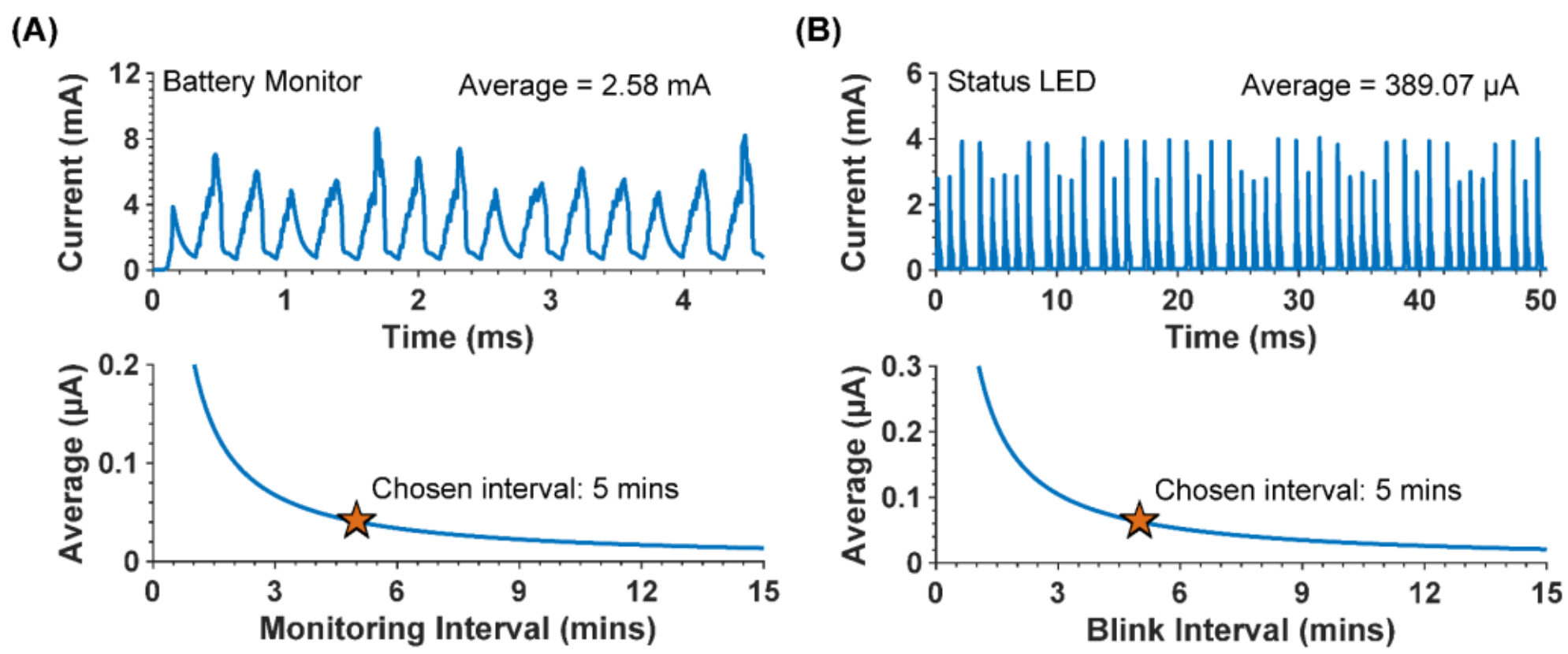

Supplementary Figure 7. Current consumption of the battery monitoring and status LED housekeeping operations. (A) Measured instantaneous battery monitoring current profile when the voltage is measured by the MCU's ADC (top). The ADC measurement process takes ~4.6 ms. Calculated average current consumption as the time interval between ADC reads is increased (bottom). This average current adds to the ~1 µA of always-on current reported in **Supplementary Fig. 6a** for the battery monitor's analog components. **(B)** Measured instantaneous current profile of the status LED when blinked for 50 ms (top). Calculated average current consumption as the time interval between LED flashes is increased (bottom).

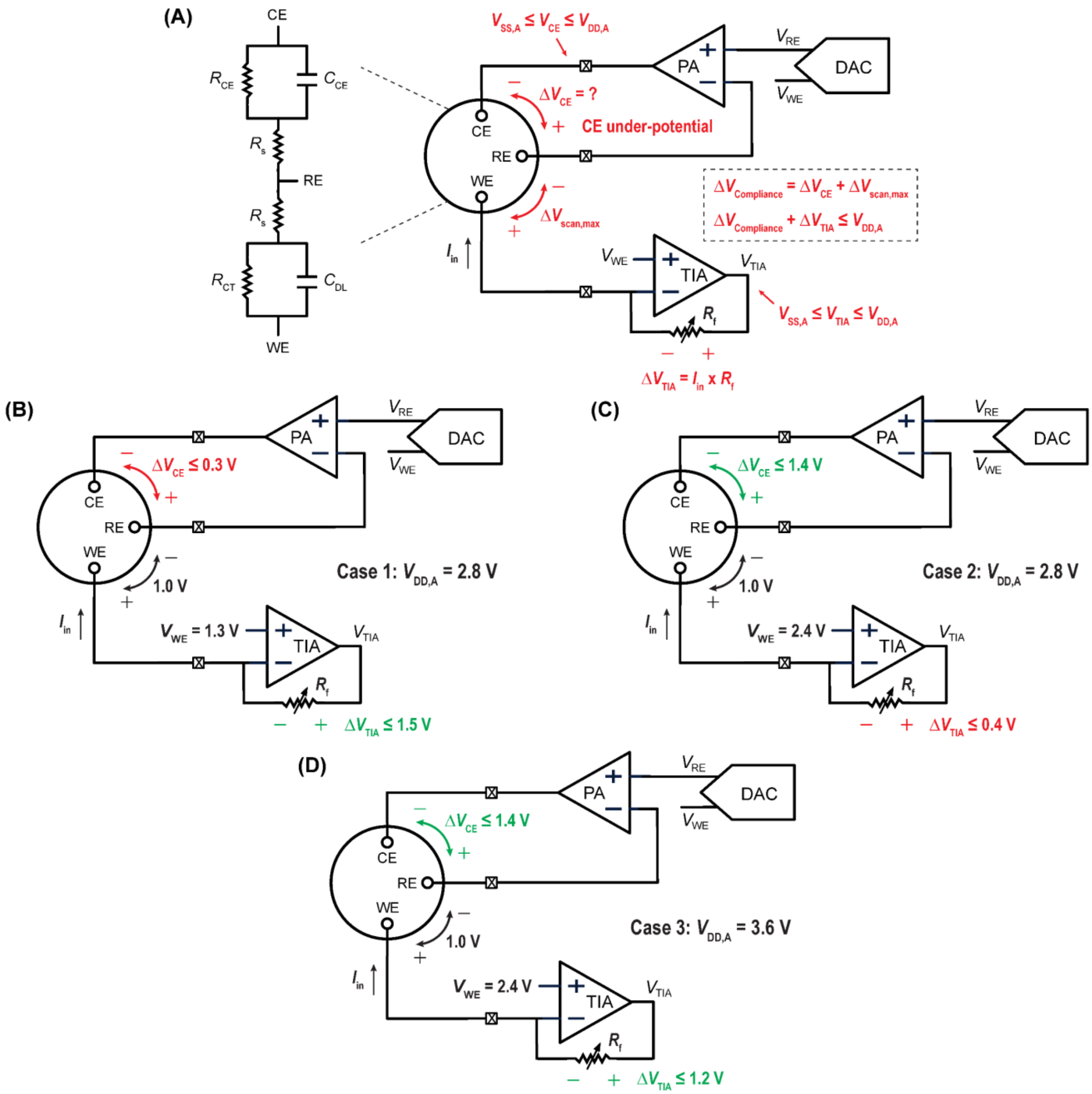

Supplementary Figure 8. Schematic illustration and examples highlighting the challenges in a TIA-based potentiostat regarding compliance voltage, TIA gain, and amplifier clipping. (A) Annotated schematic of the AD5940's TIA-based potentiostat defining various node voltage boundary limitations alongside a Randles circuit model of an electrochemical cell. **(B)** Example case of limited CE voltage headroom due to a mid-scale potentiostat compliance setting and a minimum AFE supply voltage. **(C)** Example case of limited TIA voltage headroom due to a maximum potentiostat compliance setting and a minimum AFE supply voltage. **(D)** Example case of improved CE and TIA voltage headroom due to a maximized AFE supply voltage, while maintaining the maximum compliance setting.

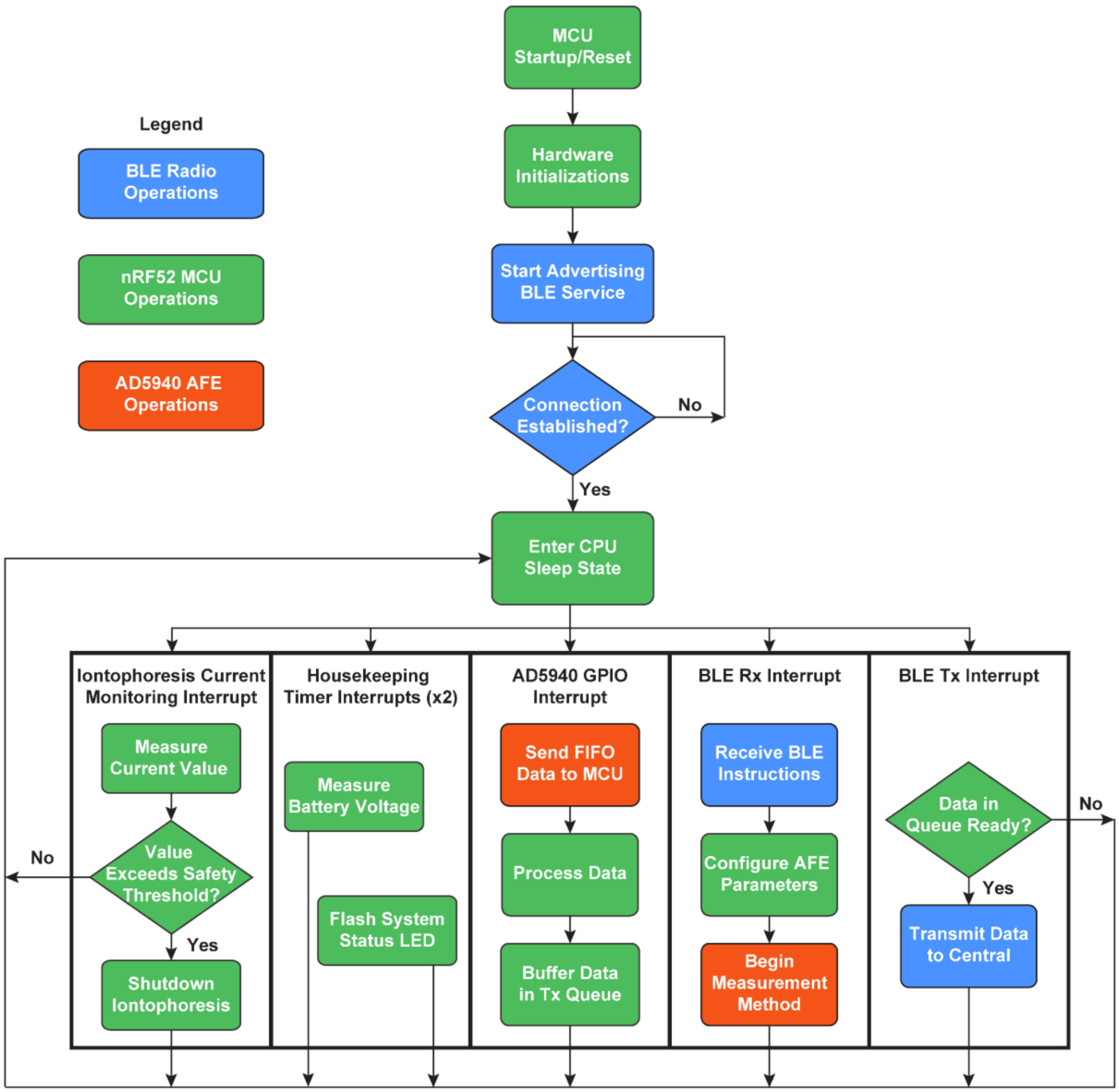

Supplementary Figure 9. Flow chart of the wearable device's firmware operation. The flow chart illustrates a simplified view of the MCU's interrupt service routine-based operation allowing the CPU to remain asleep during the majority of the device's battery lifetime. Operations are color-coded according to whether they are executed by the MCU, BLE radio, or the AD5940.

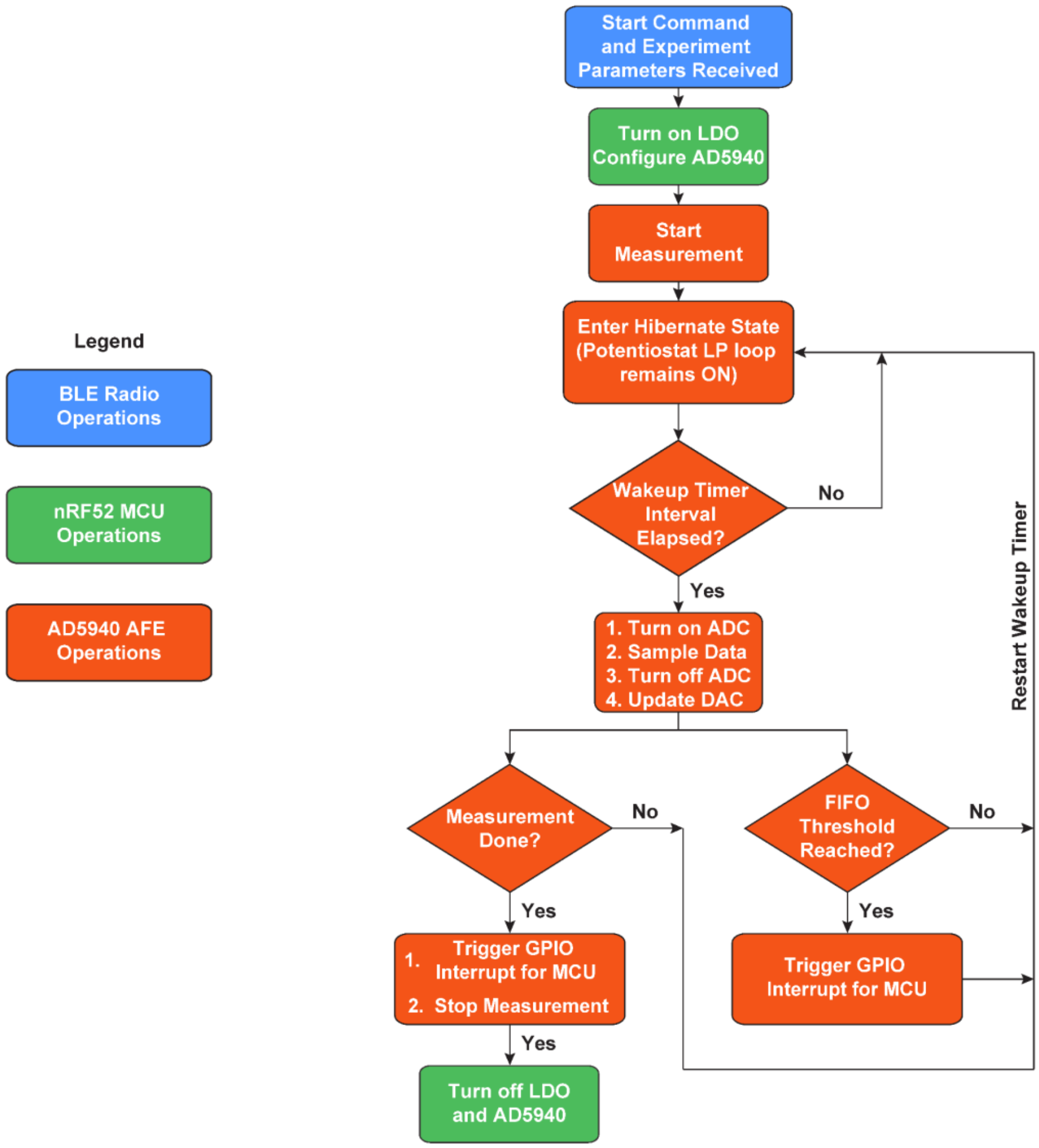

Supplementary Figure 10. Firmware flow chart specific to the AD5940's operation during measurement routines. The flow chart illustrates the general operation of the AD5940, whose sequencer, data FIFO, wakeup timers, and GPIO interrupt generation allow the chip to remain in a low-power hibernate state for the majority of the time during an electrochemical, resistive sensor, or impedance measurement routine.

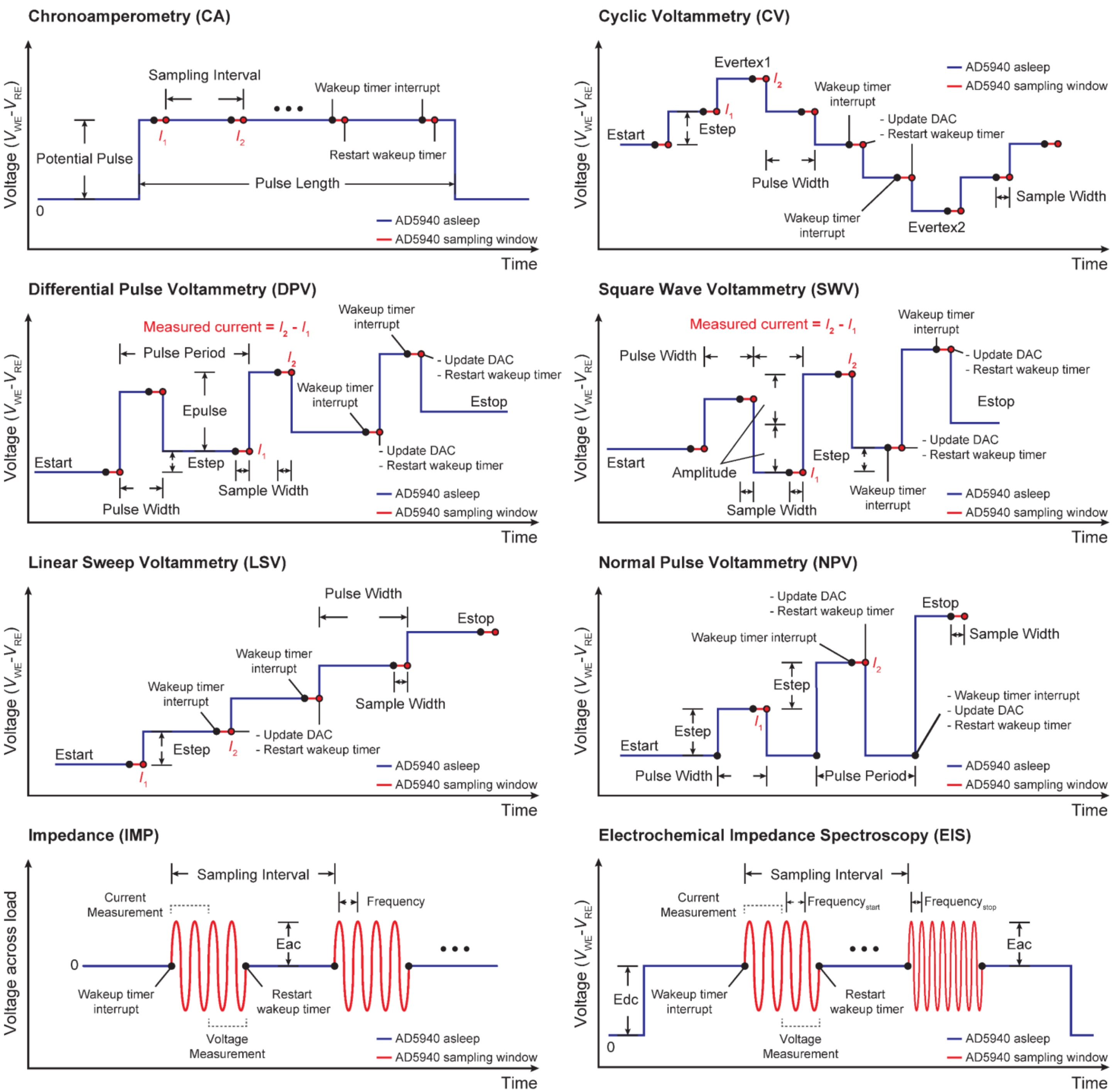

Supplementary Figure 11. Implementation diagrams of the different electrochemical and impedance measurement methods supported by Biocoin. Waveforms for each measurement method are depicted, accompanied by annotations for all associated voltage or timing parameters. The AD5940 sequencer is programmed prior to the start of a given method, allowing the AD5940 to remain asleep the majority of the time and only wake up during sampling windows. Note that OCP and resistive sensor measurements (*e.g.,* temperature measurements) operate similarly to the depiction of CA, but are not drawn as they do not apply an explicit waveform. The waveform parameters for all methods are exposed to the user in the firmware and can be programmed via the custom Python control script or a mobile application.

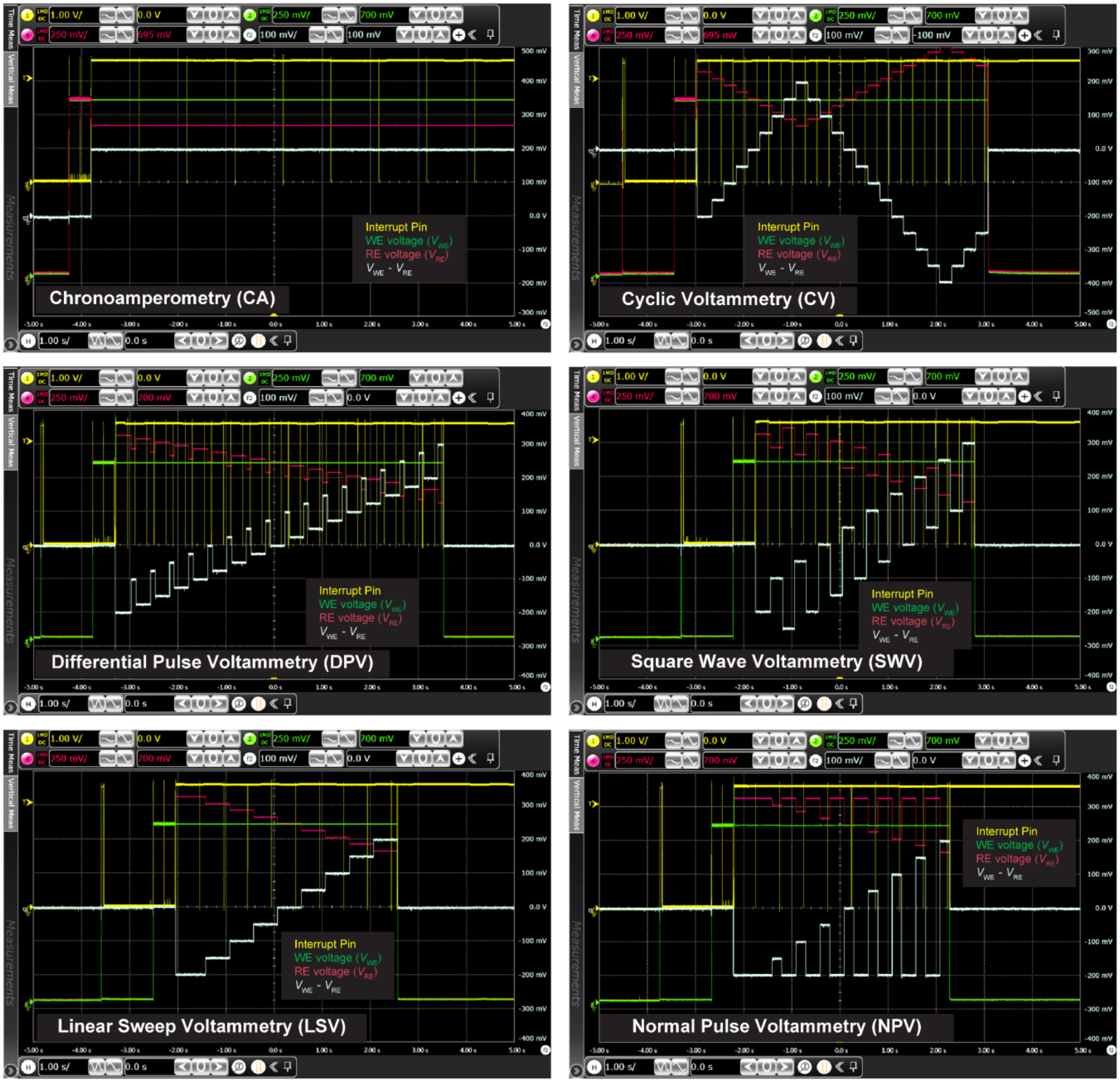

Supplementary Figure 12. Example waveform oscilloscope captures of the six core electrochemical methods: CA, CV, DPV, SWV, LSV, and NPV. Each image displays the individual WE and RE voltages with respect to ground (V_{WE} and V_{RE}, respectively), the resulting differential electrochemical cell potential (V_{WE} – V_{RE}), and the AD5940's GPIO interrupt pin that momentarily pulses low whenever the FIFO threshold is reached, prompting the MCU to read the data. All waveforms illustrate the specific example where the FIFO threshold is set to one. The implementation of all electrochemical methods was mirrored to that of a PalmSens4 potentiostat.

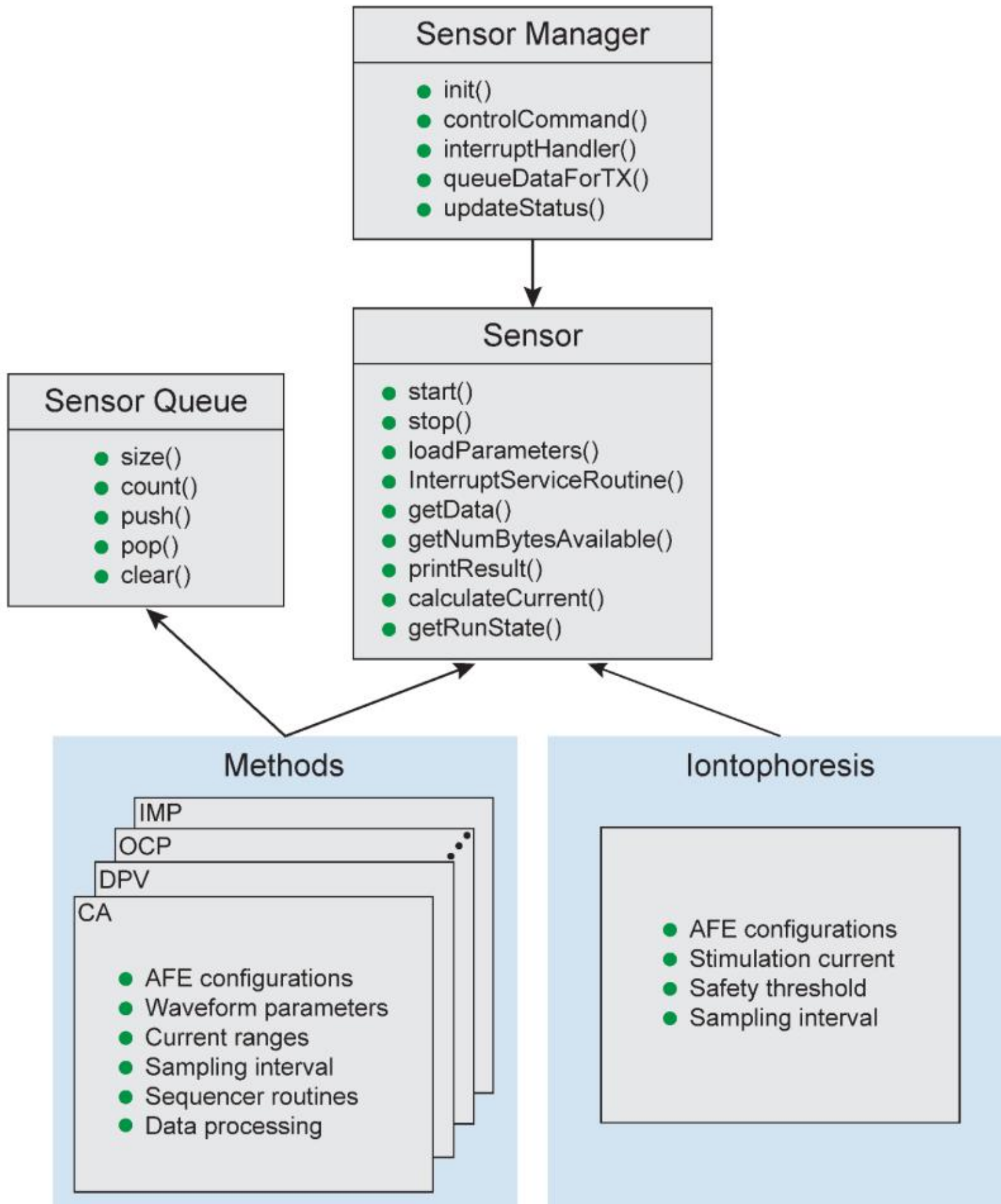

Supplementary Figure 13. Illustration of the device firmware's class-based organizational structure. Each gray box indicates a class that performs various functions or stores measurement-related parameters. Measurement-specific methods and iontophoresis are controlled by global sensor and sensor queue classes that handle tasks common to all methods and data queue operations (prior to BLE Tx), respectively. A sensor manager class sits on top of these to control the active technique.

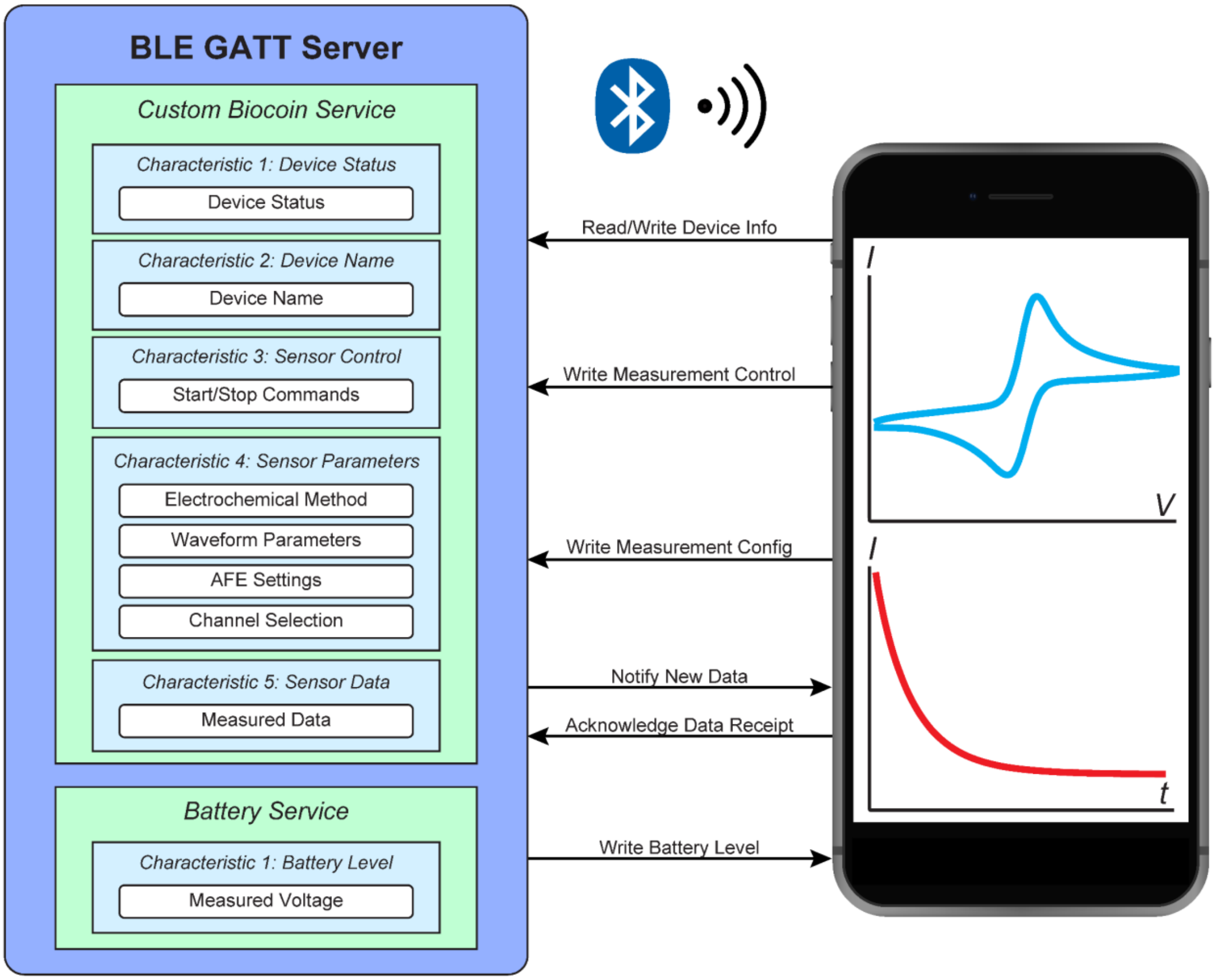

Supplementary Figure 14. BLE operation and custom GATT server configuration. Various services and characteristics are utilized to store device information, control sensor measurements, configure measurement parameters, transmit sensor data, and provide battery capacity updates. These characteristics allow a host device to seamlessly control all operations of the wearable device.

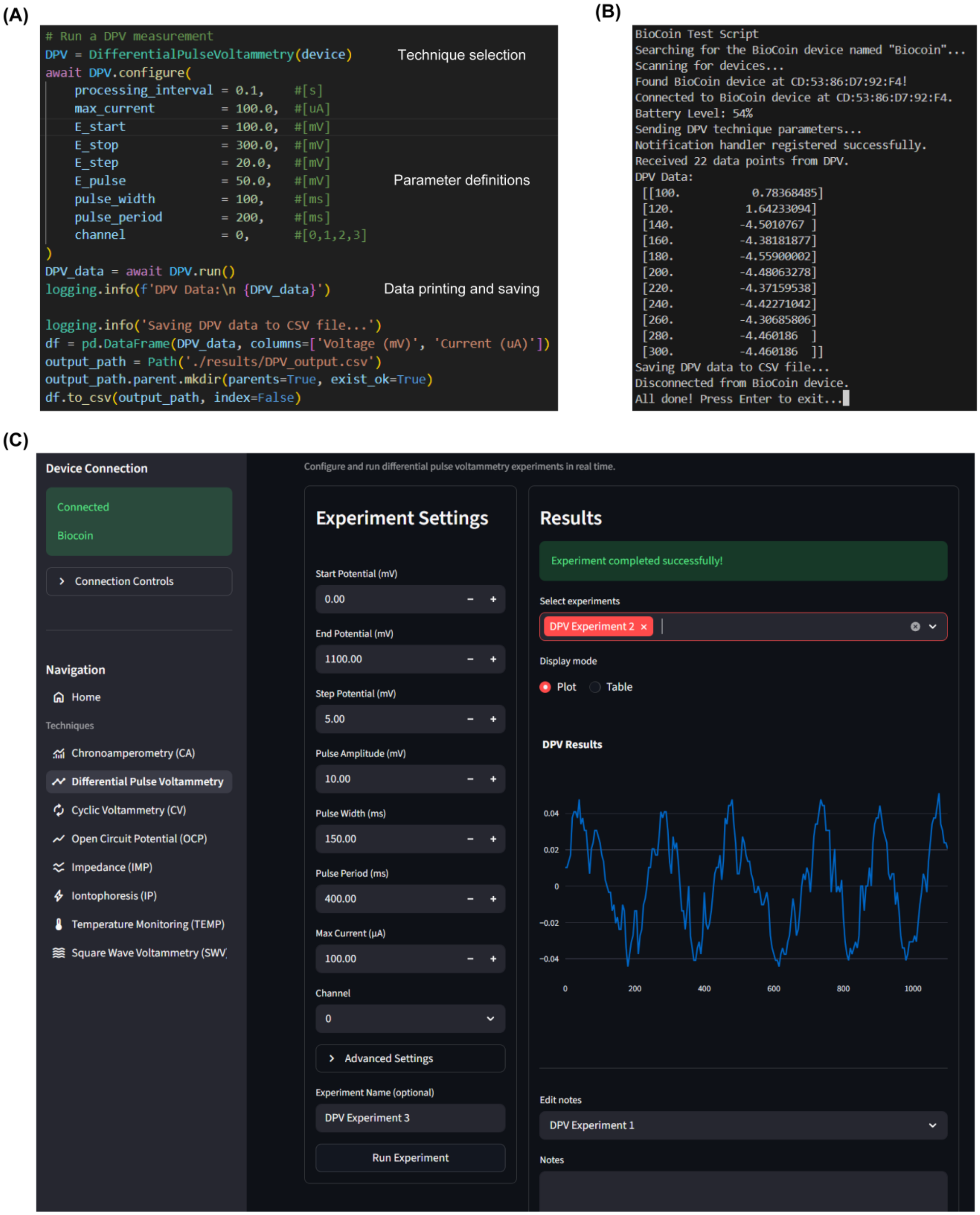

Supplementary Figure 15. Custom Python script and graphical user interface for wireless BLE control of Biocoin. (A) Image of the Python script interface running on a host computer, showcasing the measurement technique selection, parameter setting, and data logging configurations. The setup for DPV is shown as an example, similar setups exist for all supported methods. **(B)** The corresponding terminal printout during the executed DPV scan. **(C)** Image of the web-based graphical user interface enabling live plotting of sensor data.

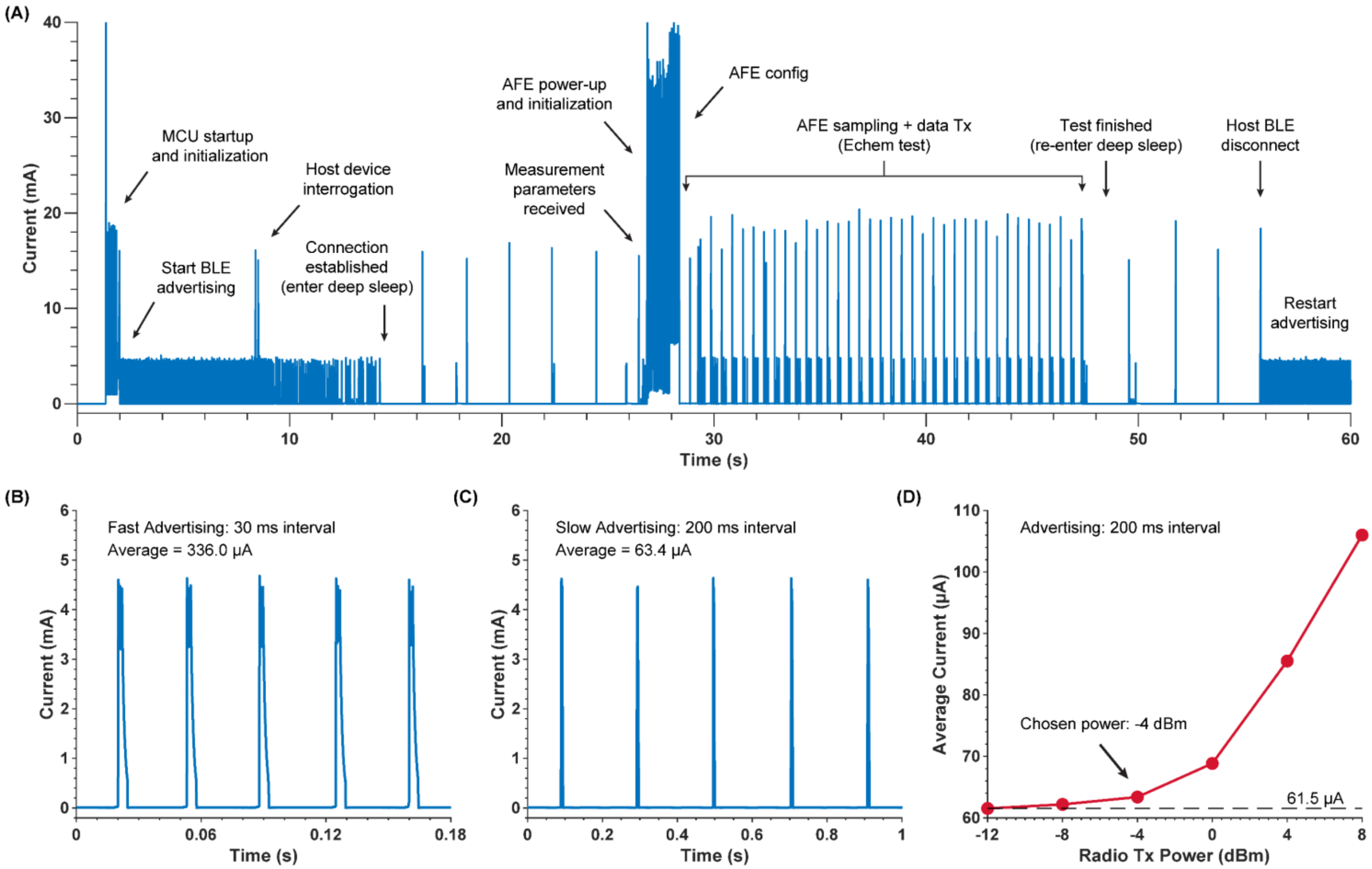

Supplementary Fig 16. Device startup and measurement sequence example routine and characterization during BLE advertising. (A) Annotated transient current profile of the wearable device as it cycles through different modes of operation while executing various functions, including startup, BLE advertising and connection, deep sleep, measurement configuration, data sampling, and wireless transmission. After power-up, all operations are initiated by the custom Python control script. **(B-D)** Characterization of the device during BLE advertising, including transient current profiles using a **(B)** 30 ms (fast) and **(C)** 200 ms (slow) advertising interval. **(D)** Average current of the device during advertising while operating with different BLE radio Tx powers. A 200 ms advertising interval was used for all power levels.

(A)

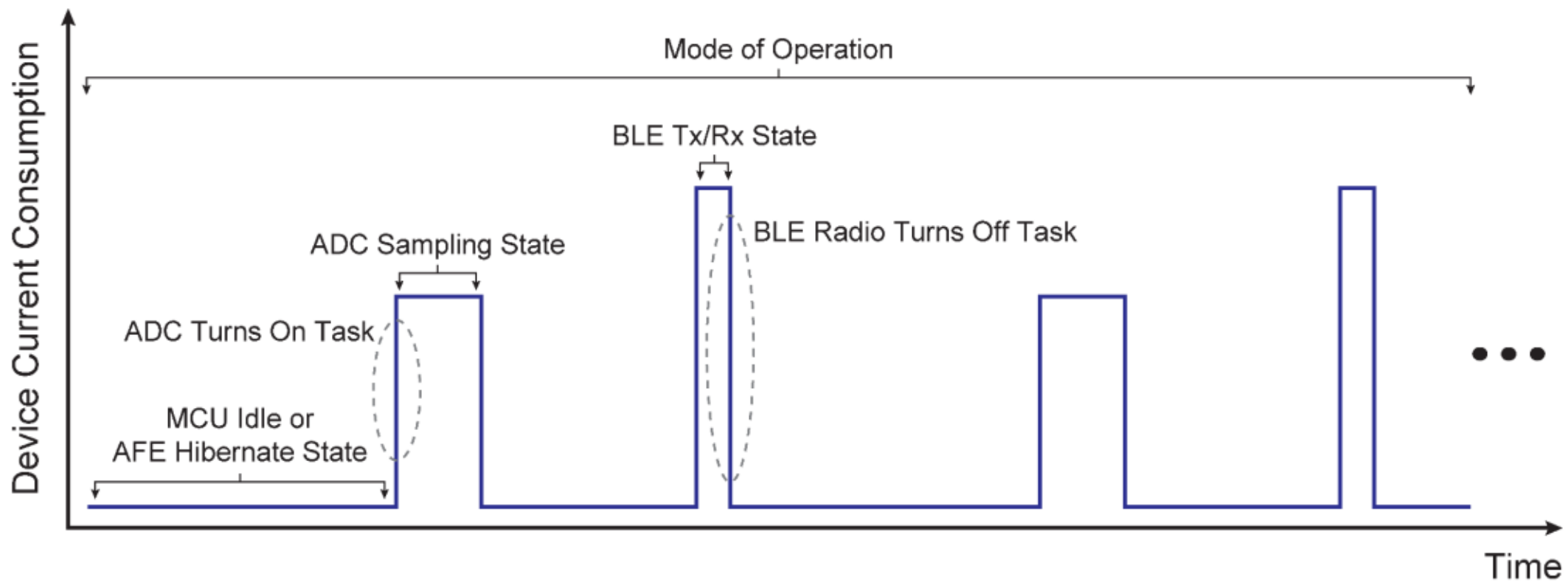

(B)

Deep Sleep Mode

Circuit Block	Block Status
Always-on blocks	
Buck-boost CH3	On
MCU	On [1]
Battery monitor	On
Analog Measurement blocks	
Buck-boost CH1	Off
LDO	Off
AD5940	Off
Voltage buffers	Off
4:1 MUX	Off
Voltage dividers	Off
Iontophoresis blocks	
Buck-boost CH2	Off
Boost converter	Off
Control opamp	Off
CSA	Off

Active Mode

Circuit Block	Block Status
Always-on blocks	
Buck-boost CH3	On
MCU	On [1]
Battery monitor	On
Analog Measurement blocks	
Buck-boost CH1	On
LDO	On
AD5940	Hibernating or Sampling [1]
Voltage buffers	On
4:1 MUX	On
Voltage dividers	On [1] / Off [1]
Iontophoresis blocks	
Buck-boost CH2	Off
Boost converter	Off
Control opamp	Off
CSA	Off

Iontophoresis Mode

Circuit Block	Block Status
Always-on blocks	
Buck-boost CH3	On
MCU	On [1]
Battery monitor	On
Analog Measurement blocks	
Buck-boost CH1	On
LDO	On
AD5940	Hibernating
Voltage buffers	On
4:1 MUX	On
Voltage dividers	Off
Iontophoresis blocks	
Buck-boost CH2	On
Boost converter	On
Control opamp	On
CSA	On

[1] Refer to **Supplementary Note 8** for descriptions of the different states that a particular block may enter during a given mode of operation.

Supplementary Figure 17. System modes of operation overview used for power consumption optimizations. (A) Representative Biocoin current consumption profile vs. time used to illustrate the distinction between device tasks, states, and modes of operation. **(B)** Summary of the operational status of all circuit blocks during the device's three modes of operation, highlighting the hardware's various levels of power-gating to minimize unnecessary power consumption.

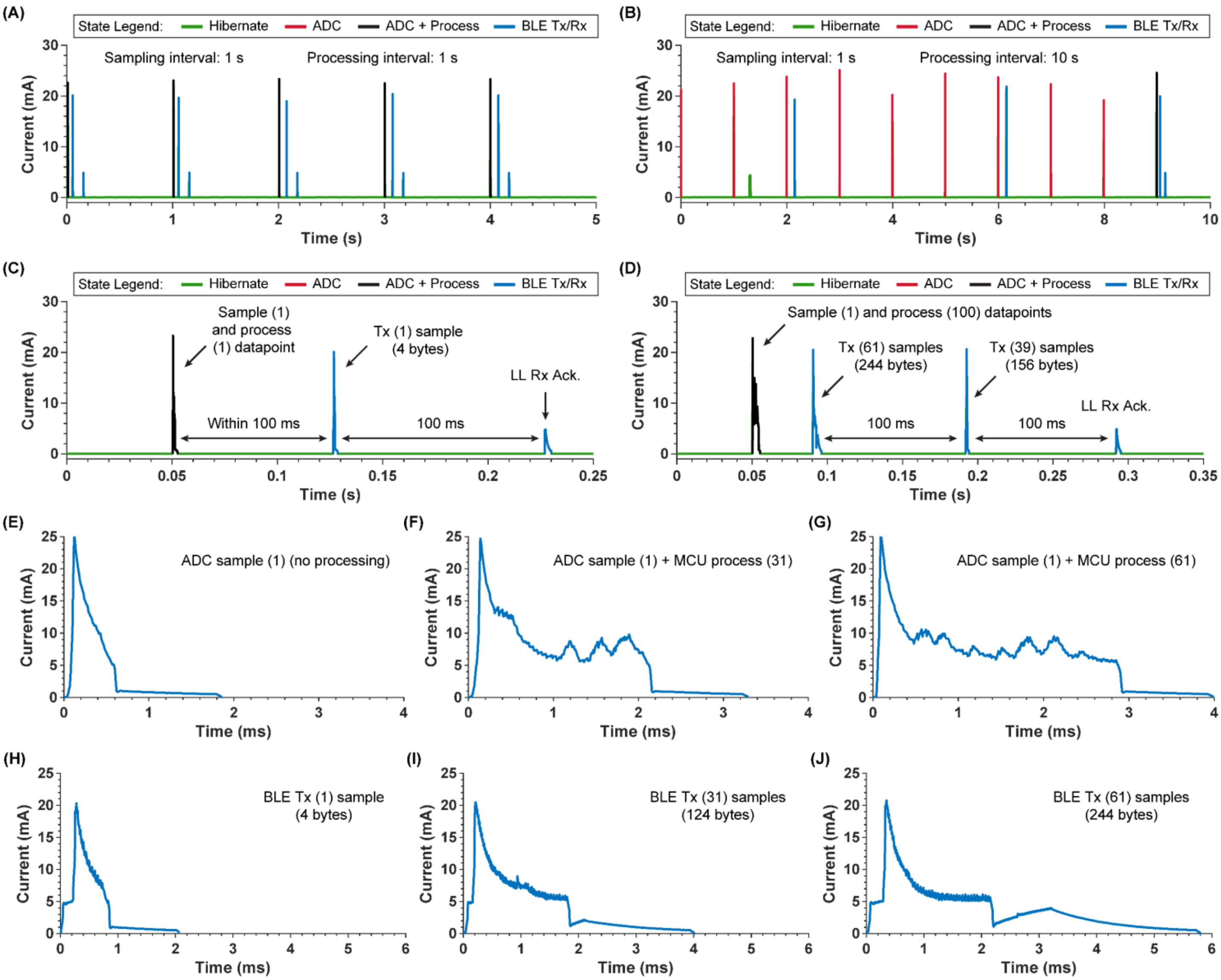

Supplementary Fig. 18. Illustration of the device's transient current consumption while performing different active mode operations with varying parameters. (A-D) Transient current profile examples during chronoamperometry with various sampling, processing, and BLE connection interval parameters. Example profiles are shown using chronoamperometry; however, the features apply behaviorally to all methods. All cases use the max MTU size of 247 bytes. Measurement results, including **(A)** a 1 s sampling interval, 1 s processing interval, 100 ms BLE connection interval, and a 4 s effective BLE connection interval, **(B)** a 1 s sampling interval, 10 s processing interval, 100 ms BLE connection interval, and a 4 s effective BLE connection interval, **(C)** a zoom in around the processing and BLE transmission of 1 data sample, followed by its associated receive acknowledgement, and **(D)** a zoom in around the processing and BLE transmission of 100 data samples, highlighting how the data Tx is now split into two sequential packets, separated by the BLE connection interval of 100 ms. **(E-G)** Zoomed-in instantaneous current consumption of AFE ADC sampling events with varying amounts of data processed by the MCU, including **(E)** current profiles when the AFE samples just 1 datapoint with: no processing (*i.e.,* MCU does not read the FIFO), **(F)** the processing of 31 samples, and **(G)** the processing of 61 samples. **(H-J)** Zoomed-in instantaneous current consumption of BLE connection events when transmitting varying amounts of data. Each data sample is sent as a float (4 bytes), with the MTU size set to 247 bytes (max). Current profiles when transmitting **(H)** just 1 data sample (4 bytes), **(I)** an approximately half-max payload of 31 data samples (124 bytes), and **(J)** a max payload of 61 data samples (244 bytes).

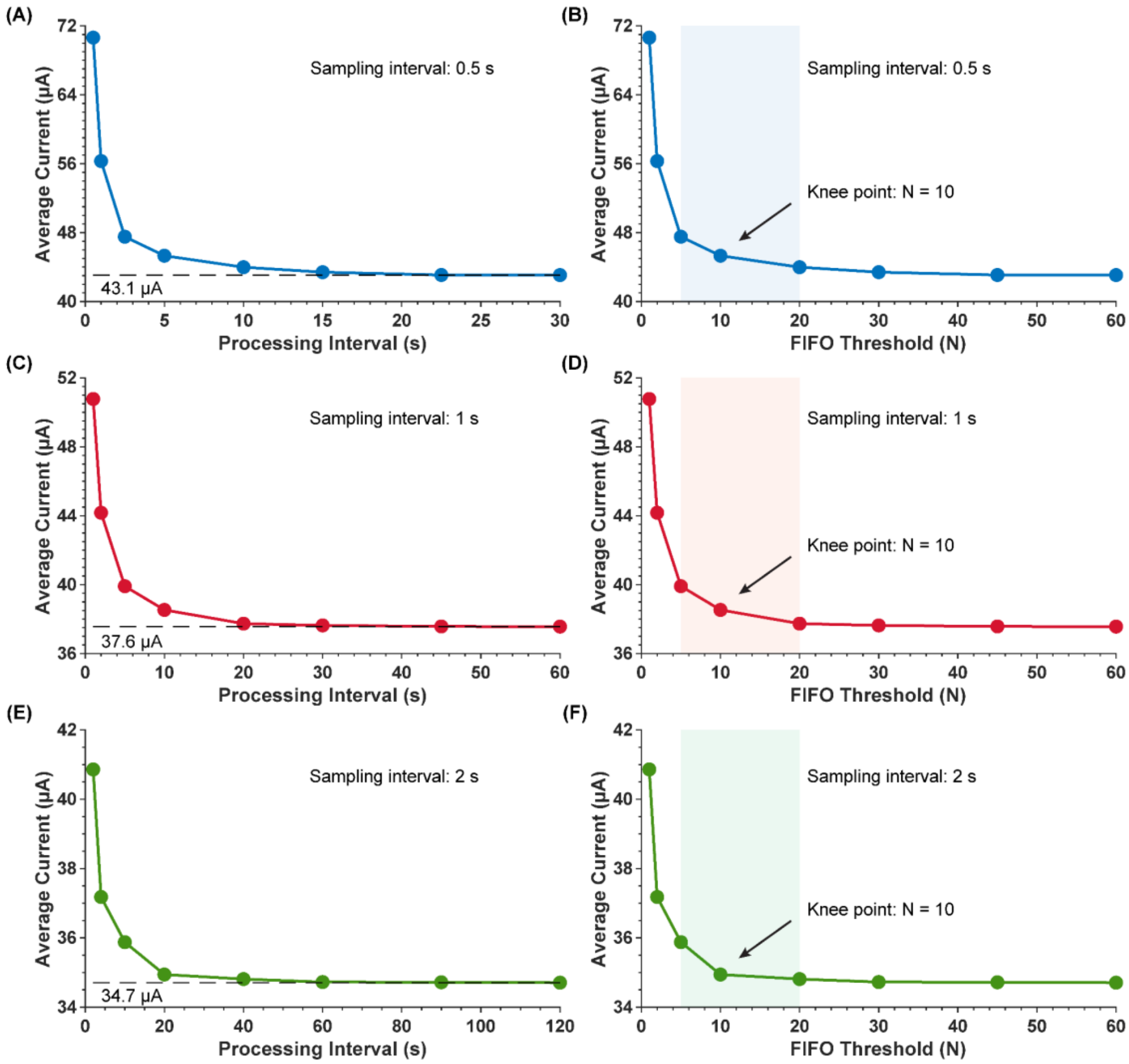

Supplementary Fig. 19. Optimization of the data processing interval parameter. (A) Average current vs. processing interval using a sampling interval of 0.5 s. **(B)** Average current data from (A) replotted vs. the corresponding FIFO threshold. **(C)** Average current vs. processing interval using a sampling interval of 1 s. **(D)** Average current data from (C) replotted vs. the corresponding FIFO threshold. **(E)** Average current vs. processing interval using a sampling interval of 2 s. **(F)** Average current data from (E) replotted vs. the corresponding FIFO threshold. All data in this study were collected using chronoamperometry as an example method; however, the general concept applies to any method. FIFO threshold = processing interval / sampling interval. A 100 ms BLE connection interval and a 4 s effective BLE connection interval were used for all cases. Shading highlights regions of optimal power–latency tradeoffs.

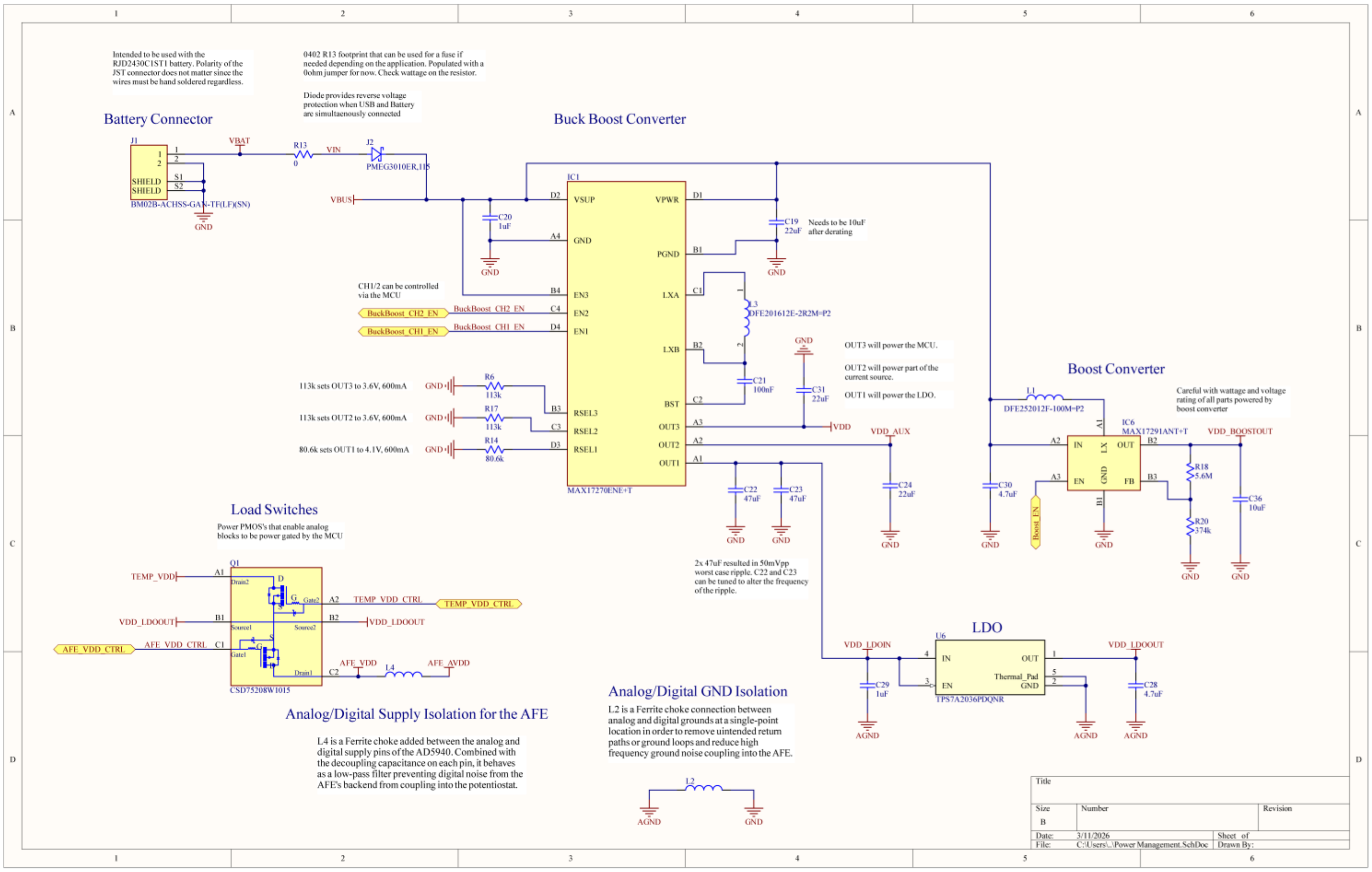

Supplementary Figure 20. Biocoin schematic: power management sheet.

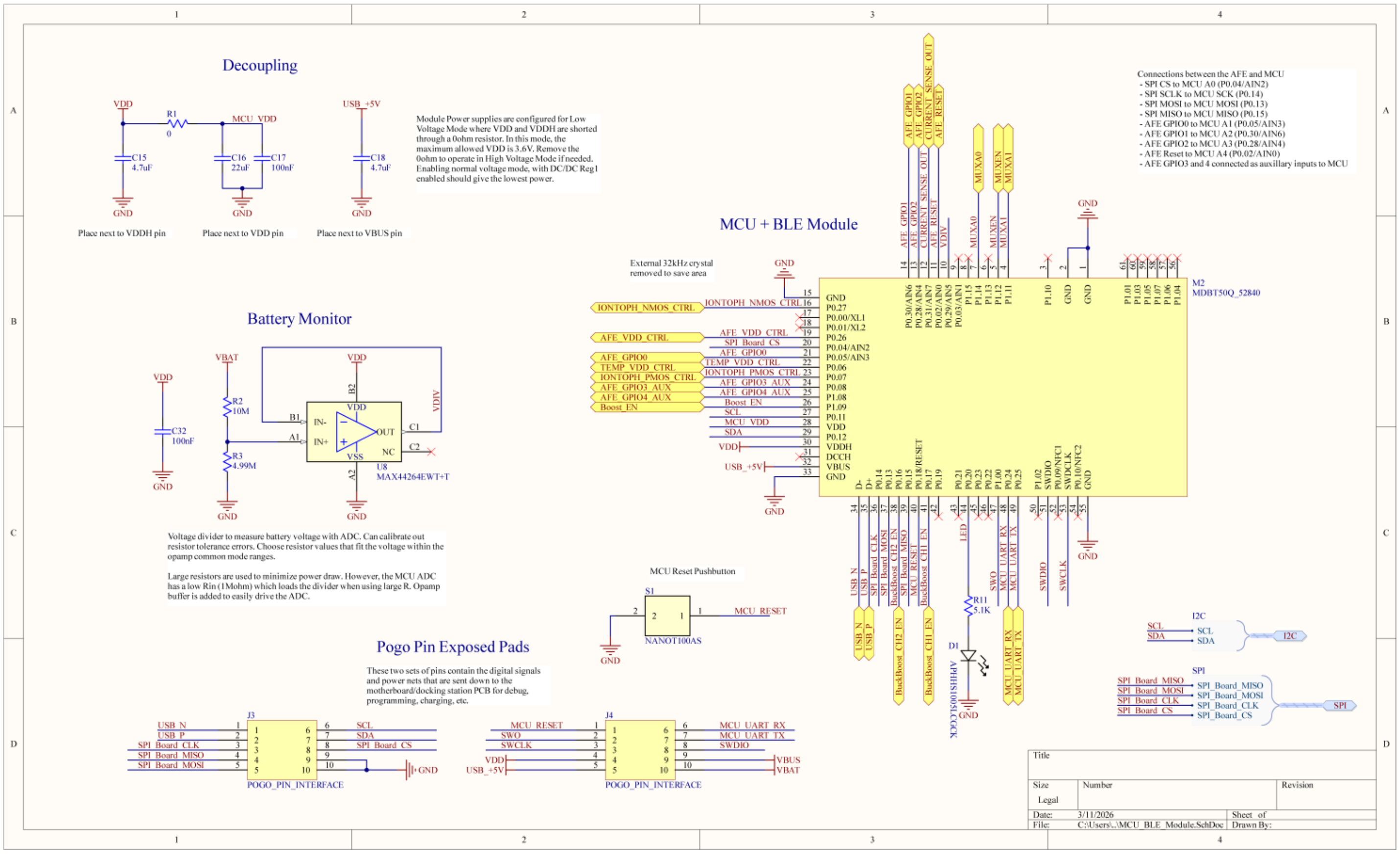

Supplementary Figure 21. Biocoin schematic: MCU and BLE module sheet.

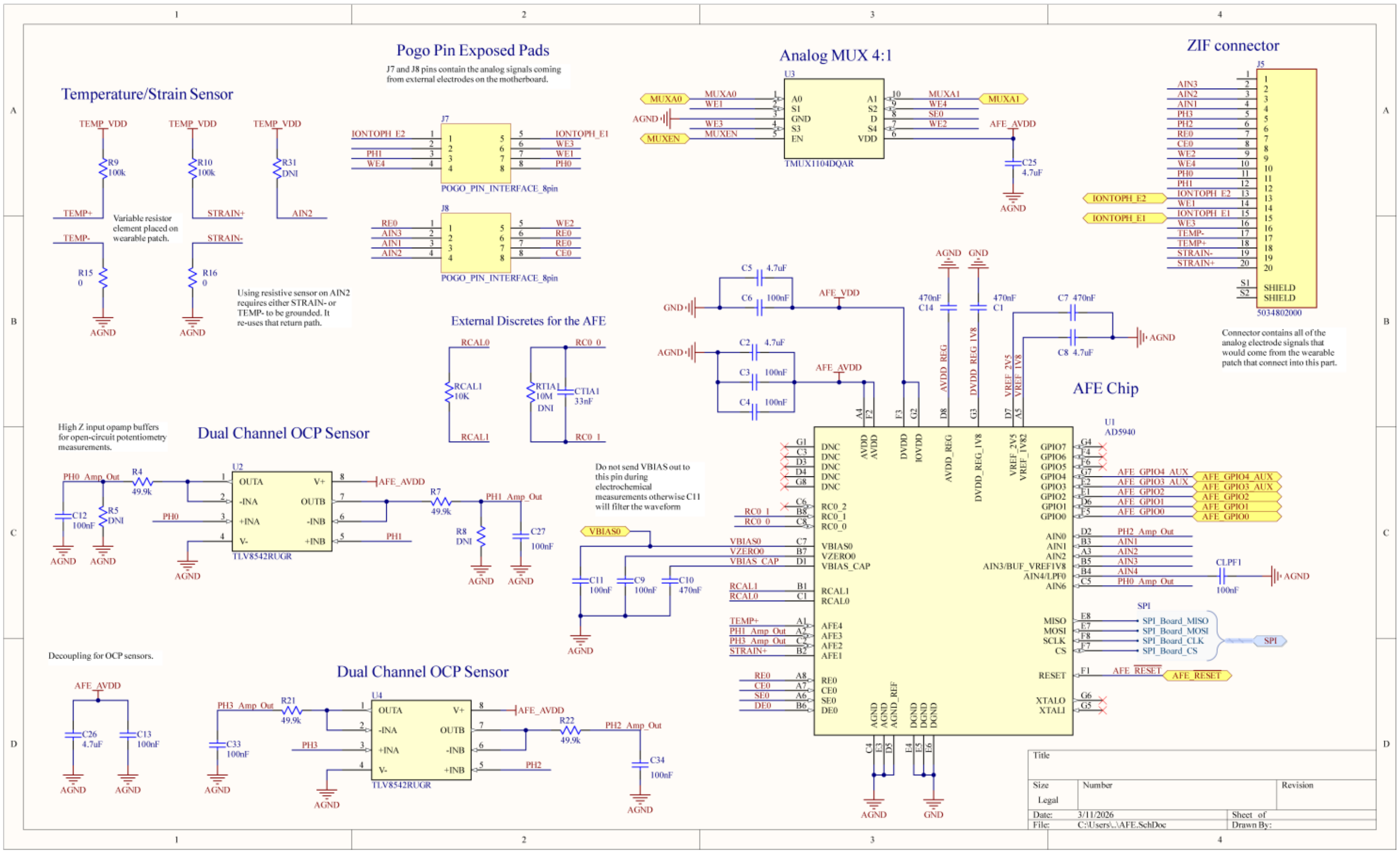

Supplementary Figure 22. Biocoin schematic: analog front-end sheet.

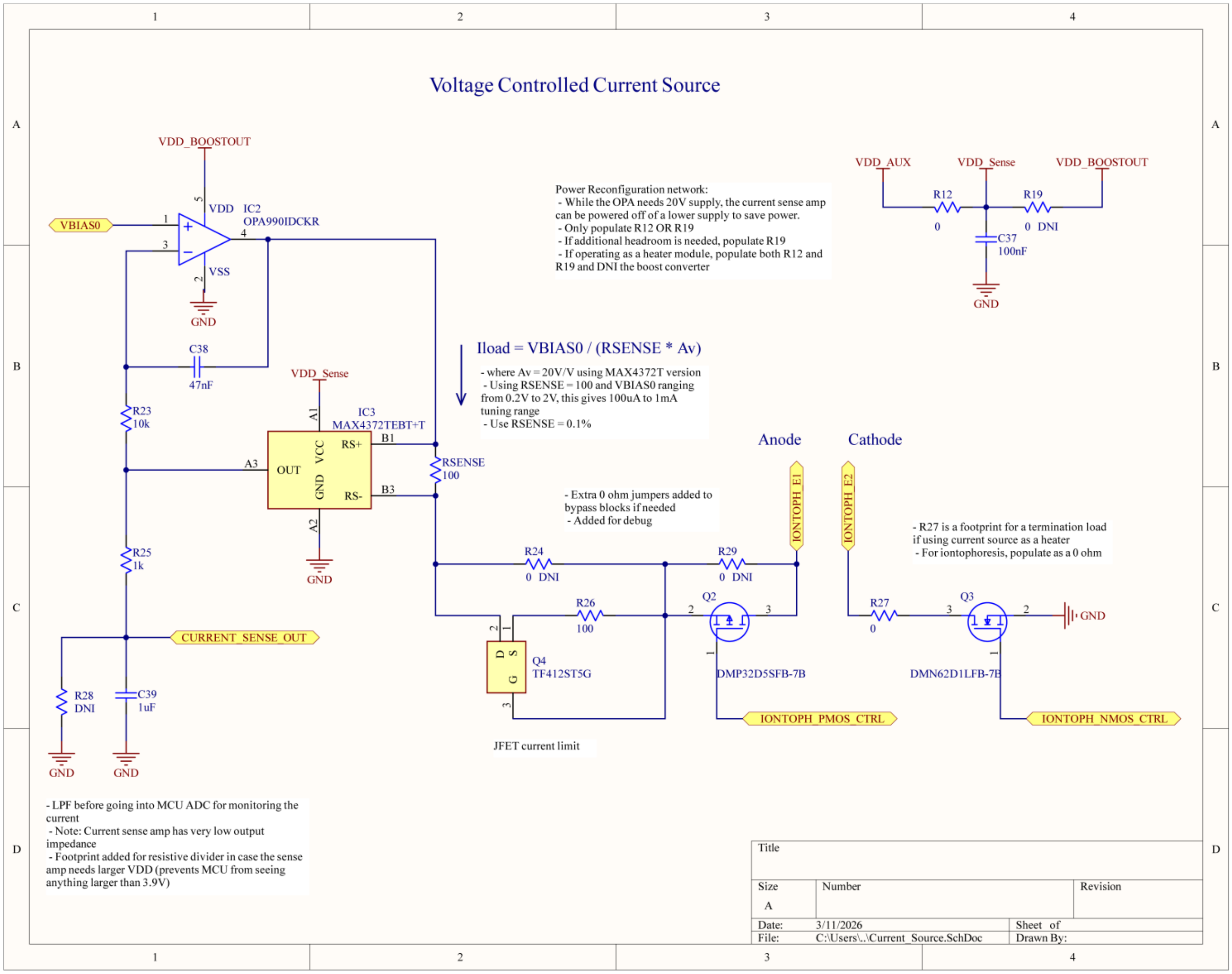

Supplementary Figure 23. Biocoin schematic: iontophoresis current source sheet.

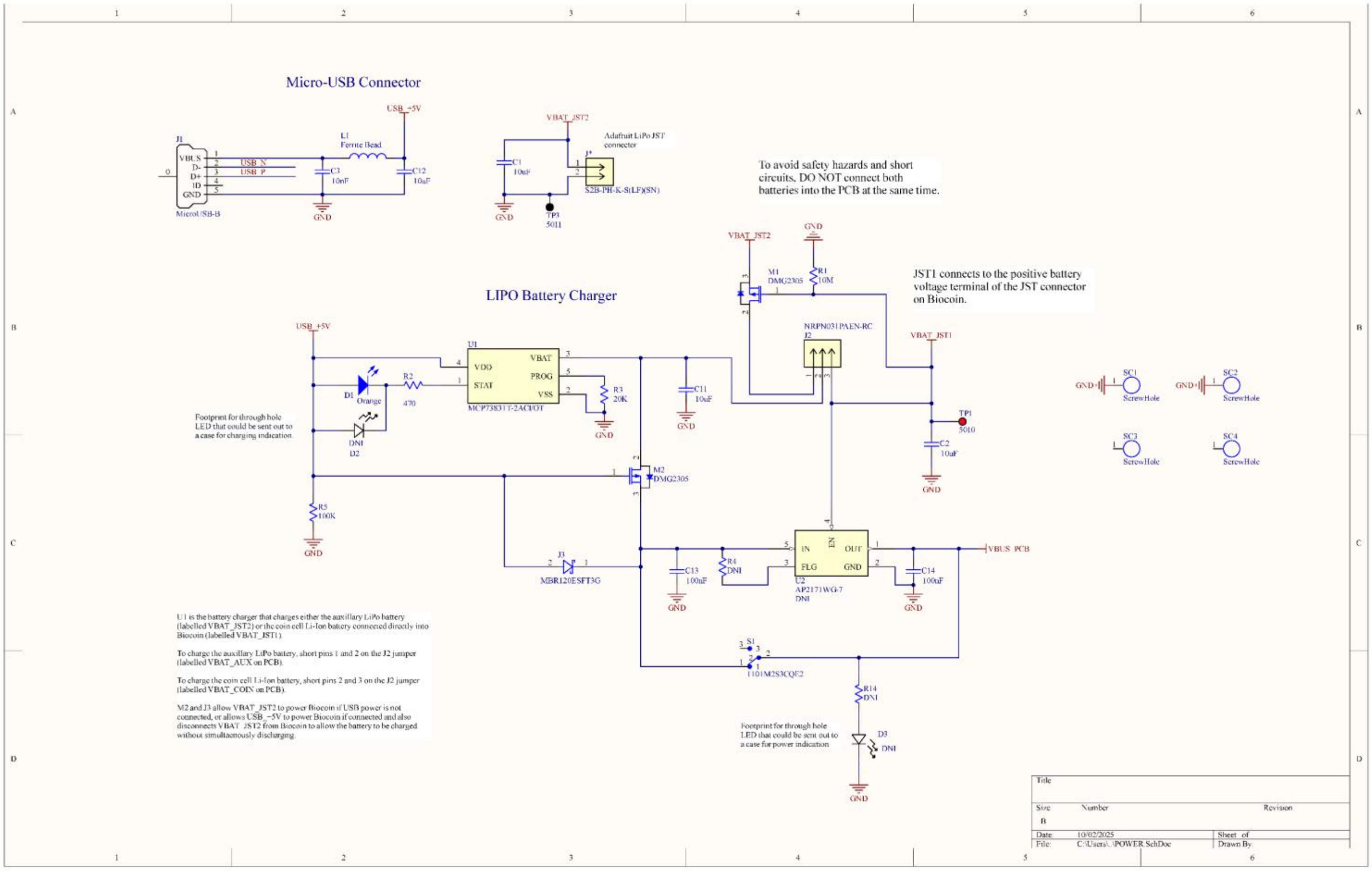

Supplementary Figure 24. Docking station schematic: power management sheet.

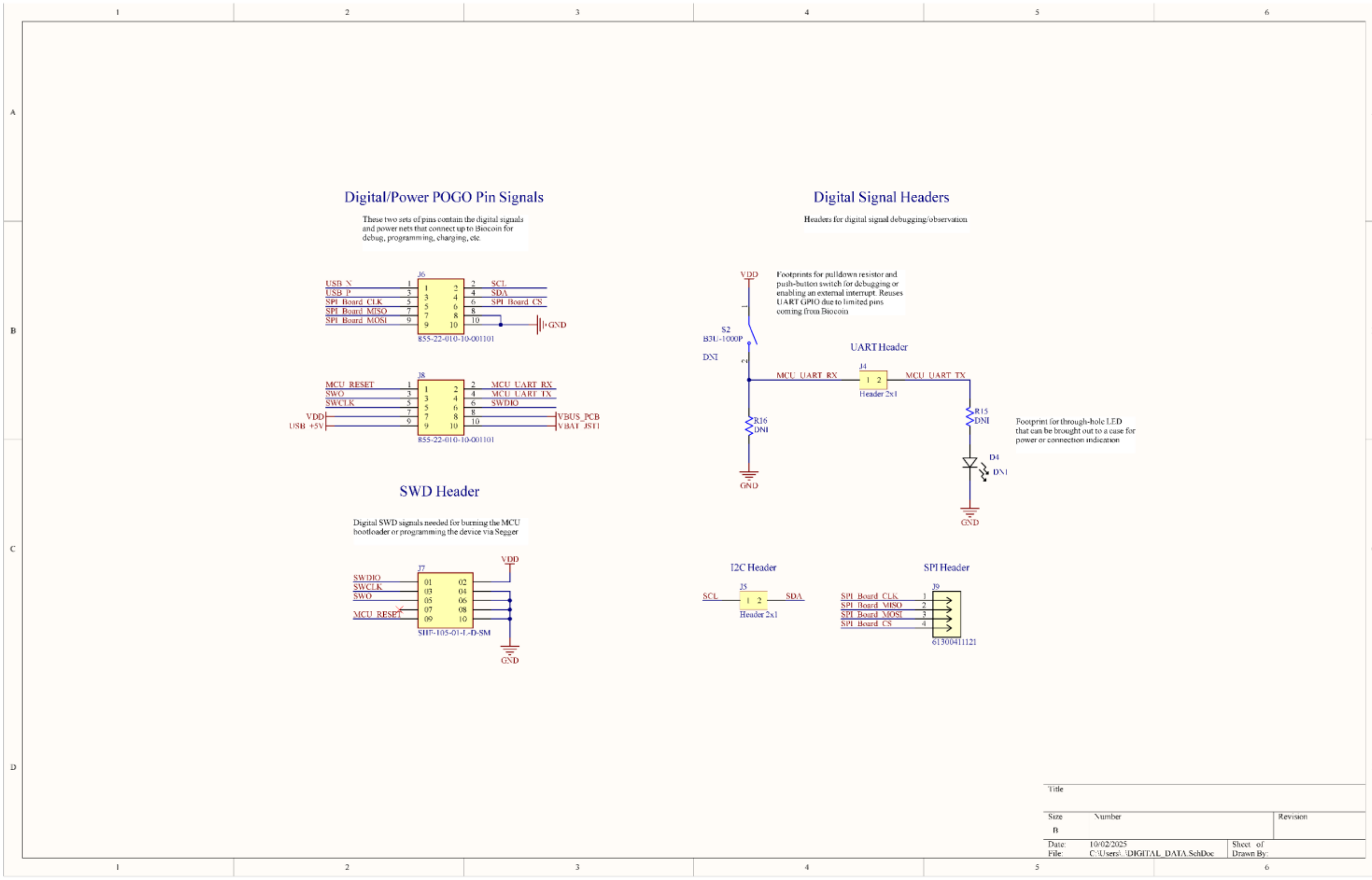

Supplementary Figure 25. Docking station schematic: digital signal interface sheet.

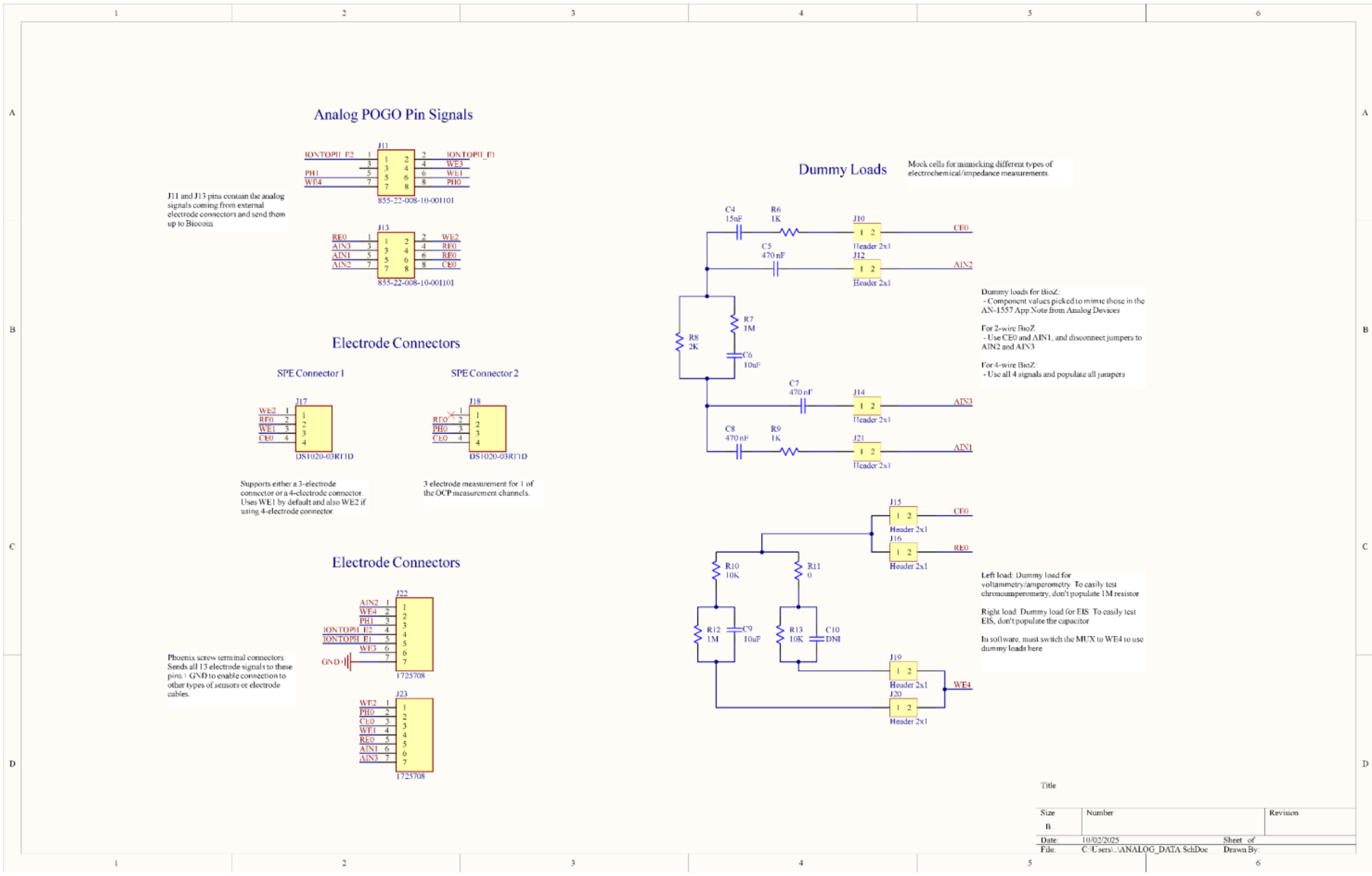

Supplementary Figure 26. Docking station schematic: analog signal interface sheet.

Supplementary Table 1. Comparison table of state-of-the-art wireless electronic systems for skin-interfaced biochemical sensing. Note: references [8,28,44,45] not included due to space considerations.

	Form Factor Device Area	Wireless Protocol	Power Source	Rechargeable? Circuit included?	User Interface	AFE Chipset(s)	Current in Lowest Power Mode [a]	Electrode Multiplexing	Electrochemical Methods	Other Hardware Sensors/Features	Open Source?	Year	Ref.
NFC-Powered	Patch (Flex) 1000 mm^2	NFC	NFC	N.A.	Mobile App	Discrete amplifiers	N.R.	1x WE 1x OCP	DPV, OCP	Temperature sensor Drug release module	No	2021	11
	Patch (Flex) 600 mm^2 [b]	NFC	NFC	N.A.	Mobile App	Discrete amplifiers	N.R.	1x WE 1x OCP	DPV, OCP	None	No	2024	10
	Patch (Flex) 3185 mm^2 [b]	NFC	NFC	N.A.	Mobile App	AD5940+AD8232 + LMP2231 + MAX31865	N.R.	1x WE	CA	Iontophoresis Temperature sensor ECG + GSR + RAP	No	2026	46
Alternatively-Powered	Patch (Flex) 1200 mm^2 [b]	BLE	Lactate BFC	Yes (Lactate) Yes (on wearable)	Mobile App	Discrete amplifiers	100 µA [d, e]	2x OCP	OCP	Temperature sensor 2x Resistive sensors	No	2020	1
	Patch (Flex) 380 mm^2 [b]	BLE	FTENG	Yes (on wearable) Yes (on wearable)	Mobile App	Discrete amplifiers	330 µA [e]	2x OCP	OCP	None	No	2020	2
	Patch (Flex) 550 mm^2 [b]	BLE	SABs	Yes (Sweat) Yes (on wearable)	Mobile App	Discrete amplifiers	N.R.	3x OCP	OCP	None	No	2022	6
	Patch (Flex) 380 mm^2 [b]	IR Comm. [c]	TEG	Yes (Body heat) Yes (on wearable)	Mobile/PC Terminal	AD8233 + ADXL362	1.8 µA [f, g]	2x OCP	OCP	ECG sensor Motion sensor	No	2023	3
	Patch (Flex) 540 mm^2	BLE	Perovskite solar cell	Yes (Light) Yes (on wearable)	Mobile App	AD5941 + discrete amplifiers	27.3 µA [f]	1x WE 2x OCP	CA, DPV, OCP	Iontophoresis 1x Resistive sensor Impedimetry	No	2023	5
	Patch (Flex) 1267 mm^2	BLE	MEG	Yes (Moisture) Yes (on wearable)	Mobile/PC Terminal	Discrete amplifiers + ADXL362	767 µA [d, f, h]	1x WE 1x OCP	DPV, OCP	Motion sensor	No	2024	4
	Patch (Flex) 662.5 mm^2	BLE	Wi-Fi + 30 mAh LiPo	Yes (Wi-Fi) Yes (on wearable)	Mobile App	LMP91000 + MAX30102	8,100 µA [f]	1x WE	CA	Temperature sensor PPG sensor	No	2024	7
	Ring (Flex) 324 mm^2	BLE	Lactate BFC + AgCl-Zn battery	Yes (Lactate) Yes (on wearable)	Mobile App	nRF52832 (MCU ADC)	5 µA [d]	4x OCP	OCP	None	No	2024	47
Commercial Battery-Powered	Wristband (Flex) 2400 mm^2 [b]	BL classic	105 mAh LiPo	Yes No	Mobile App	Discrete amplifiers	N.R.	2x WE 2x OCP	CA, OCP	1x Resistive sensor	No	2016	48
	Wristband (Flex) 2400 mm^2 [b]	BL classic	LiPo (capacity N.R.)	Yes No	Mobile App	Discrete amplifiers	N.R.	1x WE 2x OCP	CA, OCP	Iontophoresis	No	2017	26
	Wristband (Rigid) 1256 mm^2 [b]	BLE	150 mAh LiPo	Yes (USB), Yes (on wearable)	N.R.	LMP91000 + LMP91200	60 µA	12x WE 1x OCP	CA, CV, OCP	1x Resistive sensor	No	2020	49
	Patch (Hybrid-flex) 2000 mm^2 [b]	BLE	150 mAh LiPo	Yes No	Mobile App / LCD	LMP91000	7,000 µA	6x WE	CA	Iontophoresis (6x channels)	No	2020	27
	Patch (Flex) 3000 mm^2 [b]	BLE	190 mAh LiPo	Yes No	MATLAB App	Discrete amplifiers	N.R.	6x WE or OCP	SWV, OCP	1x Resistive sensor	No	2021	50
	Patch (Flex) 1323 mm^2 [b]	BLE	110 mAh Li-Ion	Yes No	Mobile App / LCD	LMP91000	N.R.	3x WE	Swept V_{GS} FET sensing	1x Resistive sensor	No	2022	51
	Patch (Flex) 855 mm^2	BLE	12 mAh LiPo	Yes No	Python script / Mobile App	AD5941	16.1 µA [d, f]	1x WE	CA	None	No	2022	35
	Patch (Rigid) 530 mm^2	BLE	110 mAh Li-Ion	Yes (Wireless), Yes (on wearable)	Mobile App	AD5940	53.5 µA	3x or 4x WE [i]	CA	None	No	2022	41
	Patch (Flex) 1500 mm^2 [b]	BL classic	LiPo (capacity N.R.)	Yes (USB), Yes (on wearable)	Mobile App	Discrete amplifiers	28,000 µA	2x WE 1x OCP	DPV, OCP	Iontophoresis 1x Resistive sensor	No	2022	32
	Patch (Flex) 930 mm^2	BLE	8 mAh Lithium	Yes No	N.R.	AD5941 + discrete amplifiers	N.R.	3x WE 2x OCP	CA, OCP	1x Resistive sensor Drug delivery module	No	2023	52
	Patch (Flex) 808 mm^2	BLE	8 mAh Lithium	Yes No	Mobile App	AD5941 + discrete amplifiers	N.R.	1x WE 1x OCP	SWV, OCP	Iontophoresis 1x Resistive sensor Impedimetry	No	2023	34
	Ring (Flex) 598 mm^2	BLE	8 mAh Lithium	Yes No	Mobile App	AD5941	26.3 µA [f]	1x WE 1x OCP	SWV, OCP	Iontophoresis Impedimetry	No	2024	53
	Patch (Flex) 1079 mm^2 [b]	BLE	Lithium (capacity N.R.)	N.R.	N.R.	Discrete amplifiers	N.R.	3x WE 3x OCP	CA, OCP	Iontophoresis 1x Resistive sensor GSR + CDC sensors	No	2024	33
	Patch (Rigid) 225 mm^2	BLE	CR2032	No No	N.R.	ADuCM355	333.3 µA [f]	1x WE	Swept V_{GS} FET sensing	None	No	2024	54
	Patch (Rigid) 589 mm^2 [b]	Wi-Fi	150 mAh LiPo	Yes No	PC App	AD5941 + ADS1299	63,000 µA	2x WE 2x OCP	CA, OCP	EMG sensor	No	2025	55
	Patch (Flex) 1384 mm^2 [b]	BLE	Lithium (capacity N.R.)	Yes No	Mobile App	AD5941 + discrete amplifiers	N.R.	15x WE 1x OCP	CA, OCP, SWV, EIS	Iontophoresis 1x Resistive sensor Impedimetry	No	2025	56
	Patch (Flex) 933 mm^2 [b]	BLE	LiPo (capacity N.R.)	Yes No	Mobile Terminal	Discrete amplifiers	N.R.	4x WE	CA	Insulin release module	No	2025	57
	Patch (Rigid) 2171 mm^2 [b]	BLE	300 mAh Li-Ion	Yes (USB) Yes (on wearable)	Mobile App	AD5941 + ADS1115	N.R.	3x WE 3x OCP	CA, OCP	None	No	2026	58
	Patch (Flex) 1891 mm^2 [b]	BLE	100 mAh LiPo	Yes No	Mobile App	Discrete amplifiers	216.2 µA [f]	1x WE 1x OCP	CA, OCP	Iontophoresis 1x Resistive sensor rSO_2 [l] sensor	No	2026	59
	**Patch (Rigid) 530 mm^2**	**BLE**	**110 mAh Li-Ion**	**Yes (USB), Yes (on docking station)**	**PC App / Python script**	**AD5940 + discrete amplifiers**	**12.7 µA [j] 10.9 µA [k]**	**4x WE 4x OCP**	**CA, CV, DPV, SWV, LSV, NPV, OCP, EIS**	**Iontophoresis 3x Resistive sensors Impedimetry**	**Yes**	**2026**	**This Work**

N.R. = Not reported; N.A. = Not applicable; BL = Bluetooth; BLE = Bluetooth low energy; AFE = Analog front-end; LiPo = Lithium polymer; Li-Ion = Lithium ion; PC = Personal computer; USB = Universal serial bus; NFC = Near field communication; FET = Field effect transistor; LCD = Liquid crystal display; WE = Working electrode; CA = Chronoamperometry; CV = Cyclic voltammetry; OCP = Open-circuit potentiometry; DPV = Differential pulse voltammetry; SWV = Square wave voltammetry; LSV = Linear sweep voltammetry; NPV = Normal pulse voltammetry; EIS = Electrochemical impedance spectroscopy; FTENG = Freestanding triboelectric nanogenerator; SABs = Sweat-activated batteries; MEG = Moisture-enabled electricity generator; CDC = Capacitance-to-digital converter; IR Comm. = Infrared communication; GSR = Galvanic skin response; PPG = Photoplethysmography; TEG = Thermoelectric generator; ADC = Analog-to-digital converter; EMG = Electromyography; ECG = Electrocardiogram; BFC = Biofuel cell; MCU = Microcontroller; RAP = Radial artery pulse;

[a] Unless stated otherwise, for BLE devices, this includes the average current required to maintain a connection and/or transmit data periodically. [b] Estimated from figure. [c] Infrared communication via On-Off-Key modulated LED blinks.
[d] Does not include average current required to maintain a BLE connection or transmit data. [e] Continuous BLE connection not supported. Data transmitted intermittently via advertising. [f] Calculated from reported power consumption and nominal supply voltage.
[g] Does not include average current of periodic IR communication, or the additional losses incurred by the energy harvesting circuitry. [h] Includes the current of MCU and potentiostat/ADC blocks. [i] 3x WE for a 3-electrode setup, 4x WE for a 2-electrode setup.
[j] MCU and AFE powered at 3.6 V. [k] MCU and AFE powered at 3.0 V. [l] Region tissue oxygenation.

Supplementary Table 2. Comparison table of open-source potentiostat-based electrochemical systems.

Form Factor Device Area	Wireless Protocol	Power Source	Rechargeable? Circuit included?	User Interface	Electrode Multiplexing	Electrochemical Methods	Iontophoresis Support?	Other Hardware Sensors	Sleep Mode Power-Gating Addressed?	Current in Lowest Power Mode	Ref.
Stamp-sized 540 mm^2	None (wired)	Battery	Yes No	PC App	1x WE	CA, CV, SWV, LSV, ASV	No	None	No	20,000 µA	12
Palm-sized 2253 $mm^{2\,a}$	BLE	Battery	Yes Yes (USB)	Mobile App	1x WE 1x OCP	CA, CV, DPV, SWV, OCP	No	None	No	4,000 µA	13
Arduino-sized 1161 mm^2	Wi-Fi	Battery	Yes Yes (USB)	PC App	1x WE	CA, CV, DPV, SWV, LSV, NPV	No	None	No	N.R.	16
Palm-sized 2258 $mm^{2\,a}$	BL classic	Battery	No No	Mobile App	1x WE	CV, SWV	No	None	No	N.R.	17
Coin-sized 438.5 mm^2	None (wired)	USB	N.A.	N.R.	1x WE	CA, CV, SWV, NPV	No	None	No	11,000 µA	18
Credit card-sized 3618 mm^2	BLE	Battery	Yes Yes (USB)	Mobile App	1x WE	CV, LSV	No	None	No	5,000 µA	19
Arduino-sized 1480 $mm^{2\,a}$	Wi-Fi	Battery	N.R.	Mobile / PC App	4x WE	CA, CV	No	None	No	N.R.	20
Credit card-sized 4000 $mm^{2\,a}$	None (wired)	Battery	No No	LCD / PC App	1x WE	CA, CV, SWV, ASV	No	None	No	N.R.	21
Hand-sized 7728 mm^2	None (wired)	USB	N.A.	PC App	1x WE	CA, CV, OCP	No	None	No	N.R.	24
**Coin-sized 530 mm^2**	**BLE**	**Battery**	**Yes Yes (USB)**	**PC App / Python script**	**4x WE 4x OCP**	**CA, CV, DPV, SWV, LSV, NPV, OCP, EIS**	**Yes**	**3x Resistive Sensors Impedimetry**	**Yes**	**12.7 µA [b] 10.9 µA [c]**	**This Work**

N.R. = Not reported; N.A = Not applicable; BLE = Bluetooth low energy; BL = Bluetooth; PC = Personal computer; WE = Working electrode;
USB = Universal serial bus; LCD = Liquid crystal display; OCP = Open circuit potentiometry; LSV = Linear sweep voltammetry; NPV = Normal pulse voltammetry; ASV = Anodic stripping voltammetry;
CA = Chronoamperometry; CV = Cyclic voltammetry; DPV = Differential pulse voltammetry; SWV = Square wave voltammetry; EIS = Electrochemical impedance spectroscopy;
[a] Estimated from figure. [b] MCU and AFE powered at 3.6 V. [c] MCU and AFE powered at 3.0 V.

Supplementary Table 3. Summary of all electrochemical, resistive, and impedance measurement methods performed by Biocoin, and the supported values and ranges of their associated parameters. Minimum sampling intervals are defined based on the limits for acquiring and transmitting data reliably, without packet loss, using the buffered and real-time transmission strategies outlined in **Supplementary Note 9**.

Measurement Method	Parameter	Supported Values [a]
Chronoamperometry (CA)	Potential Pulse	-2.2V to 2.2V
	Pulse Length	≥ 3ms
	Sampling Interval	≥ 3ms
Differential Pulse Voltammetry (DPV)	Potential Scan Range (Estart – Estop) [b]	ΔV ≤ 2.2V [c]
	Estep	≥ 0.537mV
	Epulse	≥ Estep + 0.537mV
	Pulse Width	≥ 3ms
	Pulse Period	≥ Pulse Width + 3ms
Square Wave Voltammetry (SWV)	Potential Scan Range (Estart – Estop) [b]	ΔV ≤ 2.2V [c]
	Estep	≥ 0.537mV
	Amplitude	≥ Estep
	Pulse Width	≥ 3ms
Cyclic Voltammetry (CV)	Potential Scan Range (abs{Evertex2 – Evertex1}) [b]	ΔV ≤ 2.2V [c]
	Estep	≥ 0.537mV
	Pulse Width	≥ 3ms
Linear Sweep Voltammetry (LSV)	Potential Scan Range (Estart – Estop) [b]	ΔV ≤ 2.2V [c]
	Estep	≥ 0.537mV
	Pulse Width	≥ 3ms
Normal Pulse Voltammetry (NPV)	Potential Scan Range (Estart – Estop) [b]	ΔV ≤ 2.2V [c]
	Estep	≥ 0.537mV
	Pulse Width	≥ 3ms
	Pulse Period	≥ Pulse Width + 3ms
Open Circuit Potentiometry (OCP)	Run Time	≥ 3ms
	Sampling Interval	≥ 3ms
Temperature / Resistive Sensor (TEMP)	Run Time	≥ 3ms
	Sampling Interval	≥ 3ms
Impedance (IMP)	Run Time	≥ 3ms
	Sampling Interval	≥ 3ms
	Eac	≥ 0.293mV
	Frequency [d]	≤ 250kHz
Electrochemical Impedance Spectroscopy (EIS)	Sampling Interval	≥ 3ms
	Eac	≥ 0.293mV
	Edc	≥ 0.537mV
	Frequency_{start} [d]	≤ 250kHz
	Frequency_{stop} [d]	≥ Frequency_{start}
	Number of Frequency Points	≤ 101

[a] For voltage-based parameters, the resolution is 0.537mV (the AD5940's low-power 12-bit DAC resolution). The only exceptions are the Eac parameters for IMP and EIS, which utilize the high-speed 12-bit DAC that has an amplitude resolution of 0.293mV and maximum output of 600mV. For time-based parameters, the resolution is one cycle of the 32kHz low frequency internal oscillator (~31μs).

[b] Refers to the supported dynamic potential window of the electrochemical cell (V_{WE}-V_{RE}) that can be swept over during a scan. Allowable voltage range is dictated by the AD5940's DAC output ranges. Note that the maximum range does not account for 1) any over/under-potential required by the counter electrode or 2) additional voltage headroom required by the AD5940's TIA.

[c] For example, -1.1V to 1.1V, 0V to 2.2V, -2.2V to 0V, etc.

[d] The frequency resolution is 0.015Hz.

Supplementary Table 4. Comparison of low-power BLE-compatible microcontroller chipsets (modules) considered during the Biocoin design phase. Key parameters relevant to wearable sensing platforms are summarized, including module size, memory resources, radio current consumption, peripheral features, and development ecosystem support. All the compared chipset options are also available in packaged modules (including the SoC, antenna, and other RF components), which simplifies the PCB layout phase. The nRF52840 was selected for this design because it offers a strong balance among power consumption, Flash/RAM capacity, and module dimensions, and is the only option with Arduino-compatible toolchain support (nRF52 board support package (BSP)). This ecosystem support simplifies firmware development and reduces the embedded systems knowledge required to build on this open-source platform.

**Comparison of MCU + BLE Chipsets**						
**Chipset**	**nRF52840**	**CC2651R3**	**CYBLE-222014-01**	**EFR32BG24**	**BlueNRG-2**	**STM32WB55**
**Manufacturer**	Nordic Semi.	Texas Instruments	Infineon	Silicon Labs	ST Micro.	ST Micro.
**Representative module / package**	MDBT50Q-1MV2 (Raytac) (chip antenna)	CC2651R3SIPA (Texas Instruments) (integrated antenna)	CYBLE-222014-01 (Infineon) (chip antenna)	RF-BM-BG24B1 (RF-star) (PCB antenna)	BlueNRG-M2SA (ST Micro.) (chip antenna)	STM32WB5MMG (ST Micro.) (chip antenna)
**Module / package dimensions**	10.5 × 15.5 mm	7 × 7 mm	10 × 10 mm	23.35 × 17 mm	11.5 × 13.5 mm	7.3 × 11 mm
**Flash memory**	1 MB	352 kB	256 kB	1.024 MB	256 kB	1 MB
**RAM**	256 kB	32 kB	32 kB	128 kB	24 kB	256 kB
**Sleep current**	~2.4 μA	~2.4 μA	~1.5 μA	~4.1 μA	~0.9 μA	~2.5 μA
**MCU active current**	3.1 mA @ 64 MHz	2.9 mA @ 48 MHz	13.4 mA @ 48 MHz	1.9 mA @ 39 MHz	1.9 mA @ 32 MHz	5.0 mA @ 64 MHz
**BLE Tx current (0 dBm)**	~6.4 mA	~7.1 mA	~15.6 mA	~5.0 mA	~9.27 mA	~5.2 mA
**BLE Rx current**	~6.26 mA	~6.4 mA	~16.4 mA	~4.7 mA	~7.55 mA	~4.5 mA
**Bluetooth version**	BLE 5.4	BLE 5.2	BLE 5.1	BLE 5.3	BLE 5.2	BLE 5.3
**MCU performance**	Cortex-M4 @ 64 MHz	Cortex-M4 @ 48 MHz	Cortex-M3 @ 48 MHz	Cortex-M33 @ 39 MHz	Cortex-M0 @ 32 MHz	Cortex-M4 @ 64 MHz
**Relevant analog / digital peripherals**	ADC, SPI, I^2C, UART, timers, GPIOs	ADC, SPI, I^2C, UART, timers, GPIOs	ADC, SPI, I^2C, UART, timers, GPIOs	ADC, SPI, I^2C, UART, timers, GPIOs	ADC, SPI, I^2C, UART, timers, GPIOs	ADC, SPI, I^2C, UART, timers, GPIOs
**Development ecosystem**	Nordic SDK + Zephyr + Arduino BSP	TI SimpleLink SDK	Cypress PSoC Creator	Simplicity Studio + Gecko SDK	ST BlueNRG SDK + STM32Cube	STM32CubeWB
**Arduino-compatible toolchain availability?**	**Yes (Adafruit nRF52 BSP)**	No	No	No	No	No

Supplementary Table 5. Comparison of dedicated electrochemical front-end chipsets considered during the Biocoin design phase. The table summarizes critical wearable system parameters including package size, quiescent current, current measurement capabilities, and availability of on-board digital backend resources. The AD5940 was selected for this design as it provides the most favorable combination of low quiescent power, compact package size, high-performance current measurement, and integrated data converters and sequencing.

Comparison of Dedicated Electrochemical Front-end Chipsets				
**Component**	**AD5940 [A] /AD5941 [B] / ADuCM355 [C]**	**LMP91000**	**SIC4341**	**EmStat Pico Module**
**Manufacturer**	Analog Devices	Texas Instruments	Silicon Craft Technology	PalmSens / Analog Devices
**Size**	3.6 mm × 4.2 mm [A]<br>7 mm × 7 mm [B]<br>6 mm × 5 mm [C]	4.0 mm × 4.0 mm	1.2 mm × 1.2 mm	30.5 mm × 18 mm
**Quiescent current (potentiostat front-end only)**	~10 μA	~10 μA	N.R.	N.R.<br>(< 100 μA estimated)
**Current measurement range**	~200 nA – 3 mA<br>(26 ranges)	5 μA – 750 μA<br>(7 ranges)	2.5 μA – 20 μA<br>(2 ranges)	100 nA – 5 mA<br>(12 ranges)
**Current measurement resolution**	5.5 pA	Depends on external ADC	5 nA	5.5 pA
**Integrated ADC?**	Yes	No	Yes	Yes
**Integrated DAC?**	Yes	No	Yes	Yes
**Auxiliary analog input multiplexing support built-in?**	Yes	No	No	Yes
**Sleep mode functionality built-in?**	Yes	Yes	N.R.	Yes
**Digital backend sequencer and memory built-in?**	Yes (sequencer)<br>Yes (memory)	No (sequencer)<br>No (memory)	No (sequencer)<br>Yes (memory)	Yes (sequencer)<br>Yes (memory)

[A, B, C] All three chips are based on the same analog front-end circuitry. The ADuCM355 also integrates an MCU (no BLE).
N.R. = Not reported in datasheet

Supplementary Table 6. Summary of the wearable device's average current consumption and estimated battery lifetime during different modes and states of operation, with varying operational parameters and analog supply voltages ($V_{DD,A}$). In all cases, the voltage of $V_{DD,D}$ equals $V_{DD,A}$, the electronic system was powered at 3.7 V (V_{BAT}), and a 110 mAh battery capacity is assumed. TEMP, temperature; IMP, impedance.

Mode of Operation		Method Parameters		Average Current (µA)		Battery Lifetime (mo)	
				3.6 V $V_{DD,A}$	3.0 V $V_{DD,A}$	3.6 V $V_{DD,A}$	3.0 V $V_{DD,A}$
Deep Sleep		BLE $Conn_{eff}$ Interval	1 s	17.0	15.0	9.0	10.2
			4 s	12.7	10.9	12.0	14.0
			10 s	12.1	10.3	12.6	14.8
Active	CA	Sampling Interval	1 s	38.7	26.7	3.9	5.7
			5 s	33.3	22.2	4.6	6.9
			10 s	32.7	21.6	4.7	7.1
	DPV	Pulse Period	0.1 s	170.8	160.4	0.9	1.0
			0.5 s	58.0	49.4	2.6	3.1
			1 s	45.6	36.9	3.4	4.1
	OCP	Sampling Interval	1 s	37.3	24.8	4.1	6.2
			5 s	32.0	20.2	4.8	7.6
			10 s	31.5	19.7	4.9	7.8
	TEMP	Sampling Interval	1 s	36.3	20.6	4.2	7.4
			5 s	31.2	16.2	4.9	9.4
			10 s	30.4	15.6	5.0	9.8
	IMP	Sampling Interval	1 s	548.3	413.4	0.3	0.4
			5 s	133.3	95.1	1.1	1.6
			10 s	81.3	55.1	1.9	2.8
**Mode of Operation**		**Load Condition**		**Average Current (mA)** – 3.6 V $V_{DD,A}$ and 20 V Compliance			
Iontophoresis		Load Current	0 (no load)	0.98			
			0.05 mA	1.21			
			0.5 mA	4.23			
**Sleep State**				**Average Current (µA)**			
				3.6 V $V_{DD,A}$		3.0 V $V_{DD,A}$	
MCU Idle				5.7		4.6	
AFE Hibernate				25.1		14.8	

Supplementary Table 7. Bill of materials for all components on the Biocoin PCB ($V_{DD,A}$ = 3.6 V). This BoM configures the system's analog and digital supply voltages ($V_{DD,A}$ and $V_{DD,D}$) to 3.6 V.

Manufacturer Part Number	Description	Designator	Quantity on PCB	Value	Do Not Install	Unit Price ($USD) (@ Quantity 10x)	Unit Price ($USD) (@ Quantity 100x)	Supplier
C0603X5R1E473K030BB	Multilayer Chip Capacitor 0201	C38	1	47nF		0.039	0.0227	Digikey
C0603X5R1E104K030BB	Multilayer Chip Capacitor 0201	C37	1	100nF		0.043	0.0253	Digikey
C0201C104K9PAC7867	Multilayer Chip Capacitor 0201	C3, C4, C6, C12, C13, C17, C21, C27, C32, C33, C34, CLPF1	12	100nF		0.045	0.0263	Digikey
C0603X5R1A474M030BC	Multilayer Chip Capacitor 0201	C1, C7, C10, C14	4	470nF		0.102	0.063	Digikey
0201X105K6R3CT	Multilayer Chip Capacitor 0201	C20, C29, C39	3	1uF		0.105	0.105	Digikey
GRM035R60J475ME15D	Multilayer Chip Capacitor 0201	C2, C5,C8, C15, C18, C25, C26, C28, C30	9	4.7uF		0.112	0.0701	Digikey
C0402C333J4RACAUTO	Multilayer Chip Capacitor 0402	CTIA1	1	33nF		0.165	0.1174	Digikey
GRT155R71H104KE01D	Multilayer Chip Capacitor 0402	C9, C11	2	100nF		0.029	0.0164	Digikey
04026D226MAT2A	Multilayer Chip Capacitor 0402	C16, C19, C24, C31	4	22uF		0.315	0.2118	Digikey
GRM188R61E106KA73J	Multilayer Chip Capacitor 0603	C36	1	10uF		0.142	0.09	Digikey
GRM188R60J476ME15D	Multilayer Chip Capacitor 0603	C22, C23	2	47uF		0.088	0.088	Digikey
N/A	Surface Mount Resistor 0201	R5, R8, R28, R31	4	N/A	DNI	0	0	
RMCF0201ZT0R00	Surface Mount Resistor 0201	R1, R12, R15, R16, R27	5	0Ω		0.006	0.0048	Digikey
RMCF0201ZT0R00	Surface Mount Resistor 0201	R19, R24, R29	3	0Ω	DNI	0	0	
RC0201FR-071KL	Surface Mount Resistor 0201	R25	1	1Ω		0.008	0.005	Digikey
RMCF0201FT5K10	Surface Mount Resistor 0201	R11	1	5.1kΩ		0.005	0.0048	Digikey
RMCF0201FT10K0	Surface Mount Resistor 0201	R23	1	10kΩ		0.01	0.007	Digikey
RE0201BRE0710KL	Surface Mount Resistor 0201	RCAL1	1	10kΩ		0.234	0.1632	Digikey
RMCF0201FT49K9	Surface Mount Resistor 0201	R4, R7, R21, R22	4	49.9kΩ		0.007	0.0046	Digikey
RC0201FR-0780K6L	Surface Mount Resistor 0201	R14	1	80.6kΩ		0.009	0.0066	Digikey
RE0201BRE07100KL	Surface Mount Resistor 0201	R9, R10	2	100kΩ		0.199	0.1408	Digikey
ERJ-1GNF1133C	Surface Mount Resistor 0201	R6, R17	2	113kΩ		0.039	0.0202	Digikey
ERJ-1GNF3743C	Surface Mount Resistor 0201	R20	1	374kΩ		0.039	0.0202	Digikey
CRCW02015M60FNED	Surface Mount Resistor 0201	R18	1	5.6MΩ		0.041	0.0209	Digikey
ERJ-2GE0R00X	Surface Mount Resistor 0402	R13	1	0Ω		0.014	0.0087	Digikey
ERJ-2RKF1000X	Surface Mount Resistor 0402	R26	1	100Ω		0.028	0.0138	Digikey
RP73PF1E100RBTD	Surface Mount Resistor 0402	RSENSE	1	100Ω		0.135	0.1124	Digikey
CRCW04024M99FKED	Surface Mount Resistor 0402	R3	1	4.99MΩ		0.034	0.0176	Digikey
RC0402FR-0710ML	Surface Mount Resistor 0402	R2	1	10MΩ		0.004	0.0032	Digikey
RC0402FR-0710ML	Surface Mount Resistor 0402	RTIA1	1	10MΩ	DNI	0	0	
MMZ0603S601HT000	Surface Mount Ferrite 0201	L2, L4	2	-		0.085	0.0593	Digikey
DFE252012F-100M=P2	Surface Mount Inductor	L1	1	10uH		0.2	0.1645	Digikey
DFE201612E-2R2M=P2	Surface Mount Inductor	L3	1	2.2uH		0.2	0.1645	Digikey
APHHS1005LCGCK	Green LED 0402	D1	1	-		0.194	0.1366	Digikey
MAX17270ENE+T	Buck-Boost Converter	IC1	1	-		5.066	4.2687	Digikey
OPA990IDCKR	Opamp	IC2	1	-		0.809	0.6459	Digikey
MAX4372TEBT+T	Current Sense Amplifier	IC3	1	-		2.499	2.0641	Digikey
MAX17291ANT+T	Boost Converter	IC6	1	-		2.069	1.6995	Digikey
BM02B-ACHSS-GAN-TF(LF)(SN)	Connector Header Surface Mount	J1	1	-		0.419	0.3557	Digikey
PMEG3010ER,115	Diode Schottky	J2	1	-		0.228	0.1435	Digikey
N/A, Exposed pads	Surface Mount Header Pin Pad	J3, J4	2	-	DNI	0	0	
5034802000	20 Position FFC, FPC Connector	J5	1	-		1.404	1.1931	Digikey
N/A, Exposed pads	Surface Mount Header Pin Pad	J7, J8	2	-	DNI	0	0	
MDBT50Q-1MV2	MDBT50Q_52840	M2	1	-		6.15	6.15	Digikey
CSD75208W1015	-20-V, P channel NexFET	Q1	1	-		0.473	0.3053	Digikey
DMP32D5SFB-7B	MOSFET (P-Channel)	Q2	1	-		0.277	0.175	Digikey
DMN62D1LFB-7B	MOSFET (N-Channel)	Q3	1	-		0.215	0.1347	Digikey
TF412ST5G	N-ch JFET Transistor	Q4	1	-		0.315	0.1998	Digikey
NANOT100AS	Smallest Push Button Switch	S1	1	-		0.358	0.2993	Digikey
AD5940BCBZ-RL	Electrochemical Front End	U1	1	-		10.66	9.4095	Digikey
TLV8542RUGR	Low Input Bias Current Opamp	U2, U4	2	-		0.942	0.7556	Digikey
TMUX1104DQAR	4:1 Analog MUX	U3	1	-		2.189	1.8016	Digikey
TPS7A2036PDQNR	Small Low Iq LDO	U6	1	-		0.25	0.1923	Digikey
MAX44264EWT+T	Small Low Iq Opamp	U8	1	-		1.485	1.2077	Digikey

**Total Unit Price per PCB ($USD) @ Quantity 10x :**	**42.81**
**Total Unit Price per PCB ($USD) @ Quantity 100x :**	**36.02**

Supplementary Table 8. Bill of materials for all components on the Biocoin PCB ($V_{DD,A}$ = 3.0 V). This BoM configures the system's analog and digital supply voltages ($V_{DD,A}$ and $V_{DD,D}$) to 3.0 V. Only the components highlighted in blue have changed relative to those in **Supplementary Table 7**.

Manufacturer Part Number	Description	Designator	Quantity on PCB	Value	Do Not Install	Unit Price ($USD) (@ Quantity 10x)	Unit Price ($USD) (@ Quantity 100x)	Supplier
C0603X5R1E473K030BB	Multilayer Chip Capacitor 0201	C38	1	47nF		0.039	0.0227	Digikey
C0603X5R1E104K030BB	Multilayer Chip Capacitor 0201	C37	1	100nF		0.043	0.0253	Digikey
C0201C104K9PAC7867	Multilayer Chip Capacitor 0201	C3, C4, C6, C12, C13, C17, C21, C27, C32, C33, C34, CLPF1	12	100nF		0.045	0.0263	Digikey
C0603X5R1A474M030BC	Multilayer Chip Capacitor 0201	C1, C7, C10, C14	4	470nF		0.102	0.063	Digikey
0201X105K6R3CT	Multilayer Chip Capacitor 0201	C20, C29, C39	3	1uF		0.105	0.105	Digikey
GRM035R60J475ME15D	Multilayer Chip Capacitor 0201	C2, C5,C8, C15, C18, C25, C26, C28, C30	9	4.7uF		0.112	0.0701	Digikey
C0402C333J4RACAUTO	Multilayer Chip Capacitor 0402	CTIA1	1	33nF		0.165	0.1174	Digikey
GRT155R71H104KE01D	Multilayer Chip Capacitor 0402	C9, C11	2	100nF		0.029	0.0164	Digikey
04026D226MAT2A	Multilayer Chip Capacitor 0402	C16, C19, C24, C31	4	22uF		0.315	0.2118	Digikey
GRM188R61E106KA73J	Multilayer Chip Capacitor 0603	C36	1	10uF		0.142	0.09	Digikey
GRM188R60J476ME15D	Multilayer Chip Capacitor 0603	C22, C23	2	47uF		0.088	0.088	Digikey
N/A	Surface Mount Resistor 0201	R5, R8, R28, R31	4	N/A	DNI	0	0	
RMCF0201ZT0R00	Surface Mount Resistor 0201	R1, R12, R15, R16, R27	5	0Ω		0.006	0.0048	Digikey
RMCF0201ZT0R00	Surface Mount Resistor 0201	R19, R24, R29	3	0Ω	DNI	0	0	
RC0201FR-071KL	Surface Mount Resistor 0201	R25	1	1Ω		0.008	0.005	Digikey
RMCF0201FT5K10	Surface Mount Resistor 0201	R11	1	5.1kΩ		0.005	0.0048	Digikey
RMCF0201FT10K0	Surface Mount Resistor 0201	R23	1	10kΩ		0.01	0.007	Digikey
RE0201BRE0710KL	Surface Mount Resistor 0201	RCAL1	1	10kΩ		0.234	0.1632	Digikey
RMCF0201FT49K9	Surface Mount Resistor 0201	R4, R7, R21, R22	4	49.9kΩ		0.007	0.0046	Digikey
ERJ-1GNF1333C	Surface Mount Resistor 0201	R14	1	133kΩ		0.039	0.0202	Digikey
RE0201BRE07100KL	Surface Mount Resistor 0201	R9, R10	2	100kΩ		0.199	0.1408	Digikey
ERJ-1GNF1623C	Surface Mount Resistor 0201	R6, R17	2	162kΩ		0.039	0.0202	Digikey
ERJ-1GNF3743C	Surface Mount Resistor 0201	R20	1	374kΩ		0.039	0.0202	Digikey
CRCW02015M60FNED	Surface Mount Resistor 0201	R18	1	5.6MΩ		0.041	0.0209	Digikey
ERJ-2GE0R00X	Surface Mount Resistor 0402	R13	1	0Ω		0.014	0.0087	Digikey
ERJ-2RKF1000X	Surface Mount Resistor 0402	R26	1	100Ω		0.028	0.0138	Digikey
RP73PF1E100RBTD	Surface Mount Resistor 0402	RSENSE	1	100Ω		0.135	0.1124	Digikey
CRCW04024M99FKED	Surface Mount Resistor 0402	R3	1	4.99MΩ		0.034	0.0176	Digikey
RC0402FR-0710ML	Surface Mount Resistor 0402	R2	1	10MΩ		0.004	0.0032	Digikey
RC0402FR-0710ML	Surface Mount Resistor 0402	RTIA1	1	10MΩ	DNI	0	0	
MMZ0603S601HT000	Surface Mount Ferrite 0201	L2, L4	2	-		0.085	0.0593	Digikey
DFE252012F-100M=P2	Surface Mount Inductor	L1	1	10uH		0.2	0.1645	Digikey
DFE201612E-2R2M=P2	Surface Mount Inductor	L3	1	2.2uH		0.2	0.1645	Digikey
APHHS1005LCGCK	Green LED 0402	D1	1	-		0.194	0.1366	Digikey
MAX17270ENE+T	Buck-Boost Converter	IC1	1	-		5.066	4.2687	Digikey
OPA990IDCKR	Opamp	IC2	1	-		0.809	0.6459	Digikey
MAX4372TEBT+T	Current Sense Amplifier	IC3	1	-		2.499	2.0641	Digikey
MAX17291ANT+T	Boost Converter	IC6	1	-		2.069	1.6995	Digikey
BM02B-ACHSS-GAN-TF(LF)(SN)	Connector Header Surface Mount	J1	1	-		0.419	0.3557	Digikey
PMEG3010ER,115	Diode Schottky	J2	1	-		0.228	0.1435	Digikey
N/A, Exposed pads	Surface Mount Header Pin Pad	J3, J4	2	-	DNI	0	0	
5034802000	20 Position FFC, FPC Connector	J5	1	-		1.404	1.1931	Digikey
N/A, Exposed pads	Surface Mount Header Pin Pad	J7, J8	2	-	DNI	0	0	
MDBT50Q-1MV2	MDBT50Q_52840	M2	1	-		6.15	6.15	Digikey
CSD75208W1015	-20-V, P channel NexFET	Q1	1	-		0.473	0.3053	Digikey
DMP32D5SFB-7B	MOSFET (P-Channel)	Q2	1	-		0.277	0.175	Digikey
DMN62D1LFB-7B	MOSFET (N-Channel)	Q3	1	-		0.215	0.1347	Digikey
TF412ST5G	N-ch JFET Transistor	Q4	1	-		0.315	0.1998	Digikey
NANOT100AS	Smallest Push Button Switch	S1	1	-		0.358	0.2993	Digikey
AD5940BCBZ-RL	Electrochemical Front End	U1	1	-		10.66	9.4095	Digikey
TLV8542RUGR	Low Input Bias Current Opamp	U2, U4	2	-		0.942	0.7556	Digikey
TMUX1104DQAR	4:1 Analog MUX	U3	1	-		2.189	1.8016	Digikey
TPS7A0230PDQNR	Small Low Iq LDO	U6	1	-		0.536	0.4218	Digikey
MAX44264EWT+T	Small Low Iq Opamp	U8	1	-		1.485	1.2077	Digikey

**Total Unit Price per PCB ($USD) @ Quantity 10x :**	**43.13**
**Total Unit Price per PCB ($USD) @ Quantity 100x :**	**36.26**

Supplementary Table 9. Bill of materials for all components on the docking station PCB.

Manufacturer Part Number	Description	Designator	Quantity on PCB	Value	Do Not Install	Unit Price ($USD) (@ Quantity 10x)	Unit Price ($USD) (@ Quantity 100x)	Supplier
C0805C103J5RAC7025	Multilayer Chip Capacitor 0805	C3	1	10nF		0.06	0.0361	Digikey
08055C153KAT2A	Multilayer Chip Capacitor 0805	C4	1	15nF		0.072	0.0436	Digikey
C0805C104K5RAC7800	Multilayer Chip Capacitor 0805	C13, C14	2	100nF		0.016	0.016	Digikey
C0805F474K3RAC7800	Multilayer Chip Capacitor 0805	C5, C7, C8	3	470nF		0.294	0.1974	Digikey
C0805C106K9PAC7800	Multilayer Chip Capacitor 0805	C1, C2, C6, C9, C11, C12	6	10uF		0.188	0.188	Digikey
N/A	Multilayer Chip Capacitor 0805	C10	1	N/A	DNI	0	0	
RC0805JR-070RL	Surface Mount Resistor 0805	R11	1	0Ω		0.015	0.0097	Digikey
311-470ARTR-ND	Surface Mount Resistor 0805	R2	1	470Ω		0.009	0.0075	Digikey
RC0805FR-071KL	Surface Mount Resistor 0805	R6, R9	2	1kΩ		0.008	0.008	Digikey
RC0805FR-072KL	Surface Mount Resistor 0805	R8	1	2kΩ		0.009	0.0084	Digikey
RC0805FR-0710KL	Surface Mount Resistor 0805	R10, R13	2	10kΩ		0.014	0.0083	Digikey
RC0805FR-0720KL	Surface Mount Resistor 0805	R3	1	20kΩ		0.009	0.0084	Digikey
RC0805JR-07100KL	Surface Mount Resistor 0805	R5	1	100kΩ		0.009	0.0075	Digikey
RC0805FR-071ML	Surface Mount Resistor 0805	R7, R12	2	1MΩ		0.009	0.0082	Digikey
RMCF0805FT10M0	Surface Mount Resistor 0805	R1	1	10MΩ		0.008	0.008	Digikey
N/A	Surface Mount Resistor 0805	R4, R14, R15, R16	4	N/A	DNI	0	0	
5988130107F	Surface Mount LED 0805	D1	1	-		0.236	0.1667	Digikey
N/A	Through-hole LED footprint	D2, D3, D4	3	-	DNI	0	0	
1050170001	Conn Micro USB Type B RCP 5	J1	1	-		0.785	0.667	Digikey
NRPN031PAEN-RC	Pin Header 3x1	J2	1	-		0.31	0.2635	Digikey
MBR120ESFT3G	Diode Schottky 1A Surface Mount	J3	1	-		0.399	0.3145	Digikey
M20-9990246	Pin Header 2x1	J4, J5, J10, J12, J14, J15, J16, J19, J20, J21	10	-		0.079	0.0668	Digikey
855-22-010-10-001101	10 pin POGO pin header	J6, J8	2	-		8.374	7.415	Digikey
SHF-105-01-L-D-SM	Surface Mount Shrouded Header	J7	1	-		2.55	2.03	Digikey
61300411121	CONN HEADER VERT 4POS 2.54MM	J9	1	-	DNI	0	0	
855-22-008-10-001101	8 pin POGO pin header	J11, J13	2	-		7.835	6.86523	Digikey
DS1020-03ST1D	3 pin right angle SPE connector	J17, J18	2	-		0.107	0.0848	CONNFLY
1725708	Phoenix Screw Terminal Connector	J22, J23	2	-		4.459	3.9568	Digikey
S2B-PH-K-S(LF)(SN)	LiPo Battery JST Connector	J*	1	-		0.097	0.0821	Digikey
BLM21BD222TN1D	Surface Mount Inductor 0805	L1	1	Ferrite Bead		0.14	0.0973	Digikey
DMG2305UX-13	P-Channel MOSFET DMG2305UX-13	M1, M2	2	-		0.183	0.1145	Digikey
1101M2S3CQE2	Slide Switch SPDT Through Hole	S1	1	-		5.424	4.4836	Digikey
B3U-1000P	SWITCH TACTILE SPST-NO 0.05A 12V	S2	1	-	DNI	0	0	
N/A	Screw Hole	SC1, SC2, SC3, SC4	4	-	DNI	0	0	
5010	PC TEST POINT RED	TP1	1	-		0.256	0.1936	Digikey
5011	PC TEST POINT BLACK	TP3	1	-		0.238	0.19	Digikey
MCP73831T-2ACI/OT	Charger IC Lithium Ion/Polymer	U1	1	-		0.76	0.6125	Digikey
AP2171WG-7	Do Not Install	U2	1	-	DNI	0	0	
SPN02SXCN-RC	Jumper for J2 Header	N/A	1	-		0.056	0.0476	Digikey

**Total Unit Price per PCB ($USD) @ Quantity 10x :**	**56.25**
**Total Unit Price per PCB ($USD) @ Quantity 100x :**	**48.62**

Supplementary Table 10. Detailed cost breakdown of the complete Biocoin system at varying production volumes, and comparison with representative commercial potentiostat platforms. The table summarizes per-unit costs for the Biocoin wearable and docking station at order quantities of 10 and 100 units, including PCB components, fabrication, assembly, battery components, and 3D-printed fixtures. Component pricing is based on distributor quotes; fabrication and assembly costs are based on PCB Minions' turnkey manufacturing quotes; and 3D-printed fixture quotes were obtained from Xometry. For context, representative commercially available potentiostat systems are included to highlight the relative cost savings of the custom wearable, while noting differences in form factor and intended application.

**Full Biocoin System Cost Breakdown**		
**Item**	**Unit Price ($USD) (@ Quantity 10×)**	**Unit Price ($USD) (@ Quantity 100×)**
**Biocoin**		
PCB Components *	42.81	36.02
PCB Fabrication	92.31	26.00
PCB + Component Assembly	65.10	9.46
**Docking Station**		
PCB Components	56.25	48.62
PCB Fabrication	17.50	2.44
PCB + Component Assembly	48.30	7.69
**Battery Parts**		
110 mAh Li-Ion Battery (Qty. 1) (Part #: RJD2430C1ST1)	11.20	8.85
Battery Lead Receptacle (Qty. 1) (Part #: ACHR-02V-S)	0.087	0.0738
Battery Leads (Qty. 2) (Part #: ASACHSACH28W51)	0.531 × 2 = 1.062	0.4509 × 2 = 0.9018
**3D-Printed Fixtures**		
Biocoin Top Case	21.94	19.88
Biocoin Bottom Case	21.50	19.44
Docking Station Top Fixture	21.77	19.71
Docking Station Bottom Fixture	22.35	20.29
**Total Biocoin Cost**	422.18	219.38

**Comparison to Commercially Available Potentiostat Systems**		
**System**	**Unit Price ($USD)**	**Notes**
Biocoin (@ Quantity 10×)	422.18	Open-source Wearable Potentiostat
Biocoin (@ Quantity 100×)	219.38	Open-source Wearable Potentiostat
Sensit Wearable (PalmSens)	1,186	Commercial Wearable Potentiostat
Sensit BT (PalmSens)	1,833	Handheld Wireless Potentiostat
EmStat Pico Module (PalmSens)	599	Potentiostat Module Only (Additional hardware required)
PalmSens4 (PalmSens)	5,571	Portable Benchtop Potentiostat
Autolab DRP-STAT400	5,293	Portable Benchtop Potentiostat
Autolab PGSTAT204	9,497	Benchtop Potentiostat Workstation

* Using 3.6 V $V_{DD,A}$ configuration

Supplementary References

1. Yu, Y. *et al.* Biofuel-powered soft electronic skin with multiplexed and wireless sensing for human-machine interfaces. *Sci. Robot.* **5**, eaaz7946 (2020).
2. Song, Y. *et al.* Wireless battery-free wearable sweat sensor powered by human motion. *Sci. Adv.* **6**, eaay9842 (2020).
3. An, Z. *et al.* Body Heat Powered Wirelessly Wearable System for Real-time Physiological and Biochemical Monitoring. *Adv. Funct. Mater.* **33**, 2303361 (2023).
4. Liu, G. *et al.* High-output moisture-enabled electricity generator for fully self-powered wearable physical and biochemical monitoring. *Nano Energy* **119**, 109098 (2024).
5. Min, J. *et al.* An autonomous wearable biosensor powered by a perovskite solar cell. *Nat. Electron.* **6**, 630–641 (2023).
6. Liu, Y. *et al.* Stretchable Sweat-Activated Battery in Skin-Integrated Electronics for Continuous Wireless Sweat Monitoring. *Adv. Sci.* **9**, 2104635 (2022).
7. Mirlou, F. *et al.* Continuous Glycemic Monitoring Enabled by A Wi-Fi Energy-Harvesting Wearable Sweat-Sensing Patch. *Adv. Mater. Technol.* **n/a**, 2301583 (2024).
8. Cheng, C. *et al.* Battery-free, wireless, and flexible electrochemical patch for in situ analysis of sweat cortisol via near field communication. *Biosens. Bioelectron.* **172**, 112782 (2021).
9. Reeder, J. T. *et al.* Waterproof, electronics-enabled, epidermal microfluidic devices for sweat collection, biomarker analysis, and thermography in aquatic settings. *Sci. Adv.* **5**, eaau6356 (2019).
10. Shi, Z. *et al.* Wireless and battery-free wearable biosensing of riboflavin in sweat for precision nutrition. *Biosens. Bioelectron.* **251**, 116136 (2024).
11. Xu, G. *et al.* Battery-Free and Wireless Smart Wound Dressing for Wound Infection Monitoring and Electrically Controlled On-Demand Drug Delivery. *Adv. Funct. Mater.* **31**, 2100852 (2021).
12. Adams, S. D., Doeven, E. H., Quayle, K. & Kouzani, A. Z. MiniStat: Development and Evaluation of a Mini-Potentiostat for Electrochemical Measurements. *IEEE Access* **7**, 31903–31912 (2019).

13. Ainla, A. *et al.* Open-Source Potentiostat for Wireless Electrochemical Detection with Smartphones. *Anal. Chem.* **90**, 6240–6246 (2018).
14. Anshori, I. *et al.* ESPotensio: A Low-Cost and Portable Potentiostat With Multi-Channel and Multi-Analysis Electrochemical Measurements. *IEEE Access* **10**, 112578–112593 (2022).
15. Beck, J. J. *et al.* Evaluating the performance of an inexpensive, commercially available, NFC-powered and smartphone controlled potentiostat for electrochemical sensing. *Electroanalysis* **35**, e202200552 (2023).
16. Bill, D. *et al.* Electrochemical Methods in the Cloud: FreiStat, an IoT-Enabled Embedded Potentiostat. *Anal. Chem.* **95**, 13003–13009 (2023).
17. Caux, M. *et al.* PassStat, a simple but fast, precise and versatile open source potentiostat. *HardwareX* **11**, e00290 (2022).
18. Hoilett, O. S. *et al.* KickStat: A Coin-Sized Potentiostat for High-Resolution Electrochemical Analysis. *Sensors* **20**, 2407 (2020).
19. Ibrahim, N. F. A. *et al.* We-VoltamoStat: A wearable potentiostat for voltammetry analysis with a smartphone interface. *HardwareX* **15**, e00441 (2023).
20. Mercer, C., Bennett, R., Conghaile, P. Ó., Rusling, J. F. & Leech, D. Glucose biosensor based on open-source wireless microfluidic potentiostat. *Sens. Actuators B Chem.* **290**, 616–624 (2019).
21. Rowe, A. A. *et al.* CheapStat: An Open-Source, “Do-It-Yourself” Potentiostat for Analytical and Educational Applications. *PLOS ONE* **6**, e23783 (2011).
22. Sun, A., Venkatesh, A. G. & Hall, D. A. A Multi-Technique Reconfigurable Electrochemical Biosensor: Enabling Personal Health Monitoring in Mobile Devices. *IEEE Trans. Biomed. Circuits Syst.* **10**, 945–954 (2016).
23. Sun, A. C., Yao, C., A.g., V. & Hall, D. A. An efficient power harvesting mobile phone-based electrochemical biosensor for point-of-care health monitoring. *Sens. Actuators B Chem.* **235**, 126–135 (2016).
24. Dryden, M. D. M. & Wheeler, A. R. DStat: A Versatile, Open-Source Potentiostat for Electroanalysis and Integration. *PLoS ONE* **10**, e0140349 (2015).
25. Bobacka, J., Ivaska, A. & Lewenstam, A. Potentiometric Ion Sensors. *Chem. Rev.* **108**, 329–351 (2008).

26. Emaminejad, S. *et al.* Autonomous sweat extraction and analysis applied to cystic fibrosis and glucose monitoring using a fully integrated wearable platform. *Proc. Natl. Acad. Sci.* **114**, 4625–4630 (2017).

27. Hojaiji, H. *et al.* An autonomous wearable system for diurnal sweat biomarker data acquisition. *Lab. Chip* **20**, 4582–4591 (2020).

28. Kim, J. *et al.* Simultaneous Monitoring of Sweat and Interstitial Fluid Using a Single Wearable Biosensor Platform. *Adv. Sci.* **5**, 1800880 (2018).

29. Kim, J. *et al.* Noninvasive Alcohol Monitoring Using a Wearable Tattoo-Based Iontophoretic-Biosensing System. *ACS Sens.* **1**, 1011–1019 (2016).

30. Paul, B., Demuru, S., Lafaye, C., Saubade, M. & Briand, D. Printed Iontophoretic-Integrated Wearable Microfluidic Sweat-Sensing Patch for On-Demand Point-Of-Care Sweat Analysis. *Adv. Mater. Technol.* **6**, 2000910 (2021).

31. Roustit, M., Blaise, S. & Cracowski, J.-L. Trials and tribulations of skin iontophoresis in therapeutics. *Br. J. Clin. Pharmacol.* **77**, 63–71 (2014).

32. Wang, M. *et al.* A wearable electrochemical biosensor for the monitoring of metabolites and nutrients. *Nat. Biomed. Eng.* **6**, 1225–1235 (2022).

33. Xu, C. *et al.* A physicochemical-sensing electronic skin for stress response monitoring. *Nat. Electron.* **7**, 168–179 (2024).

34. Tu, J. *et al.* A wireless patch for the monitoring of C-reactive protein in sweat. *Nat. Biomed. Eng.* **7**, 1293–1306 (2023).

35. Saha, T. *et al.* Wireless Wearable Electrochemical Sensing Platform with Zero-Power Osmotic Sweat Extraction for Continuous Lactate Monitoring. *ACS Sens.* **7**, 2037–2048 (2022).

36. Mishra, R. K. *et al.* Continuous Opioid Monitoring along with Nerve Agents on a Wearable Microneedle Sensor Array. *J. Am. Chem. Soc.* **142**, 5991–5995 (2020).

37. Mishra, R. K. *et al.* Simultaneous detection of salivary Δ9-tetrahydrocannabinol and alcohol using a Wearable Electrochemical Ring Sensor. *Talanta* **211**, 120757 (2020).

38. Tai, L.-C. *et al.* Nicotine Monitoring with a Wearable Sweat Band. *ACS Sens.* **5**, 1831–1837 (2020).

39. Tai, L.-C. *et al.* Methylxanthine Drug Monitoring with Wearable Sweat Sensors. *Adv. Mater.* **30**, 1707442 (2018).

40. Ying, D. & Hall, D. A. Current Sensing Front-Ends: A Review and Design Guidance. *IEEE Sens. J.* **21**, 22329–22346 (2021).

41. Tehrani, F. *et al.* An integrated wearable microneedle array for the continuous monitoring of multiple biomarkers in interstitial fluid. *Nat. Biomed. Eng.* **6**, 1214–1224 (2022).

42. Madden, J., O'Mahony, C., Thompson, M., O'Riordan, A. & Galvin, P. Biosensing in dermal interstitial fluid using microneedle based electrochemical devices. *Sens. Bio-Sens. Res.* **29**, 100348 (2020).

43. Bretag, A. H. Synthetic interstial fluid for isolated mammalian tissue. *Life Sci.* **8**, 319–329 (1969).

44. Lin, H. *et al.* A programmable epidermal microfluidic valving system for wearable biofluid management and contextual biomarker analysis. *Nat. Commun.* **11**, 4405 (2020).

45. Yang, Y. *et al.* A laser-engraved wearable sensor for sensitive detection of uric acid and tyrosine in sweat. *Nat. Biotechnol.* **38**, 217–224 (2020).

46. Pei, X. *et al.* A quantitative, multimodal wearable bioelectronic device for comprehensive stress assessment and sub-classification. *Nat. Commun.* **17**, 1150 (2026).

47. Ding, S. *et al.* A fingertip-wearable microgrid system for autonomous energy management and metabolic monitoring. *Nat. Electron.* **7**, 788–799 (2024).

48. Gao, W. *et al.* Fully integrated wearable sensor arrays for multiplexed in situ perspiration analysis. *Nature* **529**, 509–514 (2016).

49. Yokus, M. A., Songkakul, T., Pozdin, V. A., Bozkurt, A. & Daniele, M. A. Wearable multiplexed biosensor system toward continuous monitoring of metabolites. *Biosens. Bioelectron.* **153**, 112038 (2020).

50. Gao, Y. *et al.* A flexible multiplexed immunosensor for point-of-care in situ wound monitoring. *Sci. Adv.* **7**, eabg9614 (2021).

51. Wang, B. *et al.* Wearable aptamer-field-effect transistor sensing system for noninvasive cortisol monitoring. *Sci. Adv.* **8**, eabk0967 (2022).

52. Shirzaei Sani, E. *et al.* A stretchable wireless wearable bioelectronic system for multiplexed monitoring and combination treatment of infected chronic wounds. *Sci. Adv.* **9**, eadf7388 (2023).
53. Ye, C. *et al.* A wearable aptamer nanobiosensor for non-invasive female hormone monitoring. *Nat. Nanotechnol.* **19**, 330–337 (2024).
54. Bai, J. *et al.* Coin-sized, fully integrated, and minimally invasive continuous glucose monitoring system based on organic electrochemical transistors. *Sci. Adv.* **10**, eadl1856 (2024).
55. Liu, Z. *et al.* Wearable Systems of Reconfigurable Microneedle Electrode Array for Subcutaneous Multiplexed Recording of Myoelectric and Electrochemical Signals. *Adv. Sci.* **12**, 2409075 (2025).
56. Tu, J. *et al.* Stressomic: A wearable microfluidic biosensor for dynamic profiling of multiple stress hormones in sweat. *Sci. Adv.* **11**, eadx6491 (2025).
57. Wei, Y. *et al.* Integrated individually addressable microneedle arrays for robust glucose monitoring and on-demand insulin releasing. *Microsyst. Nanoeng.* **11**, 211 (2025).
58. Sun, H. *et al.* Modularly-Assembled Smart Microneedle Platform for Machine Learning-Driven Personalized Health Monitoring. *Nano-Micro Lett.* **18**, 248 (2026).
59. Wang, X. *et al.* A fully integrative multimodal biosensing system for real-time assessment of hypoxic metabolism and pressure ulcers risk. *Mater. Today Electron.* **16**, 100212 (2026).
